# Forecasting the impact of means restriction on the suicide mortality rate in the Region of the Americas: an ecological modeling study

**DOI:** 10.1016/j.lana.2024.100831

**Published:** 2024-07-16

**Authors:** Shannon Lange, Kawon V. Kim, Huan Jiang, Kevin D. Shield, Jürgen Rehm, Anselm J.M. Hennis, Renato Oliveira e Souza

**Affiliations:** a Institute for Mental Health Policy Research, Centre for Addiction and Mental Health, 33 Ursula Franklin Street, Toronto, Ontario, M5S 2S1, Canada; b Campbell Family Mental Health Research Institute, Centre for Addiction and Mental Health, 250 College Street, Toronto, Ontario, M5T 1R8, Canada; c Department of Psychiatry, University of Toronto, 250 College Street, 8th Floor, Toronto, Ontario, M5T 1R8, Canada; d Institute of Medical Science, Faculty of Medicine, University of Toronto, Medical Sciences Building, 1 King's College Circle, Room 2374, Toronto, Ontario, M5S 1A8, Canada; e Dalla Lana School of Public Health, Health Sciences Building, 155 College Street, 6th Floor, Toronto, Ontario, M5T 3M7, Canada; f Program on Substance Abuse & WHO European Region Collaboration Centre, Public Health Agency of Catalonia, Roc Boronat Street 81 - 95, 08005, Barcelona, Catalonia, Spain; g Zentrum für Interdisziplinäre Suchtforschung (ZIS), Universitätsklinikum Hamburg-Eppendorf, Martinistraße 52, 20246, Hamburg, Germany; h Pan American Health Organization, 525 23rd St. NW, Washington, DC, 20037, USA

**Keywords:** Means restriction, Suicide mortality, Suicide prevention

## Abstract

**Background:**

The suicide mortality rate has been increasing in Region of the Americas, despite decreasing in all other World Health Organization (WHO) regions. Means restriction is an effective evidence-based intervention for suicide prevention. The objective of the current study was to estimate the impact of implementing national-level means restriction policies (i.e., firearm and pesticide restrictions) on the suicide mortality rate in the Region of the Americas.

**Methods:**

In this ecological modeling study, two counterfactual scenarios were investigated using sex-specific suicide mortality data from the WHO Global Health Estimates database for 2000 to 2019. Forecasted sex-specific age-standardized suicide mortality rates were then estimated for each country for 2020 to 2030. Counterfactual scenario 1 involved modeling the impact of a firearm or pesticide restriction implemented in 2020 for those countries where the respective means accounted for 40% or more of all suicides for at least one sex in 2019, while in counterfactual scenario 2 this threshold was reduced to 20% or more.

**Findings:**

It was estimated that if a firearm or pesticide restriction had been implemented in 2020 in those countries where the respective means accounted for 40% or more of all suicides for at least one sex in 2019, by 2030 the male and female suicide mortality rate in the Region of the Americas would be 20.5% (from 14.5 [95% Confidence Interval [CI]: 14.1, 15.0] per 100,000 males to 11.5 [95% CI: 11.1, 12.0] per 100,000 males) and 11.1% (from 4.5 [95% CI: 4.4, 4.7] per 100,000 females to 4.0 [95% CI: 3.9, 4.2] per 100,000 females) lower than the rate if no such restrictions were implemented, respectively. When the threshold was reduced to 20% or more, minimal additional gains in terms of number of suicides avoided and suicide mortality rate reduction would be achieved.

**Interpretation:**

The implementation of a firearm or pesticide restriction policy in countries where the respective means account for a large proportion of suicides (e.g., at least 40%) could aid the Region of the Americas in achieving the WHO target of a one third reduction in the suicide mortality rate by 2030.

**Funding:**

This work received no funding.

**Research in context**

**Evidence before this study**

Means restriction–that is, limiting access to the means of suicide–has been identified as an effective evidence-based intervention for suicide prevention. Two means of suicide for which restrictions can be implemented at a nation-level are firearms and pesticides. We searched Medline, Embase, PsycInfo, CINAHL, Scopus, Cochrane, and DARE with no date or language restrictions up to May 8, 2023, for published studies evaluating the impact of national-level means restriction policies on the sex-specific suicide mortality rate. Using search terms to cover “means restriction or ban” and “suicide or suicide prevention”, and applying the criterion that the restriction was implemented on a national-level, a total of 15 studies were identified. Taken together, firearm and pesticide restrictions appear to be an effective means of reducing firearm- and pesticide-involved suicides, respectively. Thus, based on the available literature, it is likely that firearm and pesticide restrictions could aid in reducing the suicide mortality rate within the Region of the Americas, which has been increasing in recent years, despite decreasing in all other World Health Organization (WHO) regions.

**Added value of this study**

Our findings provide tangible estimates of the potential impact that firearm and pesticide restrictions would have on the suicide mortality rate in the Region of the Americas, if firearm and pesticide restriction policies were implemented in countries where the respective means accounted for 40% or more of their overall suicides. To the best of our knowledge, this is the first study to model the impact of firearm and pesticide restrictions on the suicide mortality rate on a country, sub-regional, and regional level for the Region of the Americas, or elsewhere.

**Implications of all the available evidence**

Means restriction policies that can be implemented at a national-level are a way in which the Region of the Americas could counter its current suicide mortality rate trend, and aid in achieving the WHO target of a one third reduction in the suicide mortality rate by 2030. Comprehensive suicide prevention strategies should include means restriction policies wherever it is appropriate to do so, and this determination must be guided by a situation analysis of the local epidemiology of suicide.

## Introduction

Reducing the global suicide mortality rate by one third by 2030 is an explicit goal highlighted in the World Health Organization's (WHO) Comprehensive Mental Health Action Plan 2013–2030.^1^ The suicide mortality rate is also an indicator for the United Nations Sustainable Development Goals (SDG) target 3.4 (Target 3.4 is: By 2030, reduce by one third premature mortality from non-communicable diseases through prevention and treatment and promote mental health and well-being)^2^; recognizing its importance as an indicator of a population's mental health.

Despite decreasing in all other WHO regions between 2000 and 2019, the suicide mortality rate in the Region of the Americas increased; highlighting the urgent need for enhanced prevention efforts in this particular region.^3^ In 2019, over 97 thousand individuals died by suicide in the Region of the Americas, resulting in a suicide mortality rate of 9.0 per 100,000 population (14.2 per 100,000 males and 4.1 per 100,000 females).^4^

The WHO advocates for countries to take action to prevent suicide, ideally through a comprehensive national suicide prevention strategy. Governments and communities can contribute to suicide prevention by implementing LIVE LIFE–the WHOs recommended approach to suicide prevention, intended to be used as a starting point for countries, which can be built on with the development of a comprehensive prevention strategy.^5^ LIVE LIFE includes four effective evidence-based interventions for suicide prevention: 1) Limit access to the means of suicide (so-called means restriction); 2) Interact with the media for responsible reporting of suicide; 3) Foster socio-emotional life skills in adolescents; and 4) Early identify, assess, manage and follow up any one who is affected by suicide behaviors. Of these four recommended interventions, means restriction would be expected to have an immediate impact on the suicide mortality rate in a given country, whereas the latter three would likely have a lagged effect.

However, not all means of suicide can be targeted with such strategies. For instance, suicide by asphyxiation is carried out using a variety of readily available means. Therefore, prevention of suicides that involve means that cannot be realistically targeted by restricting access should focus on the other three LIVE LIFE interventions. Further, some forms of means restriction would be more appropriate to do in certain settings (e.g., eliminate hanging structures within institutional settings) or locations (e.g., a barrier installed at a cliff where a large number of individuals have jumped from), and although effective in reducing the number of suicides in the respective setting/location, they are unlikely to make a meaningful difference in the national suicide rate.^6^ The exception to such caveats would be firearm and pesticide restrictions, both of which can be done on a national-level and have been shown to be effective in reducing the suicide mortality rate.^7^, ^8^, ^9^, ^10^ In the current study, we aimed to estimate the impact of implementing firearm and pesticide restrictions in those countries where the respective means accounted for a large proportion of suicides on the suicide mortality rate in the Region of the Americas.

## Methods

An ecological study design was used to estimate the impact of firearm and pesticide restrictions on the sex-specific suicide mortality rate in the Region of the Americas. This study is reported according to the Strengthening the Reporting of Observational Studies in Epidemiology (STROBE) statement (https://www.equator-network.org/reporting-guidelines/strobe/; Supplementary Table S1).

### Data and definitions

Annual sex-specific age-standardized suicide mortality rate estimates were obtained from the WHO Global Health Estimates database (https://www.who.int) for a total of 33 countries of the Region of the Americas for 2000 to 2019: Antigua and Barbuda, Argentina, Bahamas, Barbados, Belize, Bolivia (Plurinational State of), Brazil, Canada, Chile, Colombia, Costa Rica, Cuba, Dominican Republic, Ecuador, El Salvador, Grenada, Guatemala, Guyana, Haiti, Honduras, Jamaica, Mexico, Nicaragua, Panama, Paraguay, Peru, Saint Lucia, Saint Vincent and the Grenadines, Suriname, Trinidad and Tobago, United States of America, Uruguay, and Venezuela (Bolivarian Republic of). The suicide mortality rate estimates are derived from primary data, and when absent, estimates are derived from statistical modeling. In brief, total deaths by 5-year age groups and sex were estimated by the WHO for each country by applying the WHO life table death rates^11^ to the estimated resident populations prepared by the UN Population Division in its 2019 revision.^12^ Age-standardized suicide mortality rates were computed using the WHO standard population,^13^ which assumes one standard age distribution of the population in all countries. Additional details of the methods used by the WHO to compute the suicide mortality rate estimates used here, as well as data sources can be found elsewhere.^14^

The term pesticide restriction herein refers specifically to a ban on highly hazardous pesticides specifically, while the term firearm restriction herein refers to access restrictions (i.e., a ban of semi-automatic and fully automatic weapons for “personal protection”, an act which is often paired with a “buyback” provision).

### Statistical analysis

Estimating the impact of firearm and pesticide restrictions on suicide mortality was done using the following steps.

#### Forecast suicide mortality rates until 2030

Joinpoint regression analyses were performed on the country and sex-specific age-standardized suicide mortality rates to visualize and describe each time-series (Supplementary Tables S2–S7 and Supplementary Figures S1–S12). Forecasted country and sex-specific age-standardized suicide mortality rates were then estimated by fitting either a linear model or autoregressive integrated moving average (ARIMA) model to the 2000 to 2019 suicide mortality rates and extrapolated to obtain 2020 to 2030 suicide mortality rates. If the linear model or ARIMA model were a poor fit, as per the joinpoint regressions–i.e., did not visually align with the last segment identified, the locally weighted scatterplot smoothing (LOWESS) smoother with a span equal to 0.2 was applied and the values obtained from the smooth function were fitted to an ARIMA model to forecast 2020 to 2030 suicide mortality rates. See Supplementary Tables S8 and S9 for the observed and forecasted country and sex-specific age-standardized suicide mortality rates from 2000 to 2030, and Supplementary Table S10 for the model fit statistics of the forecasting models.

It was decided a priori that countries with a suicide mortality rate less than three per 100,000 population in 2019 would not be included in the counterfactual scenario estimates, regardless of the proportions of firearm- and pesticide-involved suicides, as it is not realistic that a country with such a low rate would necessarily implement a suicide means restriction policy. The following countries had a suicide mortality rate less than three per 100,000 population in 2019: Antigua and Barbuda, Barbados, Grenada, Honduras, Jamaica, Panama, Peru, Saint Vincent and the Grenadines, and Venezuela.^4^ It should be noted that the forecasted suicide mortality rates of the respective countries were included in the sub-regional and regional estimates.

#### Separate suicide mortality rates into mean-specific rates

The sex-specific proportion of firearm- and pesticide-involved suicides in each country was obtained from the Pan American Health Organization,^15^ and was applied to the forecasted country and sex-specific age-standardized suicide mortality rates for 2020 to 2030; resulting in firearm- and pesticide-involved suicide mortality rates, and non-firearm- and non-pesticide-involved suicide mortality rates. The proportion of firearm- and pesticide-involved suicides were obtained for 2019, or the latest available year (Supplementary Table S11). Data on the sex-specific proportion of firearm- and pesticide-involved suicides in Bahamas, Bolivia, Haiti, and Saint Lucia were not available. Thus, it was not possible to model a means restriction implementation for the respective countries; however, the forecasted suicide mortality rates were included in the sub-regional and regional estimates.

#### Obtain effect estimate for the impact of means restriction on the suicide mortality rate

We performed a systematic literature search in PubMed, Embase and PsycInfo from database inception to 8 May 2023, using the following keywords: policy or public policy or health policy; and pesticides or firearms; and suicide or suicide prevention (see Supplementary Table S12 for search strategy). After deduplication, records were screened by two reviewers independently using the population, intervention, outcome, comparator, study design criteria for study selection (Supplementary Table S13). Ten studies investigating the effects of a national firearm restriction on the suicide mortality rate^7^^,^^8^^,^^16^, ^17^, ^18^, ^19^, ^20^, ^21^, ^22^, ^23^ and five studies investigating the effects of a national pesticide restriction on the suicide mortality rate^9^^,^^10^^,^^24^, ^25^, ^26^ were identified. We then applied the following inclusion criteria, the study must provide the 1) rate ratio–the ratio of the suicide mortality rate pre- and annual post-intervention, 2) impact of the respective restriction on the mean-specific and non-mean-specific suicide mortality rate (e.g., firearm and non-firearm suicide mortality rate), and 3) corresponding measure of variability or sufficient data to calculate these (e.g., number of deaths). The criterion that the intervention effect had to be reported for both the mean specific and non-mean-specific suicide mortality rate allowed us to account for the substitution effect (i.e., when one means of suicide is unavailable, another means might be used instead–rather than the act being abandoned altogether). In addition to the above inclusion criteria, for studies on the impact of a pesticide restriction on suicide mortality, the study with the most encompassing ban was selected. Accordingly, two studies were retained (one for each type of ban; Supplementary Figure S13) and their reported rate ratios were used in the estimations described below: an Australian study investigating the impact of the 1996 National Firearms Agreement on the suicide mortality rate,^7^ and a study of the effects of a phased national-ban on 21 WHO Class I pesticides on the suicide mortality rate in Bangladesh^10^ (see Supplementary Tables S14 and S15).

#### Estimate the counterfactual scenarios of means restriction on the suicide mortality rate

Given that the impact of a means restriction policy on the overall suicide mortality rate is dependent on how common the targeted suicide means is, two counterfactual scenarios differing in the relative proportion of suicides involving the respective means were modeled (i.e., varying the commonness of the respective means).

1. A means restriction (firearm or pesticide restriction) for those countries where the respective means accounted for 40% or more of all suicides for at least one sex.

Counterfactual scenario 1 included the implementation of a firearm restriction in the United States, and a pesticide restriction in El Salvador, Guyana, Nicaragua, and Suriname (i.e., those countries where firearm- or pesticide-involved suicide accounted for at least 40% of overall suicides for at least one sex).

2. A means restriction (firearm or pesticide restriction) for those countries where the respective means accounted for 20% or more of all suicides for at least one sex.

Counterfactual scenario 2 included the implementation of a firearm restriction in the United States and Uruguay, and a pesticide restriction in El Salvador, Guyana, Nicaragua, Suriname and Trinidad and Tobago (i.e., those countries where firearm- or pesticide-involved suicide accounted for at least 20% of overall suicides for at least one sex). It should be noted that Guatemala met the criteria for counterfactual scenario 2; however, the forecasted values for males fell below zero and therefore, was not included.

Annual rate ratio estimates, which are the ratio of the suicide mortality rate pre- and annual post-intervention (i.e., firearm or pesticide restriction), were obtained from the existing literature.^7^^,^^10^ The corresponding 95% confidence intervals (CI), obtained by probabilistic sampling from distributions of suicide mortality rates, were used as a measure of uncertainty. Assuming that the bans were implemented in 2020, the firearm- or pesticide-involved suicide mortality rates and the non-firearm- and non-pesticide-involved suicide mortality rates were multiplied by the respective annual rate ratio (i.e., the mean-specific rate ratio–firearm- involved and non-firearm involved suicides, and pesticide-involved and non-pesticide involved suicides, for firearm and pesticide restrictions, respectively–of the first-year post-intervention was applied to the 2021 country- and mean-specific suicide mortality rate, and so on). The combined total of these two categories provides an estimate of the suicide mortality rate that would occur under a counterfactual firearm or pesticide restriction.

#### Estimate sub-regional and regional rates and absolute number of avoided deaths by suicide

The estimation of the number of deaths from 2020 to 2030 were based upon on population data obtained from the United Nations,^27^ and the projections for age-standardized mortality rates. The method to estimate deaths from 2020 to 2030 assumed that the percentage change in age-standardized mortality rates mirrored the percentage shift in age-specific mortality rates. The sub-regional and regional rates were weighted by population size. Monte Carlo simulations were conducted to estimate the distribution for the absolute number of avoided suicides, generating 1000 samples, and using the 2.5th and 97.5th percentiles of the resulting distribution as the uncertainty interval (UI). For countries where the forecasted values fell below zero, a value of zero was imputed.

All analyses were performed using R version 4.2.1.

### Ethical statement

Institutional review board approval and informed consent were not required, as the current study utilized aggregated data obtained from public domain databases.

### Role of the funding source

This research received no funding.

## Results

### Counterfactual scenario 1

**Fig. 1:**
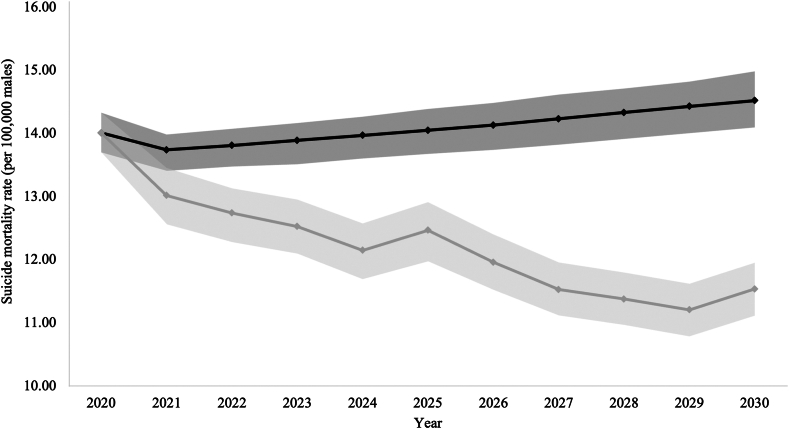
Predicted age-standardized suicide mortality rate per 100,000 males in the Region of the Americas if no means restrictions were implemented and under counterfactual scenario 1^a^, 2020–2030 ^a^A means restriction (firearm or pesticide restriction) implemented in 2020 for those countries where the respective means accounted for 40% or more of all suicides for at least one sex. *Note.* The scenario of no restrictions is depicted by the black solid line and data points; counterfactual scenario 1 is depicted by the grey solid line and data points.

**Fig. 2:**
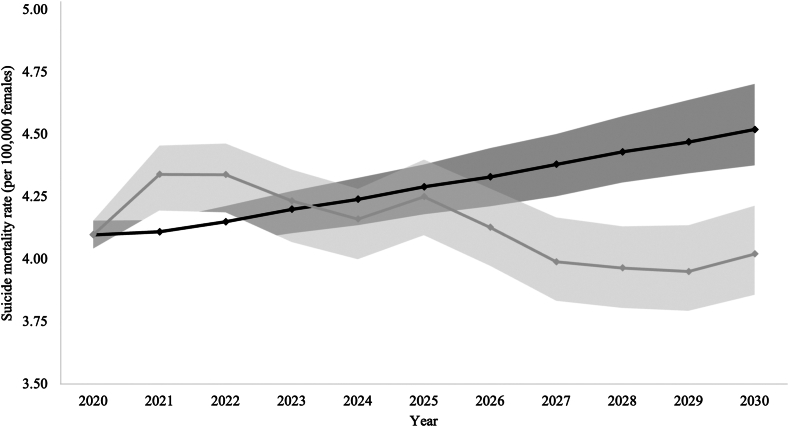
Predicted age-standardized suicide mortality rate per 100,000 females in the Region of the Americas if no means restrictions were implemented and under counterfactual scenario 1^a^, 2020–2030 ^a^A means restriction (firearm or pesticide restriction) implemented in 2020 for those countries where the respective means accounted for 40% or more of all suicides for at least one sex. *Note.* The scenario of no restrictions is depicted by the black solid line and data points; counterfactual scenario 1 is depicted by the grey solid line and data points.

It was estimated that if a firearm or pesticide restriction were implemented in 2020 in those countries where the respective means accounted for more than 40% of all suicides for at least one sex in 2019, a total of 113,580 (95% UI: −145,477, −79,637) deaths by suicide (−103,979 (95% UI: −133,554, −72,410) males and 9601 (95% UI: −21,203, 2097) females) could have been avoided by 2030 in the Region of the Americas (i.e., over the course of ten years; Table 1). By 2030 under counterfactual scenario 1, the male and female suicide mortality rate in the Region of the Americas was estimated to be 20.5% (from 14.5 [95% CI: 14.1, 15.0] per 100,000 males to 11.5 [95% CI: 11.1, 12.0] per 100,000 males; Fig. 1) and 11.1% (from 4.5 [95% CI: 4.4, 4.7] per 100,000 females to 4.0 [95% CI: 3.9, 4.2] per 100,000 females; Fig. 2) lower, respectively, than the rate if no such restrictions were implemented.

**Table 1:** Predicted age-standardized suicide mortality rate per 100,000 population and number of avoided deaths by suicide in the Region of the Americas and its subregions under counterfactual scenario 1^a^ for the years 2021–2030.

Sub-region	Sex	Scenario	2021	2022	2023	2024	2025	2026	2027	2028	2029	2030
Andean Area^b^	Males	No restriction, age-standardized rate (95% CI)	5.6 (5.3, 5.9)	5.5 (5.1, 5.9)	5.4 (5.0, 5.9)	5.4 (4.9, 5.8)	5.3 (4.8, 5.8)	5.2 (4.8, 5.8)	5.2 (4.7, 5.7)	5.2 (4.7, 5.7)	5.1 (4.6, 5.7)	5.0 (4.5, 5.6)
	Females	No restriction, age-standardized rate (95% CI)	1.8 (1.7, 1.9)	1.7 (1.6, 1.9)	1.7 (1.5, 1.9)	1.7 (1.4, 2.0)	1.6 (1.3, 2.0)	1.6 (1.3, 2.0)	1.6 (1.2, 2.1)	1.5 (1.2, 2.2)	1.5 (1.2, 2.2)	1.5 (1.1, 2.3)
Central America, Mexico and Latin Caribbean^c^	Males	No restriction, age-standardized rate (95% CI)	9.7 (9.3, 10.1)	9.7 (9.2, 10.1)	9.6 (9.2, 10.0)	9.6 (9.1, 10.1)	9.6 (9.1, 10.0)	9.5 (9.1, 10.1)	9.6 (9.1, 10.1)	9.6 (9.1, 10.2)	9.6 (9.1, 10.2)	9.7 (9.1, 10.3)
		Means restriction, age-standardized rate (95% CI)	9.6 (9.2, 10.0)	9.5 (9.1, 10.0)	9.5 (9.1, 9.9)	9.5 (9.0, 9.9)	9.4 (8.9, 9.9)	9.4 (9.0, 9.9)	9.4 (8.9, 9.9)	9.4 (8.9, 10.0)	9.4 (8.9, 10.1)	9.4 (8.9, 10.1)
		Absolute number of avoided suicides (95% UI)	−72 (−614, 469)	−140 (−746, 429)	−166 (−802, 506)	−161 (−832, 536)	−171 (−832, 614)	−145 (−899, 609)	−165 (−954, 642)	−203 (−1,018, 671)	−213 (−1,162, 769)	−234 (−1,226, 685)
	Females	No restriction, age-standardized rate (95% CI)	2.7 (2.5, 2.9)	2.7 (2.5, 2.9)	2.7 (2.5, 3.0)	2.7 (2.4, 3.0)	2.7 (2.4, 3.1)	2.7 (2.4, 3.1)	2.7 (2.5, 3.1)	2.7 (2.4, 3.2)	2.7 (2.4, 3.3)	2.8 (2.4, 3.3)
		Means restriction, age-standardized rate (95% CI)	2.7 (2.5, 2.8)	2.7 (2.5, 2.9)	2.7 (2.4, 2.9)	2.7 (2.4, 3.0)	2.7 (2.4, 3.0)	2.7 (2.4, 3.1)	2.7 (2.4, 3.1)	2.7 (2.4, 3.2)	2.7 (2.4, 3.3)	2.7 (2.4, 3.3)
		Absolute number of avoided suicides (95% UI)	−16 (−236, 208)	−29 (−279, 237)	−33 (−360, 278)	−30 (−401, 354)	−29 (−440, 383)	−24 (−517, 427)	−27 (−556, 493)	−31 (−597, 508)	−32 (−629, 527)	−34 (−685, 614)
Non-Latin Caribbean^d^	Males	No restriction, age-standardized rate (95% CI)	14.3 (13.3, 15.3)	14.1 (13.1, 15.3)	13.9 (12.9, 15.1)	13.8 (12.7, 15.1)	13.8 (12.7, 15.1)	13.7 (12.6, 15.1)	13.6 (12.4, 14.9)	13.5 (12.4, 14.9)	13.5 (12.3, 14.9)	13.4 (12.3, 14.9)
		Means restriction, age-standardized rate (95% CI)	13.1 (12.7, 13.8)	11.8 (11.3, 12.5)	11.3 (10.7, 12.0)	11.4 (10.8, 12.1)	11.3 (10.6, 12.1)	11.5 (10.9, 12.5)	11.1 (10.3, 12.0)	10.4 (9.6, 11.3)	10.1 (9.3, 11.1)	9.6 (8.8, 10.6)
		Absolute number of avoided suicides (95% UI)	−44 (−90, −5)	−86 (−130, −40)	−100 (−151, −48)	−93 (−146, −39)	−94 (−147, −45)	−81 (−133, −27)	−96 (−153, −36)	−119 (−177, −62)	−128 (−188, −71)	−144 (−208, −88)
	Females	No restriction, age-standardized rate (95% CI)	4.1 (3.8, 4.3)	4.0 (3.8, 4.3)	4.0 (3.8, 4.4)	4.1 (3.7, 4.4)	4.1 (3.7, 4.4)	4.1 (3.7, 4.5)	4.1 (3.6, 4.5)	4.1 (3.6, 4.5)	4.1 (3.6, 4.5)	4.1 (3.6, 4.6)
		Means restriction, age-standardized rate (95% CI)	3.7 (3.5, 3.9)	3.4 (3.1, 3.6)	3.2 (3.0, 3.5)	3.3 (3.0, 3.6)	3.3 (3.0, 3.7)	3.4 (3.0, 3.8)	3.3 (2.9, 3.6)	3.1 (2.7, 3.5)	3.0 (2.6, 3.4)	2.8 (2.4, 3.2)
		Absolute number of avoided suicides (95% UI)	−13 (−25, −1)	−26 (−39, −11)	−31 (−46, −16)	−28 (−45, −10)	−27 (−46, −9)	−24 (−44, −6)	−29 (−50, −9)	−37 (−58, −16)	−40 (−63, −18)	−46 (−68, −22)
North American^e^	Males	No restriction, age-standardized rate (95% CI)	21.5 (20.8, 22.2)	21.8 (21.1, 22.5)	22.0 (21.2, 22.7)	22.3 (21.5, 23.0)	22.6 (21.8, 23.4)	22.8 (22.0, 23.7)	23.1 (22.2, 24.0)	23.4 (22.4, 24.3)	23.6 (22.6, 24.6)	23.9 (22.8, 25.0)
		Means restriction, age-standardized rate (95% CI)	19.6 (18.5, 20.7)	18.9 (17.8, 20.0)	18.4 (17.4, 19.6)	17.4 (16.3, 18.5)	18.3 (17.1, 19.5)	16.9 (15.8, 18.0)	15.7 (14.7, 16.7)	15.2 (14.2, 16.3)	14.7 (13.8, 15.7)	15.7 (14.7, 16.7)
		Absolute number of avoided suicides (95% UI)	−3432 (−6046, −603)	−5102 (−7445, −2347)	−6538 (−9118,−3945)	−8855 (−11,314, −6161)	−7655 (−10,388,−4962)	−10,690 (−13,335,−7963)	−13,397 (−16,086,−10,653)	−14,665 (−17,278,−11,869)	−16,077 (−18,581,−13,266)	−14,911 (−17,622,−11,965)
	Females	No restriction, age-standardized rate (95% CI)	6.9 (6.7, 7.0)	6.9 (6.7, 7.0)	7.0 (6.8, 7.1)	7.1 (7.0, 7.3)	7.2 (7.1, 7.4)	7.4 (7.2, 7.5)	7.5 (7.3, 7.7)	7.6 (7.4, 7.8)	7.8 (7.5, 7.9)	7.9 (7.7, 8.1)
		Means restriction, age-standardized rate (95% CI)	7.4 (7.1, 7.8)	7.4 (7.0, 7.8)	7.1 (6.8, 7.5)	6.9 (6.6, 7.2)	7.2 (6.8, 7.5)	6.8 (6.5, 7.2)	6.4 (6.1, 6.7)	6.3 (6.0, 6.6)	6.3 (5.9, 6.6)	6.5 (6.1, 6.8)
		Absolute number of avoided suicides (95% UI)	1272 (464, 2018)	1055 (158, 1863)	255 (−615, 1085)	−386 (−1,211, 399)	−147 (−979, 743)	−1065 (−1,883, −207)	−2065 (−2,825, −1226)	−2457 (−3,256, −1604)	−2802 (−3,550, −1991)	−2673 (−3,467, −1742)
Southern Cone^b^	Males	No restriction, age-standardized rate (95% CI)	10.9 (10.6, 11.3)	11.0 (10.6, 11.3)	11.1 (10.6, 11.5)	11.1 (10.6, 11.6)	11.1 (10.7, 11.7)	11.3 (10.7, 11.8)	11.3 (10.7, 11.9)	11.4 (10.8, 12.0)	11.4 (10.8, 12.1)	11.5 (10.9, 12.2)
	Females	No restriction, age-standardized rate (95% CI)	3.0 (3.0, 3.1)	3.1 (3.0, 3.2)	3.1 (3.0, 3.2)	3.2 (3.0, 3.3)	3.2 (3.0, 3.3)	3.2 (3.1, 3.4)	3.3 (3.1, 3.4)	3.3 (3.1, 3.5)	3.3 (3.1, 3.5)	3.4 (3.1, 3.6)
Region of the Americas	Males	No restriction, age-standardized rate (95% CI)	13.7 (13.4, 14.0)	13.8 (13.5, 14.1)	13.9 (13.5, 14.2)	14.0 (13.6, 14.3)	14.1 (13.7, 14.4)	14.1 (13.7, 14.5)	14.2 (13.8, 14.6)	14.3 (13.9, 14.7)	14.4 (14.0, 14.8)	14.5 (14.1, 15.0)
		Means restriction, age-standardized rate (95% CI)	13.0 (12.6, 13.5)	12.7 (12.3, 13.1)	12.5 (12.1, 13.0)	12.1 (11.7, 12.6)	12.5 (12.0, 12.9)	12.0 (11.5, 12.4)	11.5 (11.1, 12.0)	11.4 (11.0, 11.8)	11.2 (10.8, 11.6)	11.5 (11.1, 12.0)
		Absolute number of avoided suicides (95% UI)	−3549 (−6282,−519)	−5328 (−7827,−2144	−6805 (−9763,−3891)	−9108 (−11,997,−6035)	−7921 (−10,854,−4565)	−10,917 (−13,909,−7853)	−13,658 (−16,839,−10,506)	−14,987 (−18,262,−11,872)	−16,417 (−19,365,−13,247)	−15,290 (−18,456, −11,777)
	Females	No restriction, age-standardized rate (95% CI)	4.1 (4.0, 4.2)	4.2 (4.1, 4.2)	4.2 (4.1, 4.3)	4.2 (4.1, 4.3)	4.3 (4.2, 4.4)	4.3 (4.2, 4.5)	4.4 (4.3, 4.5)	4.4 (4.3, 4.6)	4.5 (4.3, 4.6)	4.5 (4.4, 4.7)
		Means restriction, age-standardized rate (95% CI)	4.3 (4.2, 4.5)	4.3 (4.2, 4.5)	4.2 (4.1, 4.4)	4.2 (4.0, 4.3)	4.3 (4.1, 4.4)	4.1 (4.0, 4.3)	4.0 (3.8, 4.2)	4.0 (3.8, 4.1)	4.0 (3.8, 4.1)	4.0 (3.9, 4.2)
		Absolute number of avoided suicides (95% UI)	1243 (302, 2124)	1000 (−4, 1950)	191 (−848, 1220)	−444 (−1,600, 586)	−203 (−1,338, 1059)	−1113 (−2,285, 58)	−2121 (−3,349, −891)	−2526 (−3,794, −1212)	−2874 (−4,190, −1493)	−2754 (−4,069, −1303)

a A means restriction (firearm or pesticide restriction) implemented in 2020 for those countries where the respective means accounted for 40% or more of all suicides for at least one sex.

b No country within this subregion had firearms or pesticides account for 40% or more of all suicides for at least one sex.

c Pesticide restriction modeled for El Salvador and Nicaragua.

d Pesticide restriction modeled for Guyana and Suriname.

e Firearm restriction modeled for the United States of America.

For counterfactual scenario 1, three sub-regions would be impacted by a firearm or pesticide restriction implemented in 2020 (i.e., they include countries where the respective means accounted for more than 40% of all suicides for at least one sex in 2019): Central America, Mexico and Latin Caribbean, Non-Latin Caribbean, and North America. Of the three sub-regions, the most notable impacts would be in Non-Latin Caribbean and North America. By 2030 under counterfactual scenario 1, the male suicide mortality rate in Non-Latin Caribbean and North America would be 28.7% (from 13.4 [95% CI: 12.3, 14.9] per 100,000 males to 9.6 [95% CI: 8.8, 10.6] per 100,000 males) and 34.5% (from 23.9 [95% CI: 22.8, 25.0] per 100,000 males to 15.7 [95% CI: 14.7, 16.7] per 100,000 males) lower, respectively, than the rate if no such restrictions were implemented. While for females, the suicide mortality rate in Non-Latin Caribbean and North America would be 31.0% (from 4.1 [95% CI: 3.6, 4.6] per 100,000 females to 2.8 [95% CI: 2.4, 3.2] per 100,000 females) and 18.0% (from 7.9 [95% CI: 7.7, 8.1] per 100,000 females to 6.5 [95% CI: 6.1, 6.8] per 100,000 females) lower, respectively, than the rate if no such restrictions were implemented.

### Counterfactual scenario 2

It was estimated that if a firearm or pesticide restriction were implemented in 2020 in those countries where the respective means accounted for more than 20% of all suicides for at least one sex in 2019, a total of 113,649 (95% UI: −145,914, −79,386) deaths by suicide (−104,108 (95% UI: −134,046, −71,869) males and −9540 (95% UI: −21,233, 2050) females) could have been avoided by 2030 in the Region of the Americas (i.e., over the course of ten years Table 2). That is, by 2030 under counterfactual scenario 2, the male and female suicide mortality rate in the Region of the Americas was estimated to be 20.7% (from 14.5 [95% CI: 14.1, 15.0] per 100,000 males to 11.5 [95% CI: 11.1, 12.0] per 100,000 males) and 11.7% (from 4.5 [95% CI: 4.4, 4.7] per 100,000 females to 4.0 [95% CI: 3.9, 4.2] per 100,000 females) lower than the rate if no such restrictions were implemented.

**Table 2:** Predicted age-standardized suicide mortality rate per 100,000 population and number of avoided deaths by suicide in the Region of the Americas and its subregions under counterfactual scenario 2^a^ for the years 2021–2030.

Sub-region	Sex	Scenario	2021	2022	2023	2024	2025	2026	2027	2028	2029	2030
Andean Area^b^	Males	No restriction, age-standardized rate (95% CI)	5.6 (5.3, 5.9)	5.5 (5.1, 5.9)	5.4 (5.0, 5.9)	5.4 (4.9, 5.8)	5.3 (4.8, 5.8)	5.2 (4.8, 5.8)	5.2 (4.7, 5.7)	5.2 (4.7, 5.7)	5.1 (4.6, 5.7)	5.0 (4.5, 5.6)
	Females	No restriction, age-standardized rate (95% CI)	1.8 (1.7, 1.9)	1.8 (1.6, 1.9)	1.7 (1.5, 1.9)	1.7 (1.4, 2.0)	1.6 (1.3, 2.0)	1.6 (1.3, 2.0)	1.6 (1.2, 2.1)	1.5 (1.2, 2.2)	1.5 (1.2, 2.2)	1.5 (1.1, 2.3)
Central America, Mexico and Latin Caribbean^c^	Males	No restriction, age-standardized rate (95% CI)	9.7 (9.3, 10.1)	9.7 (9.2, 10.1)	9.6 (9.2, 10.0)	9.6 (9.1, 10.1)	9.6 (9.1, 10.0)	9.5 (9.1, 10.1)	9.6 (9.1, 10.1)	9.6 (9.1, 10.2)	9.6 (9.1, 10.2)	9.7 (9.1, 10.3)
		Means restriction, age-standardized rate (95% CI)	9.6 (9.2, 10.0)	9.5 (9.1, 10.0)	9.5 (9.1, 9.9)	9.5 (9.0, 9.9)	9.4 (8.9, 9.9)	9.4 (9.0, 9.9)	9.4 (8.9, 9.9)	9.4 (8.9, 10.0)	9.4 (8.9, 10.1)	9.4 (8.9, 10.1)
		Absolute number of avoided suicides (95% UI)	−72 (−614, 469)	−140 (−746, 429)	−166 (−802, 506)	−161 (−832, 536)	−171 (−832, 614)	−145 (−899, 609)	−165 (−954, 642)	−203 (−1,018, 671)	−213 (−1,162, 769)	−234 (−1,226, 685)
	Females	No restriction, age-standardized rate (95% CI)	2.7 (2.5, 2.9)	2.7 (2.5, 2.9)	2.7 (2.5, 3.0)	2.7 (2.4, 3.0)	2.7 (2.4, 3.1)	2.7 (2.4, 3.1)	2.7 (2.5, 3.1)	2.7 (2.4, 3.2)	2.7 (2.4, 3.3)	2.8 (2.4, 3.3)
		Means restriction, age-standardized rate (95% CI)	2.7 (2.5, 2.8)	2.7 (2.5, 2.9)	2.7 (2.4, 2.9)	2.7 (2.4, 3.0)	2.7 (2.4, 3.0)	2.7 (2.4, 3.1)	2.7 (2.4, 3.1)	2.7 (2.4, 3.2)	2.7 (2.4, 3.3)	2.7 (2.4, 3.3)
		Absolute number of avoided suicides (95% UI)	−16 (−236, 208)	−29 (−279, 237)	−33 (−360, 278)	−30 (−401, 354)	−29 (−440, 383)	−24 (−517, 427)	−27 (−556, 493)	−31 (−597, 508)	−32 (−629, 527)	−34 (−685, 614)
Non-Latin Caribbean^d^	Males	No restriction, age-standardized rate (95% CI)	14.3 (13.3, 15.3)	14.1 (13.1, 15.3)	13.9 (12.9, 15.1)	13.8 (12.7, 15.1)	13.8 (12.7, 15.1)	13.7 (12.6, 15.1)	13.6 (12.4, 14.9)	13.5 (12.4, 14.9)	13.5 (12.3, 14.9)	13.4 (12.3, 14.9)
		Means restriction, age-standardized rate (95% CI)	12.8 (12.5, 13.4)	11.3 (11.0, 11.8)	10.8 (10.4, 11.3)	10.9 (10.5, 11.5)	10.8 (10.4, 11.5)	11.2 (10.7, 11.9)	10.7 (10.2, 11.5)	10.0 (9.5, 10.7)	9.8 (9.2, 10.5)	9.3 (8.7, 10.0)
		Absolute number of avoided suicides (95% UI)	−55 (−96, −18)	−104 (−148, −61)	−120 (−166, −73)	−110 (−156, −61)	−112 (−160, −62)	−94 (−145, −45)	−108 (−162, −52)	−133 (−188, −83)	−140 (−196, −87)	−156 (−214, −97)
	Females	No restriction, age-standardized rate (95% CI)	4.1 (3.8, 4.3)	4.0 (3.8, 4.3)	4.0 (3.8, 4.4)	4.1 (3.7, 4.4)	4.1 (3.7, 4.4)	4.1 (3.7, 4.5)	4.1 (3.6, 4.5)	4.1 (3.6, 4.5)	4.1 (3.6, 4.5)	4.1 (3.6, 4.6)
		Means restriction, age-standardized rate (95% CI)	3.6 (3.5, 3.7)	3.2 (3.1, 3.3)	3.1 (3.0, 3.2)	3.2 (3.1, 3.3)	3.2 (3.1, 3.3)	3.3 (3.1, 3.5)	3.1 (3.0, 3.3)	2.9 (2.8, 3.1)	2.8 (2.7, 3.0)	2.7 (2.5, 2.9)
		Absolute number of avoided suicides (95% UI)	−16 (−26, −7)	−31 (−42, −20)	−37 (−49, −24)	−33 (−47, −20)	−33 (−47, −18)	−28 (−44, −13)	−34 (−49, −18)	−42 (−59, −25)	−45 (−63, −27)	−51 (−68, −34)
North American^e^	Males	No restriction, age-standardized rate (95% CI)	21.5 (20.8, 22.2)	21.8 (21.1, 22.5)	22.0 (21.2, 22.7)	22.3 (21.5, 23.0)	22.6 (21.8, 23.4)	22.8 (22.0, 23.7)	23.1 (22.2, 24.0)	23.4 (22.4, 24.3)	23.6 (22.6, 24.6)	23.9 (22.8, 25.0)
		Means restriction, age-standardized rate (95% CI)	19.6 (18.5, 20.7)	18.9 (17.8, 20.0)	18.4 (17.4, 19.6)	17.4 (16.3, 18.5)	18.3 (17.1, 19.5)	16.9 (15.8, 18.0)	15.7 (14.7, 16.7)	15.2 (14.2, 16.3)	14.7 (13.8, 15.7)	15.7 (14.7, 16.7)
		Absolute number of avoided suicides (95% UI)	−3432 (−6046, −603)	−5102 (−7445, −2347)	−6538 (−9118, −3945)	−8855 (−11,314, −6161)	−7655 (−10,388, −4962)	−10,690 (−13,335, −7963)	−13,397 (−16,086, −10,653)	−14,665 (−17,278, −11,869)	−16,077 (−18,581, −13,266)	−14,911 (−17,622, −11,965)
	Females	No restriction, age-standardized rate (95% CI)	6.9 (6.7, 7.0)	6.9 (6.7, 7.0)	7.0 (6.8, 7.1)	7.1 (7.0, 7.3)	7.2 (7.1, 7.4)	7.4 (7.2, 7.5)	7.5 (7.3, 7.7)	7.6 (7.4, 7.8)	7.8 (7.5, 7.9)	7.9 (7.7, 8.1)
		Means restriction, age-standardized rate (95% CI)	7.4 (7.1, 7.8)	7.4 (7.0, 7.8)	7.1 (6.8, 7.5)	6.9 (6.6, 7.2)	7.2 (6.8, 7.5)	6.8 (6.5, 7.2)	6.4 (6.1, 6.7)	6.3 (6.0, 6.6)	6.3 (5.9, 6.6)	6.5 (6.1, 6.8)
		Absolute number of avoided suicides (95% UI)	1272 (464, 2018)	1055 (158, 1863)	255 (−615, 1085)	−386 (−1,211, 399)	−147 (−979, 743)	−1065 (−1,883, −207)	−2065 (−2,825, −1226)	−2457 (−3,256, −1604)	−2802 (−3,550, −1991)	−2673 (−3,467, −1742)
Southern Cone^f^	Males	No restriction, age-standardized rate (95% CI)	10.9 (10.6, 11.3)	11.0 (10.6, 11.3)	11.1 (10.6, 11.5)	11.1 (10.6, 11.6)	11.2 (10.7, 11.7	11.3 (10.7, 11.8)	11.3 (10.7, 11.9)	11.4 (10.8, 12.0)	11.4 (10.8, 12.1)	11.5 (10.9, 12.2)
		Means restriction, age-standardized rate (95% CI)	11.0 (10.6, 11.3)	11.0 (10.7, 11.4)	11.1 (10.6, 11.5)	11.1 (10.7, 11.6)	11.2 (10.7, 11.7)	11.3 (10.7, 11.8)	11.3 (10.7, 11.8)	11.3 (10.8, 11.9)	11.4 (10.8, 12.0)	11.5 (10.8, 12.1)
		Absolute number of avoided suicides (95% UI)	88 (−752, 936)	84 (−846, 1029)	46 (−971, 1084)	22 (−1,020, 1.132)	29 (−1,151, 1346)	−6 (−1,313, 1258)	−46 (−1,406, 1255)	−60 (−1,398, 1346)	−71 (−1,583, 1521)	−69 (−1,624, 1600)
	Females	No restriction, age-standardized rate (95% CI)	3.0 (3.0, 3.1)	3.1 (3.0, 3.2)	3.1 (3.0, 3.2)	3.2 (3.0, 3.3)	3.2 (3.0, 3.3)	3.2 (3.1, 3.4)	3.3 (3.1, 3.4)	3.3 (3.1, 3.5)	3.3 (3.1, 3.5)	3.4 (3.1, 3.6)
		Means restriction, age-standardized rate (95% CI)	3.1 (3.0, 3.2)	3.1 (3.0, 3.2)	3.1 (3.0, 3.2)	3.2 (3.0, 3.3)	3.2 (3.1, 3.3)	3.2 (3.1, 3.4)	3.3 (3.1, 3.4)	3.3 (3.1, 3.5)	3.3 (3.1, 3.6)	3.4 (3.2, 3.6)
		Absolute number of avoided suicides (95% UI)	33 (−355, 391)	34 (−379, 436)	22 (−386, 454)	16 (−447, 483)	18 (−463, 538)	9 (−528, 516)	−2 (−576, 566)	−5 (−596, 623)	−8 (−636, 645)	−8 (−718, 684)
Region of the Americas	Males	No restriction, age-standardized rate (95% CI)	13.7 (13.4, 14.0)	13.8 (13.5, 14.1)	13.9 (13.5, 14.2)	14.0 (13.6, 14.3)	14.1 (13.7, 14.4)	14.1 (13.7, 14.5)	14.2 (13.8, 14.6)	14.3 (13.9, 14.7)	14.4 (14.0, 14.8)	14.5 (14.1, 15.0)
		Means restriction, age-standardized rate (95% CI)	13.0 (12.6, 13.5)	12.8 (12.3, 13.2)	12.5 (12.0, 12.9)	12.2 (11.7, 12.6)	12.5 (12.0, 12.9)	12.0 (11.5, 12.4)	11.5 (11.1, 11.9)	11.4 (11.0, 11.7)	11.2 (10.8, 11.6)	11.5 (11.1, 11.9)
		Absolute number of avoided suicides (95% UI)	−3471 (−6,437, −578)	−5262 (−8,023, −2435)	−6778 (−9,395, −3916)	−9104 (−12,029, −5868)	−7909 (−11,026, −4572)	−10,935 (−13,837, −7690)	−13,717 (−16,524, −10,412)	−15,061 (−18,455, −11,779)	−16,501 (−19,761, −13,222)	−15,371 (−18,557, −11,397)
	Females	No restriction, age-standardized rate (95% CI)	4.1 (4.0, 4.2)	4.2 (4.1, 4.2)	4.2 (4.1, 4.3)	4.2 (4.1, 4.3)	4.3 (4.2, 4.4)	4.3 (4.2, 4.5)	4.4 (4.3, 4.5)	4.4 (4.3, 4.6)	4.5 (4.3, 4.6)	4.5 (4.4, 4.7)
		Means restriction, age-standardized rate (95% CI)	4.4 (4.2, 4.5)	4.3 (4.2, 4.5)	4.2 (4.1, 4.4)	4.2 (4.0, 4.3)	4.3 (4.1, 4.4)	4.1 (4.0, 4.3)	4.0 (3.8, 4.1)	4.0 (3.8, 4.1)	4.0 (3.8, 4.2)	4.0 (3.9, 4.2)
		Absolute number of avoided suicides (95% UI)	1274 (286, 2176)	1028 (51, 2013)	208 (−864, 1186)	−433 (−1,544, 661)	−191 (−1,366, 941)	−1109 (−2,290, 12)	−2128 (−3,326, −964)	−2536 (−3,790, −1227)	−2887 (−4,242, −1459)	−2767 (−4,150, −1290)

a A means restriction (firearm or pesticide restriction) implemented in 2020 for those countries where the respective means accounted for 20% or more of all suicides for at least one sex.

b No country within this subregion had firearms or pesticides account for 20% or more of all suicides for at least one sex.

c Pesticide restriction modeled for El Salvador and Nicaragua.

d Pesticide restriction modeled for Guyana, Suriname, and Trinidad and Tobago.

e Firearm restriction modeled for the United States of America.

f Firearm restriction modeled for Uruguay.

Based on the above, lowering the threshold to 20% or more–i.e., the respective means had to account for 20% or more of all suicides for at least one sex for a country to implement a means restriction–did not result in a notable number of additional deaths by suicide avoided. Specifically, it was estimated that by 2030 a total of 69 additional deaths by suicides in the Regional of the Americas would have been avoided. However, the additional avoided deaths were estimated to occur among males only (deaths by suicide were estimated to increase among females), resulting in a 0.2% lower suicide mortality rate among males and no change in the suicide mortality rate among females.

For the country-specific predicted age-standardized suicide mortality rates per 100,000 population and number of avoided deaths by suicide under counterfactual scenario 1 and 2 see Supplementary Table S16.

## Discussion

The findings of the current study demonstrate that firearm or pesticide restriction policies (i.e., means restriction policies that can be implemented on a national scale) have the potential to lower the suicide mortality rate within the Region of the Americas. Both of these forms of means restriction have been implemented elsewhere and have been found to be effective in preventing suicide. For instance, in Sri Lanka a 3-year phased ban of the pesticides dimethoate and fenthion occurred in 2008–10 and paraquat in 2009–11; Knipe and colleagues^26^ found that the overall suicide mortality rate dropped by 21% between 2011 and 2015 (from 18.3 to 14.3 per 100,000 population). In addition to Sri Lanka, success has also been observed in India and China–where suicide rates were reduced, particularly among young females, following bans of highly hazardous pesticides.^28^

With respect to the firearm restriction modeled here,^7^ when the proportion of firearm-involved suicides was below 40%, the restriction did not result in an immediate reduction in the suicide mortality rate, but rather took a few years for a rather minimal reduction to be observed (as was the case for Uruguay). This is in line with the current literature, which suggests that at the population level, means restriction proves to be the most effective when the method is common, accounting for a high proportion of deaths by suicide.^29^^,^^30^ As such, based on our findings, it appears that a firearm restriction would be the most effective in reducing the suicide mortality rate in countries where the proportion of firearm-involved suicides is high (e.g., 40% or more). In the case of the Region of the Americas, this was only the case for males in the United States of America. This also explains why minimal gains were estimated when the threshold for means restriction was lowered to 20% or more.

The most notable reduction observed under the counterfactual scenarios modeled was for the Non-Latin Caribbean subregion. The Non-Latin Caribbean subregion would have reduced their suicide mortality rate by as much as 31% among males and by 34% among females if they would have implemented a pesticide restriction in three countries (Guyana, Suriname, and Trinidad and Tobago) in 2020. One concern about pesticide restrictions specifically is that it would lead to loss of agricultural productivity. However, Stuart and colleagues^31^ recently analyzed production data from eleven countries and found that it consistently failed to show any negative effects of banning a highly hazardous pesticide, paraquat, on agricultural productivity. In this review, the authors provide a wide range of alternative approaches to weed management and foliage removal, many of which do not rely on chemical herbicides, as well as economic considerations.^31^ Another argument against means restriction policies in general is that individuals will simply use another method of suicide–often referred to as means substitution. Given the potential for means substitution to influence the overall reduction in suicide rates following the implementation of a restriction policy, we took into consideration the effect of each type of restriction on other means of suicide. In fact, this was a criterion for inclusion of studies reporting the effect of such restriction policies (i.e., the study had to provide the impact of the respective restriction on the mean-specific and non-mean-specific suicide mortality rate). Taking the possible means substitution phenomena into consideration provided a more nuanced analysis, and decreased the likelihood of overestimating the impact of the means restrictions. The findings of the current study suggest that although individual studies have shown evidence of means substitution (see ^10^^,^^26^ for example), the gains in lives saved outweigh the suicide losses by other means. This finding is consistent with the literature. For instance, in the review by Lim and colleagues,^32^ it was found that means restriction of poison was associated with decreased method-specific suicide rates without an equivalent shift toward the use of other methods. Specifically, based on pre- and post-data for 29 interventions, the median (interquartile range; IQR) change in method-specific suicide rates was −1.18 (−2.03 to −0.46) per 100,000 population post-implementation, whereas the median (IQR) change in other methods of suicide was −0.09 (−2.22 to 1.65) per 100,000 population.

There are some limitations that must be acknowledged. First, any modeling study is only as good as the underlying data. Statistics on suicide may be subject to bias, and classification and registration may differ across countries. Problems with both reliability and comparability of national suicide statistics have been noted.^33^^,^^34^ While the WHO Global Health Estimates try to remove some of the problems, e.g., reallocating non-informative codes, such as “not otherwise specified”, some bias may remain. Second, the 2019 sex-specific proportion of firearm- and pesticide-involved suicides was applied to the forecasted rates of 2020–2030, and therefore, the proportions were assumed to have not changed over this time. Although this is likely not the case, as there could be changes in trends, societal behavior or legislation that occur during the forecasted period that may impact the means-specific proportions, it was the best we could do with the data we had available. It is reasonable to deduce that if the proportion of firearm- or pesticide-involved suicides were to increase, the implementation of a restriction on the respective means would likely further decrease the overall suicide mortality rate–the reverse is also likely true. Third, the rate ratio of the suicide mortality rate pre- and annual post-intervention obtained from the existing literature were not sex-specific, nor age-specific. Evidence does suggest that females are more responsive to means restriction measures than males.^16^ Therefore, assuming consistent effects across sexes may overlook potential gender-based differences in the impact of the respective bans. It is also possible that there are differences in the proportion firearm- and pesticide-involved suicides, as well as differences in the impact of the respective bans across the different age-groups that we were not able to account for. Accordingly, we would advocate for further research that provides these disaggregated data, which would ultimately allow us to refine our estimates. Fourth, given the ecological study design, there is a possibility that relationships observed and modeled at the country level may not reflect relationships at the individual level. Fifth, the effect estimates were drawn from single studies. Although they are supported by the current literature (for example, see^35^), they may reflect a sociocultural context that is not identical to that of the countries in the Americas for which the means restrictions were modeled. However, having said that, a study on the impact of a law banning the purchase, sale, transfer, or possession of handguns by civilians in Washington D.C., found that the enactment was associated with an abrupt 23% reduction in firearm-involved suicides.^36^ These findings suggest that, in the US context, a national firearm-restriction would be effective in reducing the suicide mortality rate. Unfortunately, there is no study evaluating the impact of a pesticide ban on the suicide rate for any country in the Region of the Americas. Lastly, it is important to note that the impact of each form of means restriction was modeled on forecasted suicide mortality rates. Despite our best effort to ensure that the forecasted rates were reasonable and reflective of the current country-specific trend, by testing multiple models, it is possible that they differ from reality, which will only be known when the time comes.

Although not a limitation of the current study per se, as it was decided a priori that firearm or pesticide restrictions would not be modeled for countries with a suicide mortality rate less than three per 100,000 population in 2019, the effect estimates used in the modeling were not obtained from a low-incidence setting, and thus, may not be generalizable to such settings. This does, however, highlight that future research should explore this if the situation were to arise (that is if a country with a low suicide mortality rate were to implement a means restriction policy). Further, as the current study was limited to the Americas, we would caution against extrapolating the results to other regions, as there may be important socio-cultural factors influencing the incidence of firearm- and pesticide-involved suicides to consider elsewhere. Instead, we would encourage researchers to consider conducting similar analyses for other regions.

We would be amiss if we did not discuss the challenges associated with implementation of means restriction. The implementation of means restriction could be viewed as intrusive by members of the public, as the benefits for the majority of the people would be minimal, and thus, met with significant reluctance to accept. Such opposition could be overcome with appropriate media coverage and endorsement by community leaders.^6^ Related to this is the necessity of understanding the cultural beliefs surrounding the targeted means. Restricting a means which has significant cultural meaning may be met with public resistance and in such situations, education efforts should be enhanced. Second, the success of a restriction in reducing the suicide mortality rate is dependent on it being maintained and enforced. This will require collaboration and cooperation with local authorities. Lastly, as specified in the WHO LIVE LIFE,^5^ monitoring and evaluation should be part of all interventions. However, the effectiveness of a means restriction policy can be assessed only in the long-term.

## Conclusion

It is apparent that a ban of highly hazardous pesticides in countries where pesticide-involved suicides account for a large proportion of overall suicides would be an effective suicide prevention policy within the Region of the Americas; such a policy has also been found to be cost-effective.^37^ Although a firearm ban could prove to be effective in reducing the suicide mortality rate in a given country, the effects appear to be more nuanced–for example, if there is a notable difference between the sexes in the proportion of firearm-involved suicides, the suicide mortality rate for the sex with the lower proportion may not be reduced. This highlights the fact that comprehensive suicide prevention strategies must be guided by a situation analysis of the local epidemiology of suicide. In conclusion, multisectoral collaboration for restricting access to means of suicide as a universal evidence-based intervention for suicide prevention could aid the Region of the Americas in achieving the WHO target of a one third reduction in the suicide mortality rate by 2030.^1^

## Contributors

SL, JR, AJMH and ROS were responsible for the study conceptualization. SL and KVK were responsible for the data curation, and had directly accessed and verified the underlying data. SL and JR developed the methodology. KVK, HJ and KDS performed the formal analyses. SL prepared the visualizations, and was responsible for writing the manuscript. JR, AJMH, and ROS reviewed and edited the manuscript. All authors approved the final version of the manuscript, and were responsible for the decision to submit the manuscript.

## Data sharing statement

The data that support the findings of this study are openly available in the WHO Global Health Estimates database (https://www.who.int).

## Declaration of interests

Shannon Lange declared receiving payment from the Pan American Health Organization as a consultant for work related to the current manuscript.
