# Supplementary material for: Forecasting the impact of means restriction on the suicide mortality rate in the Region of the Americas: an ecological modeling study
Source: Lancet Reg Health Am. 2024 Jul 16;36:100831. doi: 10.1016/j.lana.2024.100831 (PMC11372382; doi:10.1016/j.lana.2024.100831)
Supplement: Supplementary Figures and Tables [file mmc1.pdf]

Forecasting the Impact of Means Restriction on the Suicide Mortality Rate in the Region of the Americas: An Ecological Modeling Study

Supplementary Material

Table of Contents

Supplementary Table 1. STROBE checklist 3

Supplementary Table 2. Joinpoint analysis of age-standardized suicide mortality rates (per 100,000 people) over the past 20 years in the Region of the Americas and its sub-regions 4

Supplementary Figure 1. Age-standardized suicide mortality rate among males, and the trend over time in the Region of the Americas and its sub-regions, 2000-2019 5

Supplementary Figure 2. Age-standardized suicide mortality rate among females, and the trend over time in the Region of the Americas and its sub-regions, 2000-2019 6

Supplementary Table 3. Joinpoint analysis of age-standardized suicide mortality rates (per 100,000 people) over the past 20 years in the Andean Area sub-region, by country 7

Supplementary Figure 3. Age-standardized suicide mortality rate among males, and the trend over time in the Andean Area sub-region and its countries, 2000-2019 8

Supplementary Figure 4. Age-standardized suicide mortality rate among females, and the trend over time in the Andean Area sub-region and its countries, 2000-2019 9

Supplementary Table 4. Joinpoint analysis of age-standardized suicide mortality rates (per 100,000 people) over the past 20 years in the Central America, Mexico and Latin Caribbean sub-region, by country 10

Supplementary Figure 5. Age-standardized suicide mortality rate among males, and the trend over time in the Central America, Mexico and Latin Caribbean sub-region and its countries, 2000-2019 11

Supplementary Figure 6. Age-standardized suicide mortality rate among females, and the trend over time in the Central America, Mexico and Latin Caribbean sub-region and its countries, 2000-2019 12

Supplementary Table 5. Joinpoint analysis of age-standardized suicide mortality rates (per 100,000 people) over the past 20 years in the Non-Latin Caribbean sub-region, by country 13

Supplementary Figure 7. Age-standardized suicide mortality rate among males, and the trend over time in the Non-Latin Caribbean sub-region and its countries, 2000-2019 14

Supplementary Figure 8. Age-standardized suicide mortality rate among females, and the trend over time in the Non-Latin Caribbean sub-region and its countries, 2000-2019 15

Supplementary Table 6. Joinpoint analysis of age-standardized suicide mortality rates (per 100,000 people) over the past 20 years in the North America sub-region, by country 16

Supplementary Figure 9. Age-standardized suicide mortality rate among males, and the trend over time in the North America sub-region and its countries, 2000-2019 17

Supplementary Figure 10. Age-standardized suicide mortality rate among females, and the trend over time in the North America sub-region and its countries, 2000-2019 18

Supplementary Table 7. Joinpoint analysis of age-standardized suicide mortality rates (per 100,000 people) over the past 20 years in the Southern Cone sub-region, by country 19

Supplementary Figure 11. Age-standardized suicide mortality rate among males, and the trend over time in the Southern Cone sub-region and its countries, 2000-2019 20

Supplementary Figure 12. Age-standardized suicide mortality rate among females, and the trend over time in the Southern Cone sub-region and its countries, 2000-2019 21

Supplementary Table 8. Observed and Forecasted Sex-specific Age-standardized Suicide Mortality Rates and 95% Confidence Intervals Among Males, 2000-2030 22

Supplementary Table 9. Observed and Forecasted Sex-specific Age-standardized Suicide Mortality Rates and 95% Confidence Intervals Among Females, 2000-2030 24

Supplementary Table 10. Model fit statistics for the forecasting models 26

Supplementary Table 11. Proportion of firearm- and pesticide-involved suicides by country and sex in the Region of Americas 28

Supplementary Table 12. Search strategy employed in PubMed, Embase, and PsycInfo (OVID) 29

Supplementary Table 13. PICOS criteria for study selection 30

Supplementary Figure 13. PRISMA flow diagram of study selection 31

Supplementary Table 14. Effect estimates for a firearm ban 32

Supplementary Table 15. Effect estimates for a pesticide ban 33

Supplementary Table 16. Predicted age-standardized suicide mortality rate per 100,000 population and number of avoided deaths by suicide in seven countries in the Region of the Americas under a counterfactual scenario of a pesticide or firearm restriction implemented in 2020 for the years 2021-2030 34

Supplementary Table 1. STROBE checklist

	Item No	Recommendation	Page No
**Title and abstract**	1	(*a*) Indicate the study's design with a commonly used term in the title or the abstract	1,2
		(*b*) Provide in the abstract an informative and balanced summary of what was done and what was found	2-3
**Introduction**			
Background/rationale	2	Explain the scientific background and rationale for the investigation being reported	4-5
Objectives	3	State specific objectives, including any prespecified hypotheses	5
**Methods**			
Study design	4	Present key elements of study design early in the paper	5
Setting	5	Describe the setting, locations, and relevant dates, including periods of recruitment, exposure, follow-up, and data collection	6
Participants	6	Give the eligibility criteria, and the sources and methods of selection of participants	6
Variables	7	Clearly define all outcomes, exposures, predictors, potential confounders, and effect modifiers. Give diagnostic criteria, if applicable	6
Data sources/ measurement	8	For each variable of interest, give sources of data and details of methods of assessment (measurement). Describe comparability of assessment methods if there is more than one group	6-11
Bias	9	Describe any efforts to address potential sources of bias	9
Study size	10	Explain how the study size was arrived at	n/a
Quantitative variables	11	Explain how quantitative variables were handled in the analyses. If applicable, describe which groupings were chosen and why	6
Statistical methods	12	(*a*) Describe all statistical methods, including those used to control for confounding	7-11
		(*b*) Describe any methods used to examine subgroups and interactions	n/a
		(*c*) Explain how missing data were addressed	7-8
		(*d*) If applicable, describe analytical methods taking account of sampling strategy	n/a
		(*e*) Describe any sensitivity analyses	n/a
**Results**			
Participants	13	(a) Report numbers of individuals at each stage of study—eg numbers potentially eligible, examined for eligibility, confirmed eligible, included in the study, completing follow-up, and analysed	n/a
		(b) Give reasons for non-participation at each stage	n/a
		(c) Consider use of a flow diagram	n/a
Descriptive data	14	(a) Give characteristics of study participants (eg demographic, clinical, social) and information on exposures and potential confounders	n/a
		(b) Indicate number of participants with missing data for each variable of interest	n/a
Outcome data	15	Report numbers of outcome events or summary measures	Table 1 & 2
Main results	16	(*a*) Give unadjusted estimates and, if applicable, confounder-adjusted estimates and their precision (eg, 95% confidence interval). Make clear which confounders were adjusted for and why they were included	12-14
		(*b*) Report category boundaries when continuous variables were categorized	n/a
		(*c*) If relevant, consider translating estimates of relative risk into absolute risk for a meaningful time period	n/a
Other analyses	17	Report other analyses done—eg analyses of subgroups and interactions, and sensitivity analyses	n/a
**Discussion**			
Key results	18	Summarise key results with reference to study objectives	14-15
Limitations	19	Discuss limitations of the study, taking into account sources of potential bias or imprecision. Discuss both direction and magnitude of any potential bias	17-18
Interpretation	20	Give a cautious overall interpretation of results considering objectives, limitations, multiplicity of analyses, results from similar studies, and other relevant evidence	18-19
Generalisability	21	Discuss the generalisability (external validity) of the study results	19-20
**Other information**			
Funding	22	Give the source of funding and the role of the funders for the present study and, if applicable, for the original study on which the present article is based	21

Supplementary Table 2. Joinpoint analysis of age-standardized suicide mortality rates (per 100,000 people) over the past 20 years in the Region of the Americas and its sub-regions

**Location**	**Sex**	**Mortality rate[a]**		**Total study period (2000-2019)**		**Period 1**			**Period 2**			**Period 3**			**Period 4**		
		**2000**	**2019**	**AAPC (%)**	**95% CI**	**Years**	**APC (%)**	**95% CI**	**Years**	**APC (%)**	**95% CI**	**Years**	**APC (%)**	**95% CI**	**Years**	**APC (%)**	**95% CI**
Andean Area																	
	M	8.50	6.05	**-1.79**	**-2.54, -1.03**	**2000-11**	**-1.37**	**-1.69, -1.06**	**2011-14**	**-4.03**	**-7.35, -0.60**	2014-17	2.18	-1.36, 5.84	**2017-19**	**-6.37**	**-9.64, -2.98**
	F	2.69	1.85	**-2.09**	**-2.32, -1.86**	**2000-19**	**-2.09**	**-2.32, -1.86**	-	-	-	-	-	-	-	-	-
Central America, Mexico and Latin Caribbean																	
	M	10.33	9.75	-0.23	-1.15, 0.69	2000-04	0.65	-0.75, 2.08	2004-07	-1.95	-7.49, 3.94	**2007-16**	**0.69**	**0.26, 1.12**	**2016-19**	**-2.41**	**-4.76, -0.01**
	F	2.73	2.58	-0.35	-1.73, 1.04	**2000-06**	**-2.42**	**-3.73, -1.10**	2006-09	3.03	-6.00, 12.93	2009-19	-0.09	-0.70, 0.51	-	-	-
Non-Latin Caribbean																	
	M	17.48	15.1	**-1.04**	**-1.45, -0.62**	**2000-06**	**-2.98**	**-4.14, -1.80**	2006-19	-0.13	-0.50, 0.24	-	-	-	-	-	-
	F	4.35	4.05	-0.48	-0.98, 0.02	**2000-06**	**-2.10**	**-3.52, -0.66**	2006-19	0.27	-0.15, 0.70	-	-	-	-	-	-
North America																	
	M	16.45	21.65	**1.36**	**0.58, 2.14**	**2000-14**	**0.87**	**0.71, 1.03**	**2014-17**	**5.12**	**0.56, 9.90**	2017-19	-0.68	-5.07, 3.92	-	-	-
	F	4.05	6.61	**2.67**	**2.33, 3.01**	**2000-04**	**3.50**	**2.43, 4.59**	**2004-09**	**1.60**	**0.57, 2.65**	**2009-19**	**2.87**	**2.63, 3.10**	-	-	-
Southern Cone																	
	M	9.99	11.26	**0.72**	**0.27, 1.16**	2000-13	-0.08	-0.47, 0.30	**2013-19**	**2.46**	**1.15, 3.78**	-	-	-	-	-	-
	F	2.23	2.93	**1.10**	**0.83, 1.37**	**2000-19**	**1.10**	**0.83, 1.37**	-	-	-	-	-	-	-	-	-
Region of the Americas																	
	M	12.63	14.16	0.49	-0.16, 1.15	2000-06	-0.22	-0.77, 0.34	**2006-14**	**0.50**	**0.06, 0.94**	2014-17	2.78	-0.98, 6.69	2017-19	-0.81	-4.46, 2.98
	F	3.23	4.08	**1.25**	**0.73, 1.78**	2000-02	3.39	-0.80, 7.76	2002-06	0.02	-1.68, 1.74	**2006-19**	**1.31**	**1.16, 1.46**	-	-	-

APC: Average annual percent change; APC: Annual percent change; CI: Confidence interval; F: Females; M: Males

[a]per 100,000 population

Note. Bolded text indicates statistical significance (p<0.05).

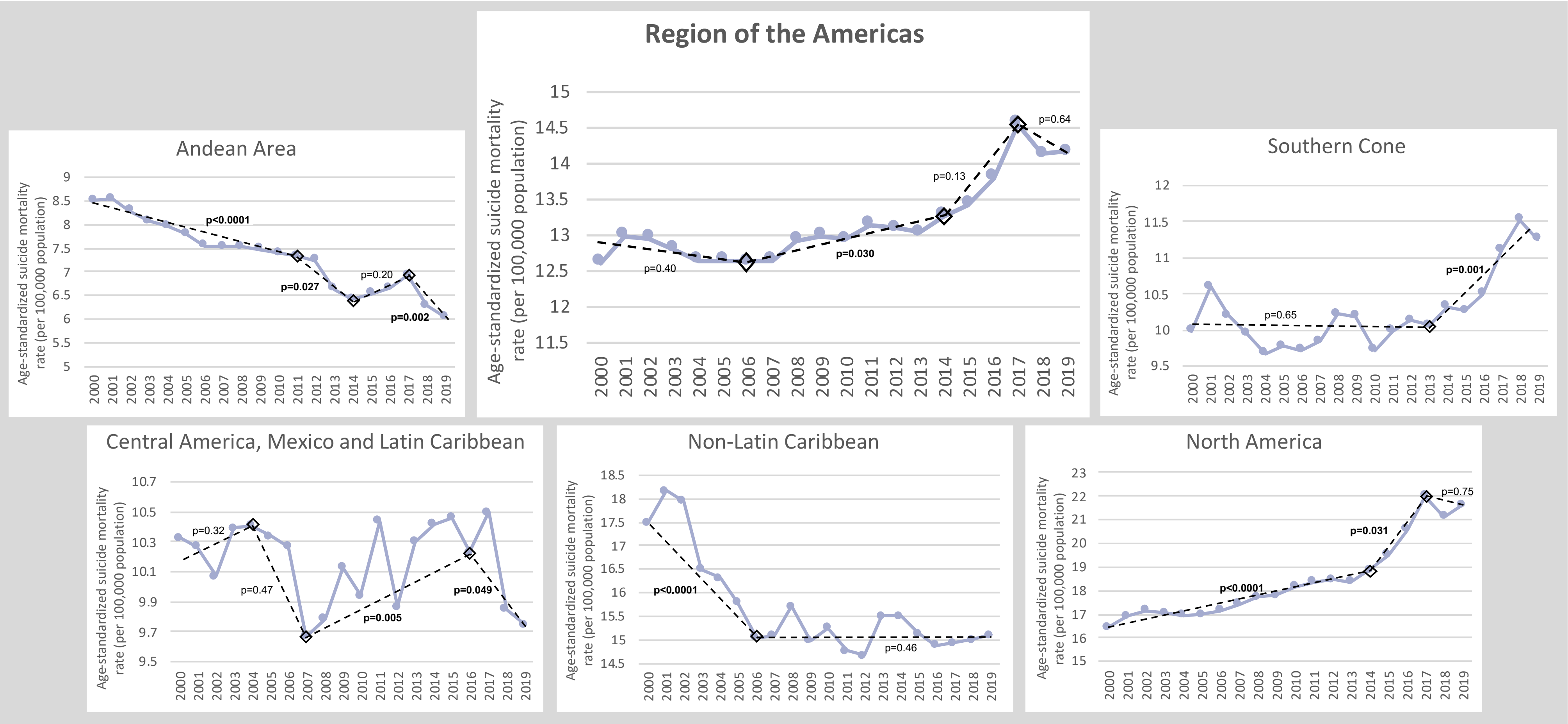

Supplementary Figure 1. Age-standardized suicide mortality rate among males, and the trend over time in the Region of the Americas and its sub-regions, 2000-2019
Note. Diamonds indicate identified points of inflection. Statistically significant linear segments at $p<0.05$ are bolded.

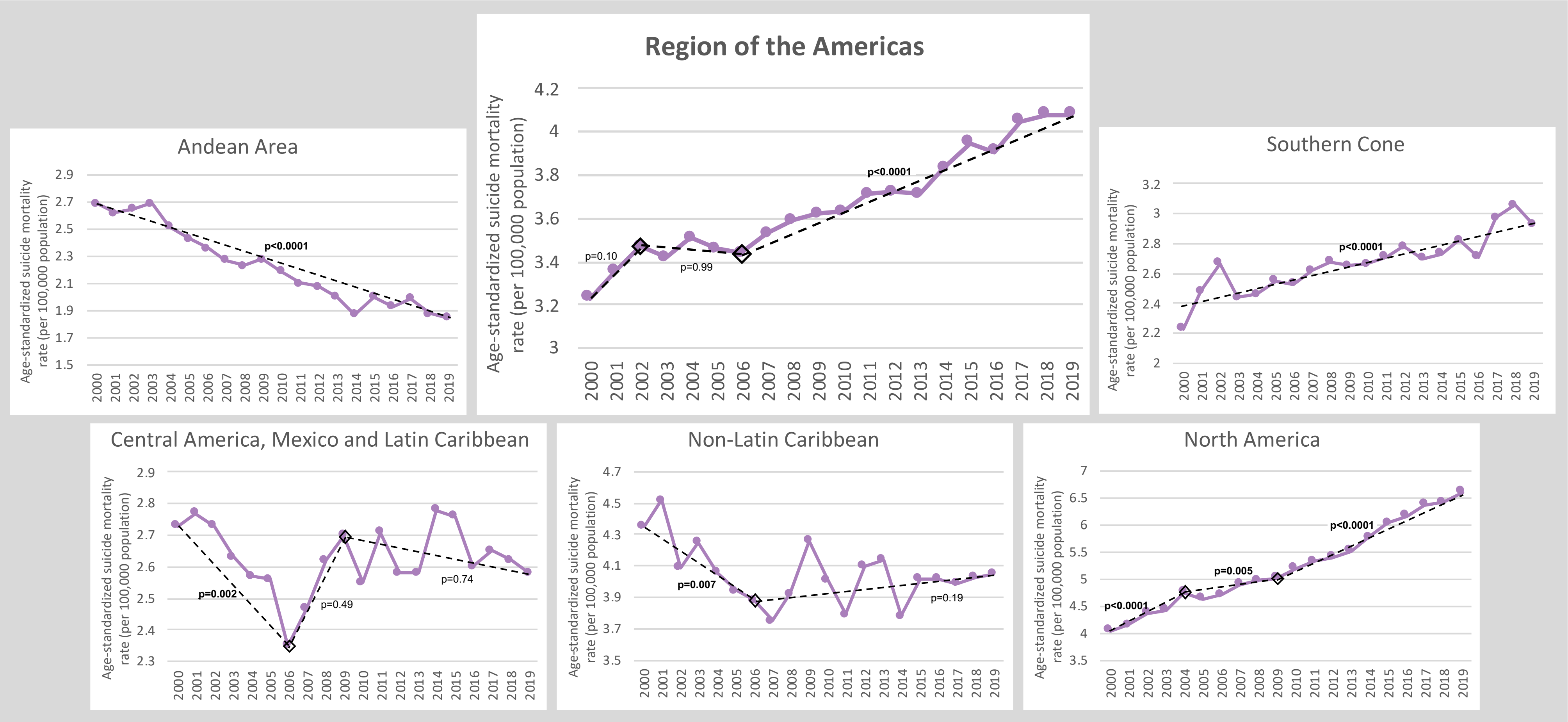

Supplementary Figure 2. Age-standardized suicide mortality rate among females, and the trend over time in the Region of the Americas and its sub-regions, 2000-2019
Note. Diamonds indicate identified points of inflection. Statistically significant linear segments at p<0.05 are bolded.

Supplementary Table 3. Joinpoint analysis of age-standardized suicide mortality rates (per 100,000 people) over the past 20 years in the Andean Area sub-region, by country

**Country**	**Sex**	**Mortality rate**[a]		**Total study period (2000-2019)**		**Period 1**			**Period 2**			**Period 3**			**Period 4**		
		**2000**	**2019**	**AAPC (%)**	**95% CI**	**Years**	**APC (%)**	**95% CI**	**Years**	**APC (%)**	**95% CI**	**Years**	**APC (%)**	**95% CI**	**Years**	**APC (%)**	**95% CI**
Bolivia																	
	M	11.76	9.60	**-1.10**	**-1.51, -0.69**	**2000-06**	**-3.21**	**-3.61, -2.81**	**2006-13**	**-0.60**	**-1.01, -0.19**	**2013-16**	**3.41**	**0.85, 6.04**	**2016-19**	**-2.41**	**-3.61, -1.19**
	F	5.15	4.19	**-1.00**	**-1.41 -0.58**	**2000-05**	**-1.44**	**-2.35, -0.52**	2005-16	-0.21	-0.51, 0.10	**2016-19**	**-3.12**	**-5.28, -0.91**	-	-	-
Colombia																	
	M	8.38	5.96	**-1.80**	**-3.10, -0.49**	**2000-13**	**-2.44**	**-2.87, -2.01**	2013-17	4.73	-0.02, 9.70	**2017-19**	**-9.93**	**-18.59, -0.36**	-	-	-
	F	2.57	1.71	**-2.10**	**-2.81, -1.38**	**2000-11**	**-5.26**	**-6.08, -4.43**	**2011-19**	**2.41**	**0.94, 3.91**	-	-	-	-	-	-
Ecuador																	
	M	9.64	11.88	**1.26**	**0.51, 2.01**	**2000-07**	**7.64**	**5.78, 9.53**	**2007-19**	**-2.29**	**-3.05, -1.53**	-	-	-	-	-	-
	F	4.16	3.60	-0.72	-1.44, 0.01	**2000-05**	**6.46**	**4.72, 8.22**	2005-10	-1.09	-3.35, 1.21	**2010-19**	**-4.30**	**-4.95, -3.64**	-	-	-
Peru																	
	M	4.56	4.10	**-0.50**	**-1.67, 0.70**	**2000-07**	**-1.60**	**-2.42, -0.78**	**2007-10**	**10.26**	**2.54, 18.57**	**2010-16**	**-4.93**	**-6.27, -3.57**	2016-19	0.97	-2.38, 4.43
	F	2.40	1.38	**-2.76**	**-3.92, -1.58**	**2000-07**	**-5.19**	**-5.87, -4.50**	2007-10	7.31	-0.60, 15.85	**2010-15**	**-5.98**	**-7.69, -4.24**	2015-19	-1.54	-3.38, 0.33
Venezuela																	
	M	11.31	3.65	**-6.03**	**-7.77, -4.25**	**2000-08**	**-7.77**	**-8.63, -6.89**	2008-11	1.47	-7.47, 11.26	**2011-14**	**-15.29**	**-22.75, -7.11**	2014-19	-1.59	-3.50, 0.35
	F	1.65	0.69	**-4.33**	**-6.00, -2.63**	2000-02	5.05	-10.54, 23.37	**2002-14**	**-7.20**	**-8.02, -6.36**	2014-19	-0.87	-3.90, 2.26	-	-	-
Andean Area																	
	M	8.50	6.05	**-1.79**	**-2.54, -1.03**	**2000-11**	**-1.37**	**-1.69, -1.06**	**2011-14**	**-4.03**	**-7.35, -0.60**	2014-17	2.18	-1.36, 5.84	**2017-19**	**-6.37**	**-9.64, -2.98**
	F	2.69	1.85	**-2.09**	**-2.32, -1.86**	**2000-19**	**-2.09**	**-2.32, -1.86**	-	-	-	-	-	-	-	-	-

APC: Average annual percent change; APC: Annual percent change; CI: Confidence interval; F: Females; M: Males

[a]per 100,000 population

Note. Bolded text indicates statistical significance (p<0.05).

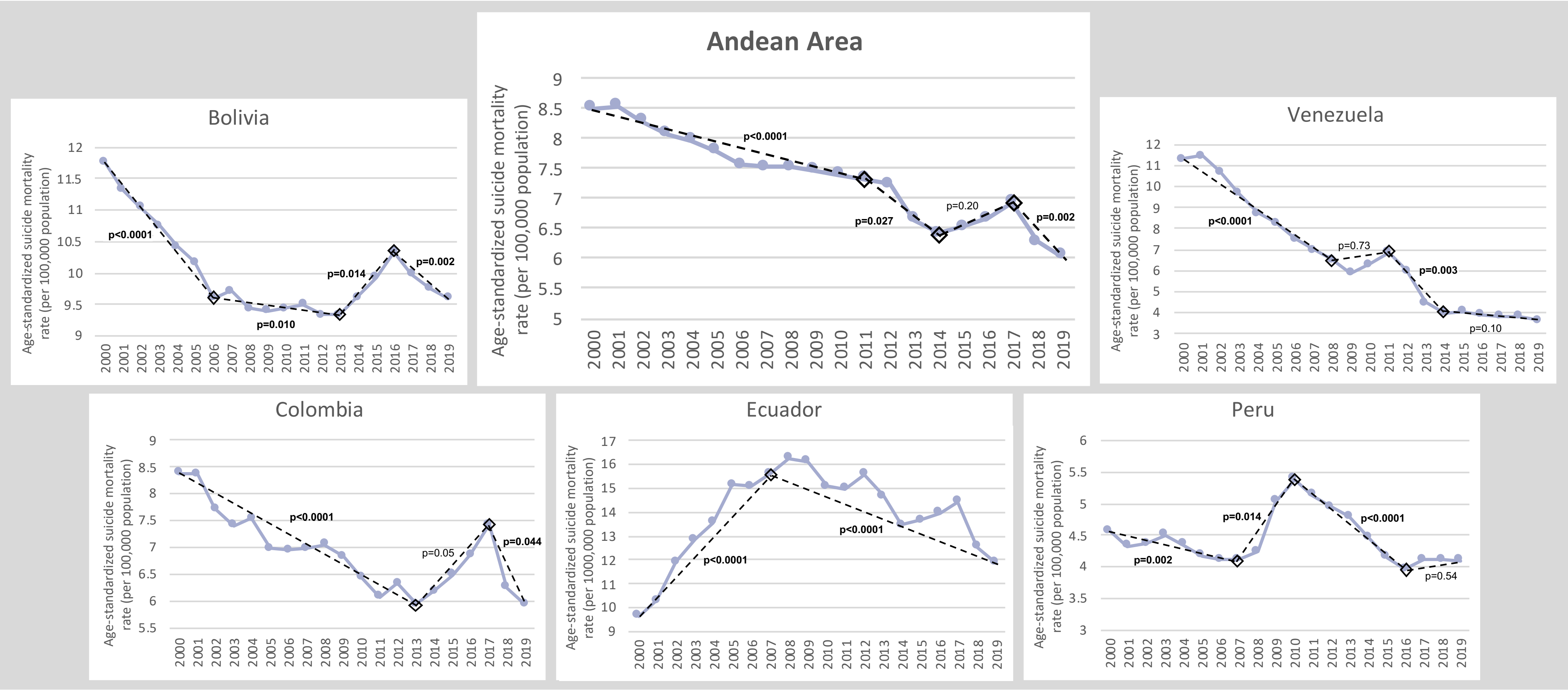

Supplementary Figure 3. Age-standardized suicide mortality rate among males, and the trend over time in the Andean Area sub-region and its countries, 2000-2019
Note. Diamonds indicate identified points of inflection. Statistically significant linear segments at p<0.05 are bolded.

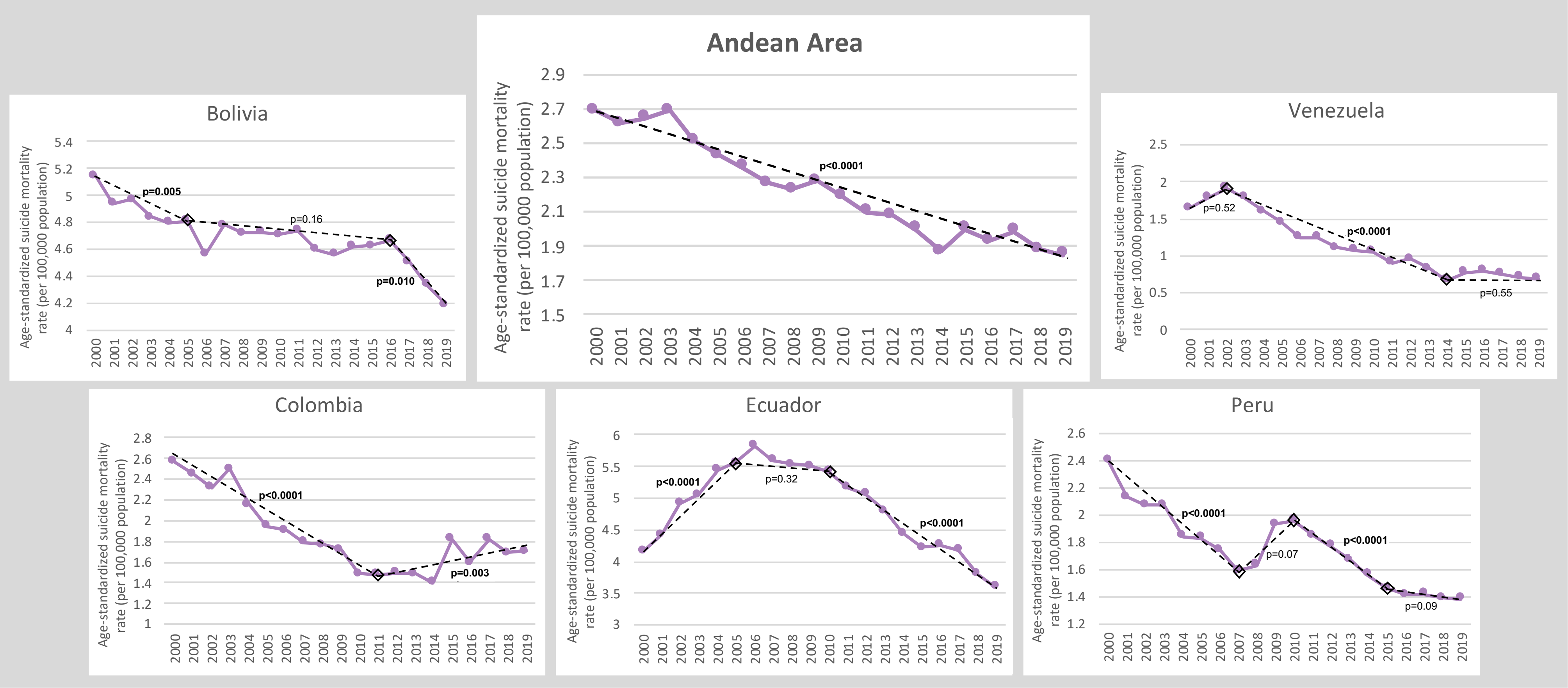

Supplementary Figure 4. Age-standardized suicide mortality rate among females, and the trend over time in the Andean Area sub-region and its countries, 2000-2019
Note. Diamonds indicate identified points of inflection. Statistically significant linear segments at p<0.05 are bolded.

Supplementary Table 4. Joinpoint analysis of age-standardized suicide mortality rates (per 100,000 people) over the past 20 years in the Central America, Mexico and Latin Caribbean sub-region, by country

Country	Sex	Mortality rate[a]		Total study period (2000-2019)		Period 1			Period 2			Period 3			Period 4		
		2000	2019	AAPC (%)	95% CI	Years	APC (%)	95% CI	Years	APC (%)	95% CI	Years	APC (%)	95% CI	Years	APC (%)	95% CI
Costa Rica																	
	M	12.28	13.34	-0.66	-1.67, 0.37	2000-19	-0.66	-1.67, 0.37	-	-	-	-	-	-	-	-	-
	F	1.55	1.93	0.51	-0.76, 1.79	2000-19	0.51	-0.76, 1.79	-	-	-	-	-	-	-	-	-
Cuba																	
	M	22.7	16.67	-1.68	-3.36, 0.03	2000-02	-8.01	-16.65, 1.53	**2002-07**	**-2.94**	**-5.38, -0.44**	2007-10	3.48	-6.07, 14.00	**2010-19**	**-1.20**	**-1.87, -0.53**
	F	8.93	4.08	**-3.99**	**-5.00, -2.97**	**2000-06**	**-10.13**	**-12.84, -7.34**	**2006-19**	**-1.01**	**-1.90, -0.12**	-	-	-	-	-	-
Dominican Republic																	
	M	8.28	8.52	0.41	-0.69, 1.51	**2000-07**	**2.77**	**1.87, 3.68**	2007-11	-1.38	-5.04, 2.42	**2011-15**	**6.77**	**2.81, 10.89**	**2015-19**	**-7.71**	**-9.78, -5.58**
	F	1.57	1.85	0.97	-0.05, 2.01	**2000-04**	**6.69**	**4.18, 9.26**	**2004-11**	**-1.21**	**-2.38, -0.03**	**2011-15**	**7.3**	**3.15, 11.61**	**2015-19**	**-6.57**	**-8.76, -4.31**
El Salvador																	
	M	10.63	11.14	0.05	-1.07, 1.18	**2000-07**	**5.44**	**2.62, 8.34**	**2007-19**	**-2.96**	**-4.05, -1.87**	-	-	-	-	-	-
	F	3.39	2.08	**-2.8**	**-3.76, -1.83**	**2000-06**	**-6.60**	**-9.27, -3.84**	**2006-19**	**-1.00**	**-1.81, -0.17**	-	-	-	-	-	-
Guatemala																	
	M	24.03	10.31	**-4.55**	**-5.50, -3.60**	2000-05	-0.16	-2.72, 2.46	**2005-13**	**-10.04**	**-11.37, -8.69**	2013-19	-0.51	-2.40, 1.42	-	-	-
	F	4.11	2.53	**-2.58**	**-3.21, -1.95**	**2000-04**	**2.89**	**0.57, 5.27**	**2004-12**	**-5.32**	**-6.23, -4.42**	**2012-19**	**-2.45**	**-3.38, -1.51**	-	-	-
Haiti																	
	M	14.71	14.87	0.12	-0.25, 0.49	**2000-04**	**-1.87**	**-2.98, -0.74**	**2004-09**	**2.86**	**1.70, 4.04**	**2009-19**	**-0.44**	**-0.70, -0.17**	-	-	-
	F	11.1	7.98	**-1.74**	**-2.22, -1.26**	**2000-04**	**-1.94**	**-3.40, -0.46**	2004-09	0.97	-0.54, 2.49	**2009-19**	**-2.99**	**-3.34, -2.63**	-	-	-
Honduras																	
	M	5.07	4.39	-0.88	-4.47, 2.86	**2000-10**	**-1.60**	**-2.50, -0.69**	**2010-14**	**16.67**	**8.39, 25.58**	**2014-17**	**-19.59**	**-33.98, -2.07**	2017-19	1.58	-19.30, 27.86
	F	1.14	0.98	**-0.75**	**-1.24, -0.25**	**2000-06**	**0.67**	**0.10, 1.24**	**2006-11**	**-3.32**	**-4.39, -2.24**	2011-15	1.27	-0.61, 3.18	**2015-19**	**-1.59**	**-2.69, -0.47**
Mexico																	
	M	6.84	8.68	0.94	-0.13, 2.01	**2000-07**	**0.99**	**0.15, 1.82**	**2007-17**	**2.62**	**2.07, 3.17**	**2017-19**	**-7.22**	**-16.47, 3.05**	-	-	-
	F	1.12	2.18	**3.11**	**1.92, 4.32**	**2000-15**	**4.10**	**3.44, 4.76**	2015-19	-0.50	-5.84, 5.15	-	-	-	-	-	-
Nicaragua																	
	M	9.34	7.79	**-1.51**	**-2.05, -0.96**	**2000-19**	**-1.51**	**-2.05, -0.96**	-	-	-	-	-	-	-	-	-
	F	3.57	1.91	-3.36	-8.20, 1.73	**2000-06**	**-8.51**	**-12.43, -4.41**	2006-09	9.23	-22.64, 54.25	**2009-19**	**-3.74**	**-5.58, -1.87**	-	-	-
Panama																	
	M	10.38	4.80	**-4.20**	**-5.21, -3.19**	2000-06	-0.82	-3.85, 2.29	**2006-19**	**-5.72**	**-6.55, -4.89**	-	-	-	-	-	-
	F	1.48	0.98	**-3.62**	**-539, -1.83**	**2000-19**	**-3.62**	**-539, -1.83**	-	-	-	-	-	-	-	-	-
Central America, Mexico and Latin Caribbean																	
	M	10.33	9.75	-0.23	-1.15, 0.69	2000-04	0.65	-0.75, 2.08	2004-07	-1.95	-7.49, 3.94	**2007-16**	**0.69**	**0.26, 1.12**	**2016-19**	**-2.41**	**-4.76, -0.01**
	F	2.73	2.58	-0.35	-1.73, 1.04	**2000-06**	**-2.42**	**-3.73, -1.10**	2006-09	3.03	-6.00, 12.93	2009-19	-0.09	-0.70, 0.51	-	-	-

APC: Average annual percent change; APC: Annual percent change; CI: Confidence interval; F: Females; M: Males

[a]per 100,000 population

Note. Bolded text indicates statistical significance ($p<0.05$).

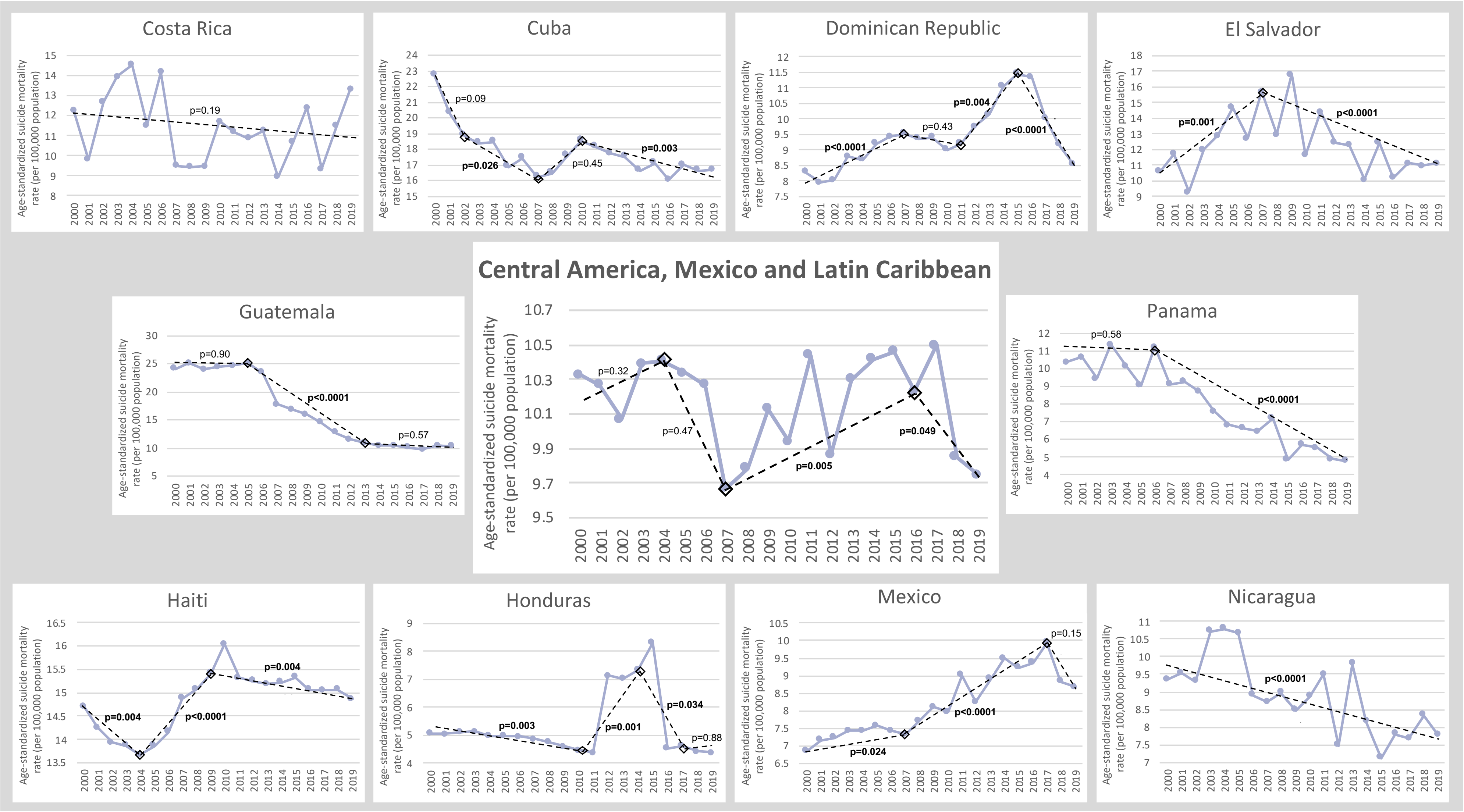

Supplementary Figure 5. Age-standardized suicide mortality rate among males, and the trend over time in the Central America, Mexico and Latin Caribbean sub-region and its countries, 2000-2019

Note. Diamonds indicate identified points of inflection. Statistically significant linear segments at $p<0.05$ are bolded.

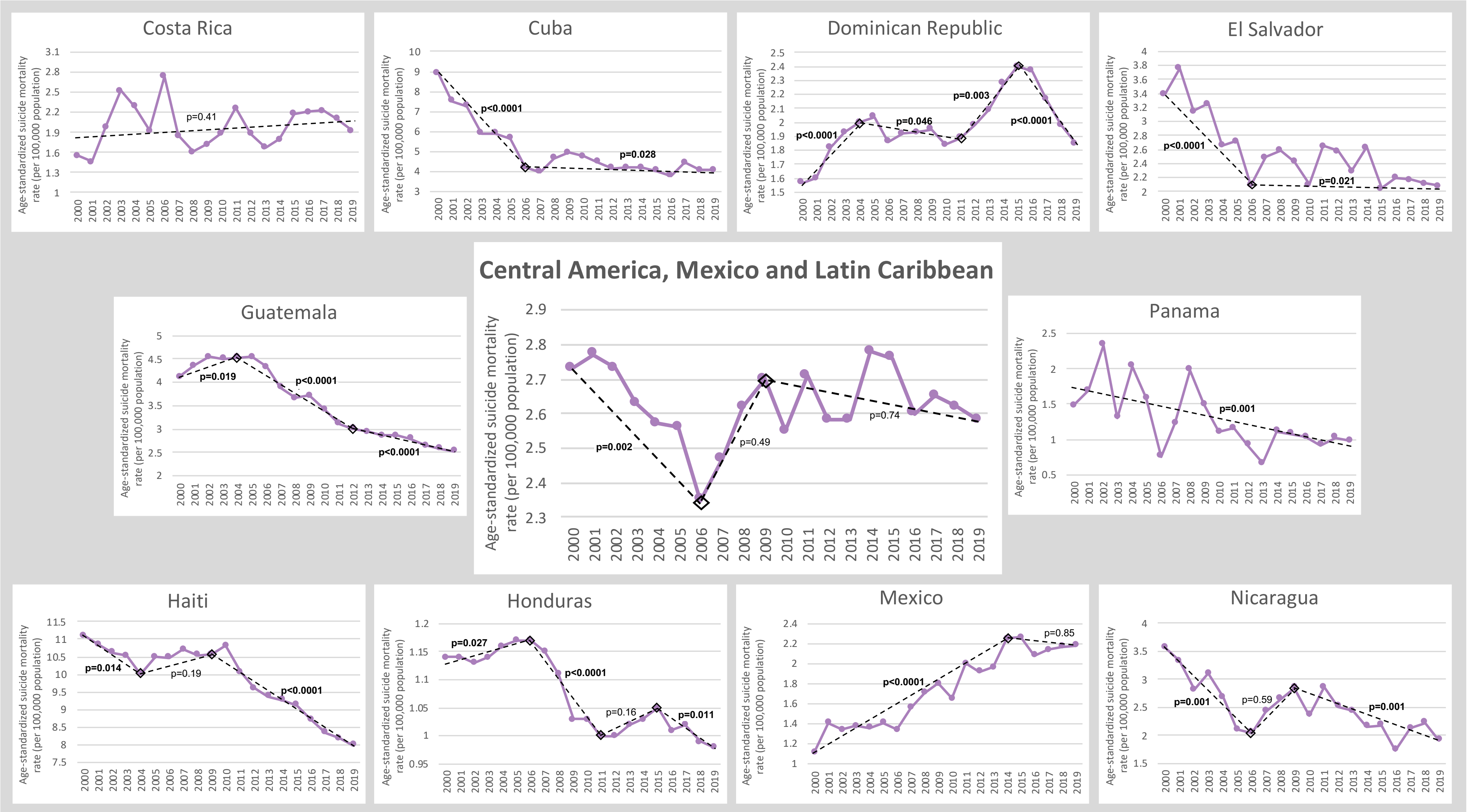

Supplementary Figure 6. Age-standardized suicide mortality rate among females, and the trend over time in the Central America, Mexico and Latin Caribbean sub-region and its countries, 2000-2019

Note. Diamonds indicate identified points of inflection. Statistically significant linear segments at p<0.05 are bolded.

Supplementary Table 5. Joinpoint analysis of age-standardized suicide mortality rates (per 100,000 people) over the past 20 years in the Non-Latin Caribbean sub-region, by country

Country	Sex	Mortality rate[a]		Total study period (2000-2019)		Period 1			Period 2			Period 3			Period 4		
		2000	2019	AAPC (%)	95% CI	Years	APC (%)	95% CI	Years	APC (%)	95% CI	Years	APC (%)	95% CI	Years	APC (%)	95% CI
Antigua and Barbuda																	
	M[b]	4.45	0	**-11.40**	**-15.91, -6.64**	**2000-11**	**-24.21**	**-28.91, -19.21**	2011-19	n/a	n/a						
	F[b]	0	0.60	0.76	-6.84, 8.97	2000-11	n/a	n/a	2011-14	45.74	-1.83, 116.38	**2014-19**	**-8.00**	**-13.45, -2.20**			
Bahamas																	
	M	4.20	5.75	**1.51**	**0.57, 2.45**	**2000-10**	**3.98**	**2.62, 5.35**	2010-19	-1.16	-2.68, 0.38	-	-	-	-	-	-
	F	1.11	1.23	0.29	-3.28, 4.00	2000-02	-13.79	-30.29, 6.62	2002-05	17.33	-4.22, 43.73	**2005-16**	**-2.82**	**-3.87, -1.76**	2016-19	6.47	-2.34, 16.07
Barbados																	
	M	4.85	0.48	-14.68	-37.73, 16.91	2000-02	-74.91	-95.89, 53.24	2002-05	82.50	-70.01, 1,010.58	**2005-19**	**-13.65**	**-21.55, -4.95**	-	-	-
	F[b]	0.45	0.16	**-5.79**	**-9.55, -1.86**	**2000-19**	**-5.79**	**-9.55, -1.86**									
Belize																	
	M	17.16	13.64	-0.90	-2.42, 0.63	**2000-07**	**-4.00**	**-7.45, -0.42**	2007-19	0.95	-0.63, 2.55	-	-	-	-	-	-
	F	2.93	1.83	**-2.34**	**-4.62, -0.01**	**2000-08**	**-5.28**	**-6.47, -4.08**	2008-11	4.45	-9.41, 20.42	**2011-16**	**-6.24**	**-9.81, -2.54**	2016-19	6.02	-0.62, 13.10
Grenada																	
	M[b]	3.82	0.53	-5.69	-14.76, 4.34	2000-04	55.45	-3.79, 151.15	**2004-19**	**-17.46**	**-21.87, -12.80**						
	F[b]	0.57	0.72	1.62	-11.46, 16.63	**2000-06**	**13.01**	**2.27, 24.88**	2006-09	-22.91	-69.84, 97.05	2009-19	3.58	-0.74, 0.09			
Guyana																	
	M	57.59	64.95	0.44	-0.37, 1.26	2000-02	4.12	-2.01, 10.62	**2002-08**	**-3.54**	**-4.75, -2.32**	**2008-13**	**4.44**	**2.57, 6.34**	2013-19	0.03	-0.91, 0.99
	F	14.45	16.95	0.30	-0.16, 0.76	2000-19	0.30	-0.16, 0.76	-	-	-	-	-	-	-	-	-
Jamaica																	
	M	3.26	3.63	0.03	-2.99, 3.14	**2000-05**	**-6.09**	**-9.66, -2.37**	2005-08	12.26	-8.25, 37.36	2008-19	-0.25	-1.35, 0.87	-	-	-
	F	0.89	1.00	0.61	-0.88, 2.12	**2000-06**	**-3.76**	**-5.16, -2.34**	2006-09	6.59	-3.39, 17.60	**2009-19**	**1.56**	**0.89, 2.23**	-	-	-
Saint Lucia																	
	M	14.52	12.51	-0.82	-3.41, 1.85	2000-10	-0.76	-1.57, 0.07	2010-13	3.61	-13.50, 24.12	**2013-19**	**-3.06**	**-4.92, -1.17**	-	-	-
	F	2.05	1.47	**-1.85**	**-3.34, -0.33**	**2000-03**	**-6.77**	**-10.46, -2.93**	2003-10	-0.75	-1.83, 0.33	2010-13	1.61	-7.93, 12.14	**2013-19**	**-2.29**	**-3.37, -1.18**
Saint Vincent and the Grenadines																	
	M[b]	12.59	1.33	-10.67	-27.68, 10.33	**2000-14**	**-7.65**	**-11.04, -4.12**	2014-17	-54.13	-86.28, 53.31	2017-19	92.27	-46.32, 588.64			
	F[b]	0.50	0.67	-2.87	-7.46, 1.95	2000-19	-2.87	-7.46, 1.95									
Suriname																	
	M	38.80	41.31	0.11	-0.57, 0.79	2000-02	4.85	-2.16, 12.37	**2002-19**	**-0.43**	**-0.58, -0.29**	-	-	-	-	-	-
	F	11.79	11.79	**-0.14**	**-0.28, -0.01**	**2000-19**	**-0.14**	**-0.28, -0.01**	-	-	-	-	-	-	-	-	-
Trinidad and Tobago																	
	M	26.38	13.08	**-3.47**	**-4.10, -2.83**	**2000-19**	**-3.47**	**-4.10, -2.83**	-	-	-	-	-	-	-	-	-
	F	6.32	3.65	**-2.84**	**-3.89, -1.78**	**2000-06**	**-7.60**	**-9.54, -5.63**	2006-13	1.78	-0.27, 3.87	**2013-19**	**-3.23**	**-5.25, -1.16**	-	-	-
Non-Latin Caribbean																	
	M	17.48	15.1	**-1.04**	**-1.45, -0.62**	**2000-06**	**-2.98**	**-4.14, -1.80**	2006-19	-0.13	-0.50, 0.24	-	-	-	-	-	-
	F	4.35	4.05	-0.48	-0.98, 0.02	**2000-06**	**-2.10**	**-3.52, -0.66**	2006-19	0.27	-0.15, 0.70	-	-	-	-	-	-

APC: Average annual percent change; APC: Annual percent change; CI: Confidence interval; F: Females; M: Males; n/a: Not available

[a]per 100,000 population

[b]A value of 0.5 per 100,000 population was imputed for one or more years that had an estimated suicide mortality rate of zero per 100,000 population.

Note. Bolded text indicates statistical significance ($p<0.05$). For Antigua and Barbuda, APC was not estimated for the linear segment following 2011 for males, and prior to 2011 for females as the suicide mortality rates was estimated to be zero per 100,000 population consistently during these periods.

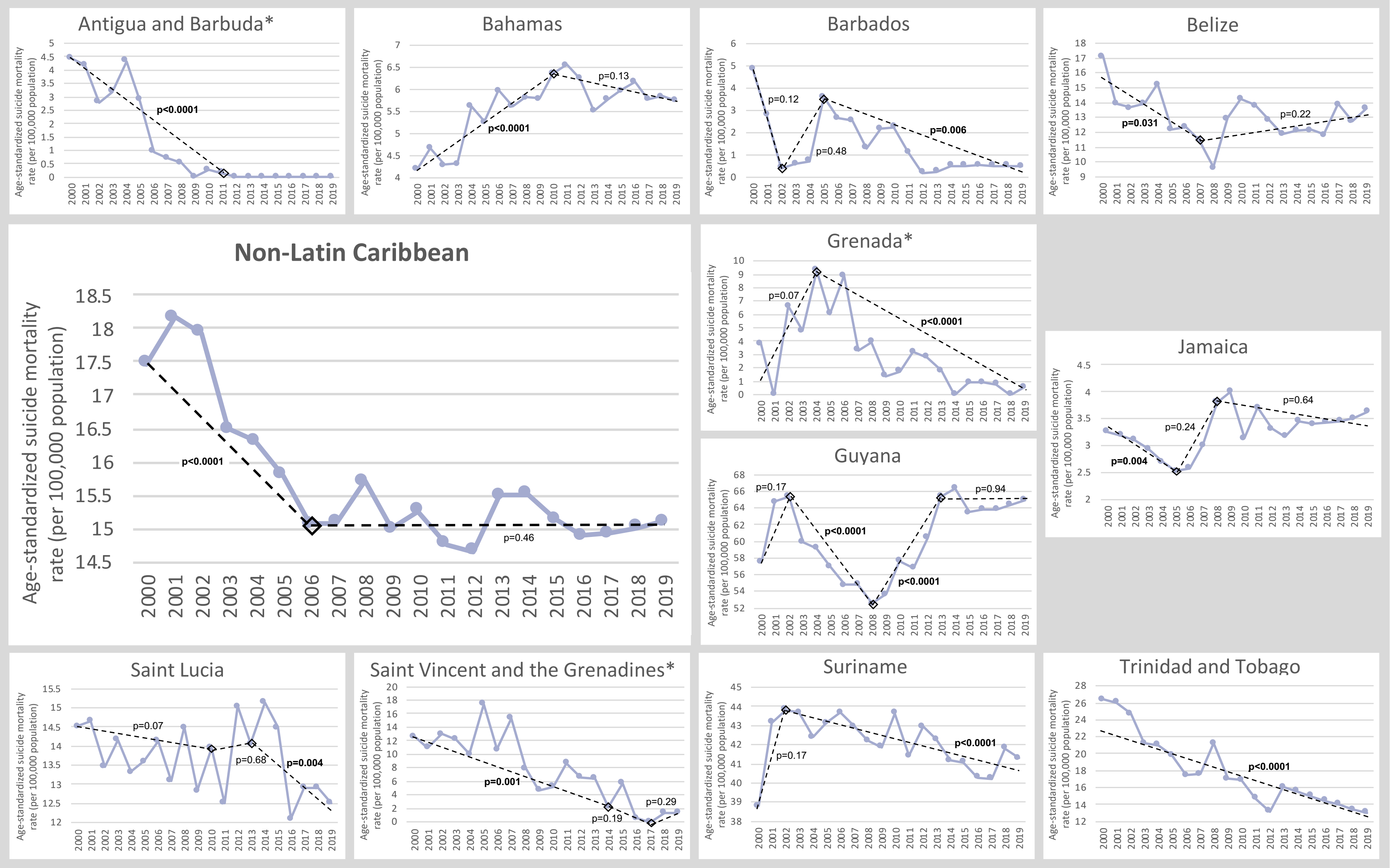

Supplementary Figure 7. Age-standardized suicide mortality rate among males, and the trend over time in the Non-Latin Caribbean sub-region and its countries, 2000-2019

Note. Diamonds indicate identified points of inflection. Statistically significant linear segments at p<0.05 are bolded.

*A value of 0.5 per 100,000 population was imputed for one or more years that had an estimated suicide mortality rate of zero per 100,000 population.

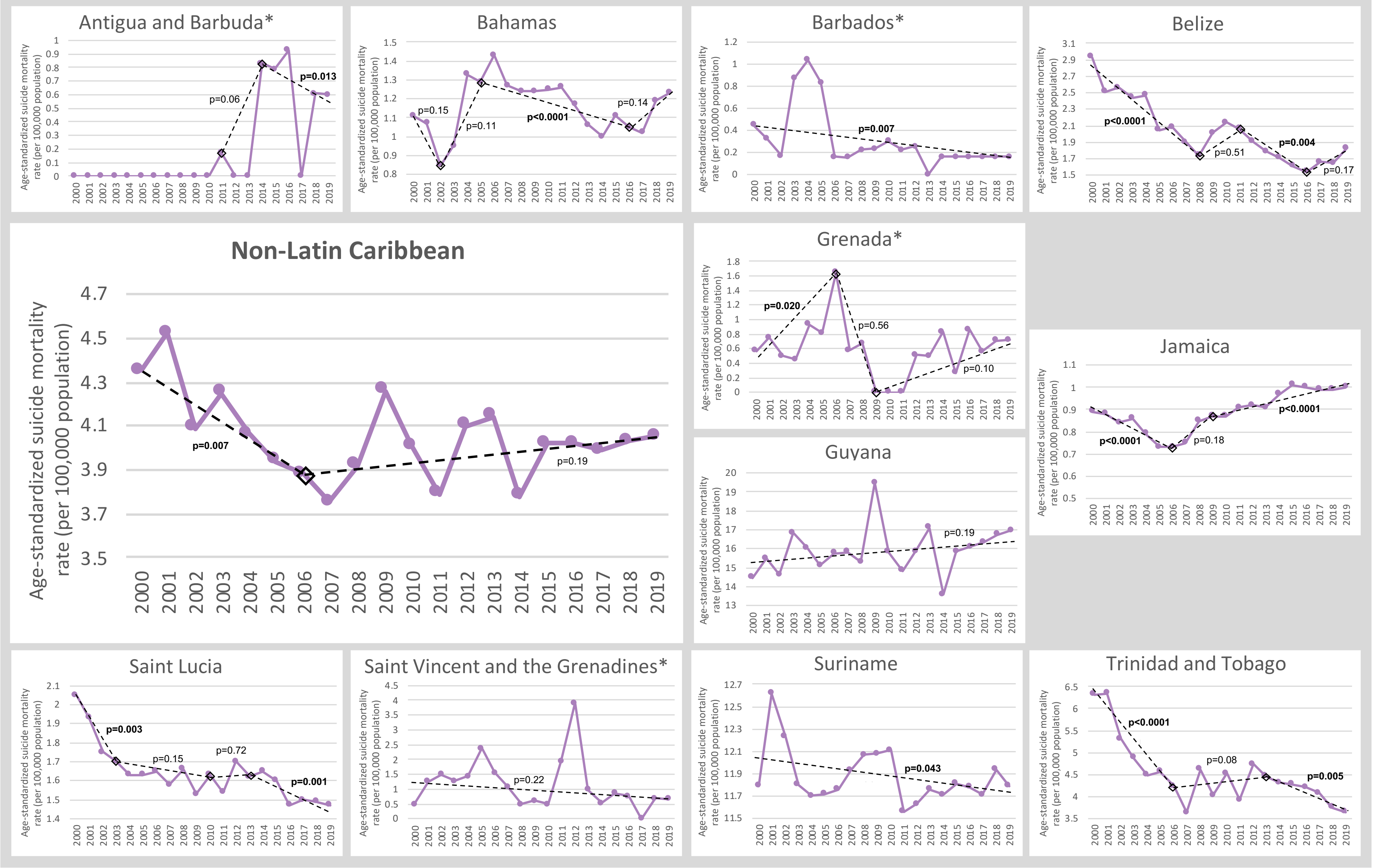

Supplementary Figure 8. Age-standardized suicide mortality rate among females, and the trend over time in the Non-Latin Caribbean sub-region and its countries, 2000-2019

Note. Diamonds indicate identified points of inflection. Statistically significant linear segments at p<0.05 are bolded.

*A value of 0.5 per 100,000 population was imputed for one or more years that had an estimated suicide mortality rate of zero per 100,000 population.

Supplementary Table 6. Joinpoint analysis of age-standardized suicide mortality rates (per 100,000 people) over the past 20 years in the North America sub-region, by country

Country	Sex	Mortality rate[a]		Total study period (2000-2019)		Period 1			Period 2			Period 3			Period 4		
		2000	2019	AAPC (%)	95% CI	Years	APC (%)	95% CI	Years	APC (%)	95% CI	Years	APC (%)	95% CI	Years	APC (%)	95% CI
Canada																	
	M	16.56	15.34	-0.28	-0.87, 0.31	**2000-07**	**-2.19**	**-3.57, -0.80**	**2007-19**	**0.85**	**0.25, 1.45**	-	-	-	-	-	-
	F	4.94	5.38	0.39	-0.78, 1.59	2000-11	0.38	-0.19, 0.95	2011-15	3.14	-2.04, 8.59	2015-19	-2.24	-5.21, 0.84	-	-	-
United States of America																	
	M	16.44	22.39	**1.54**	**0.83, 2.25**	**2000-14**	**1.03**	**0.86, 1.20**	**2014-17**	**5.12**	**0.94, 9.46**	2017-19	-0.15	-4.13, 3.98	-	-	-
	F	3.96	6.77	**2.87**	**1.98, 3.76**	**2000-03**	**4.13**	**1.87, 6.45**	**2003-13**	**2.24**	**1.93, 2.55**	2013-16	4.22	-1.25, 9.99	**2016-19**	**2.37**	**0.15, 4.65**
North America																	
	M	16.45	21.65	**1.36**	**0.58, 2.14**	**2000-14**	**0.87**	**0.71, 1.03**	**2014-17**	**5.12**	**0.56, 9.90**	2017-19	-0.68	-5.07, 3.92	-	-	-
	F	4.05	6.61	**2.67**	**2.33, 3.01**	**2000-04**	**3.50**	**2.43, 4.59**	**2004-09**	**1.60**	**0.57, 2.65**	**2009-19**	**2.87**	**2.63, 3.10**	-	-	-

APC: Average annual percent change; APC: Annual percent change; CI: Confidence interval; F: Females; M: Males

[a]per 100,000 population

Note. Bolded text indicates statistical significance (p<0.05).

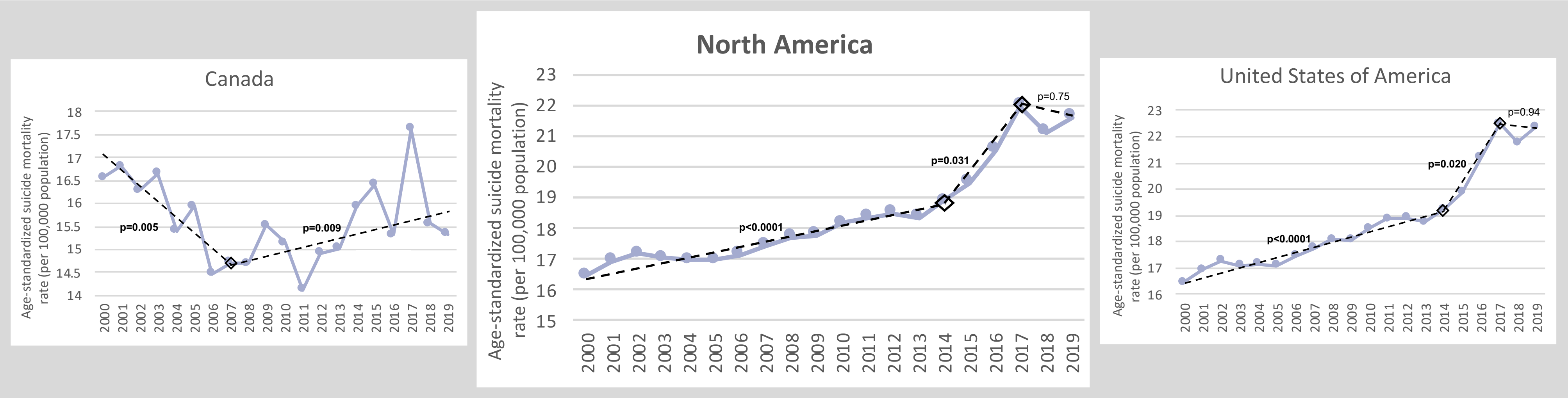

Supplementary Figure 9. Age-standardized suicide mortality rate among males, and the trend over time in the North America sub-region and its countries, 2000-2019
Note. Diamonds indicate identified points of inflection. Statistically significant linear segments at $p<0.05$ are bolded.

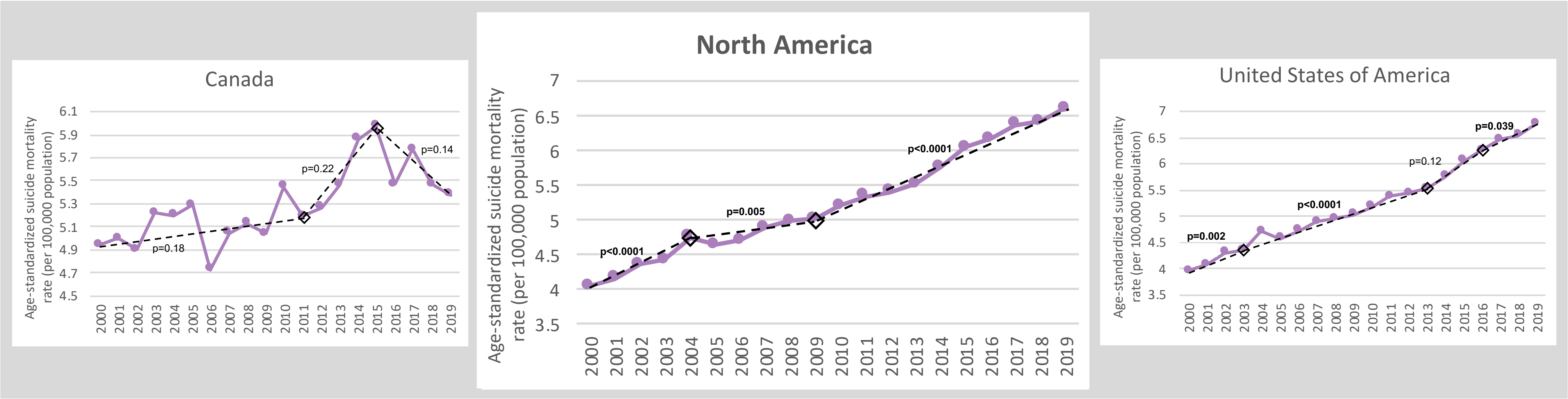

Supplementary Figure 10. Age-standardized suicide mortality rate among females, and the trend over time in the North America sub-region and its countries, 2000-2019
Note. Diamonds indicate identified points of inflection. Statistically significant linear segments at $p<0.05$ are bolded.

Supplementary Table 7. Joinpoint analysis of age-standardized suicide mortality rates (per 100,000 people) over the past 20 years in the Southern Cone sub-region, by country

Country	Sex	Mortality rate[a]		Total study period (2000-2019)		Period 1			Period 2			Period 3			Period 4		
		**2000**	**2019**	**AAPC (%)**	**95% CI**	**Years**	**APC (%)**	**95% CI**	**Years**	**APC (%)**	**95% CI**	**Years**	**APC (%)**	**95% CI**	**Years**	**APC (%)**	**95% CI**
Argentina																	
	M	15.95	13.47	**-1.10**	**-1.78, -0.41**	**2000-06**	**-3.43**	**-5.37, -1.45**	2006-19	-0.004	-0.59, 0.58	-	-	-	-	-	-
	F	3.38	3.30	-0.14	-2.40, 2.16	2000-02	8.26	-10.01, 30.25	2002-06	-6.06	-12.81, 1.22	2006-19	0.49	-0.16, 1.15	-	-	-
Brazil																	
	M	7.37	10.32	**1.80**	**1.34, 2.26**	**2000-14**	**0.87**	**0.54, 1.19**	**2014-19**	**4.45**	**2.74, 6.19**	-	-	-	-	-	-
	F	1.83	2.76	**1.86**	**1.60, 2.11**	**2000-19**	**1.86**	**1.60, 2.11**	-	-	-	-	-	-	-	-	-
Chile																	
	M	18.96	13.40	**-1.76**	**-3.34, -0.16**	**2000-05**	**-2.79**	**-5.08, -0.44**	2005-09	5.18	-0.55, 11.25	**2009-13**	**-5.73**	**-10.87, -0.30**	**2013-19**	**-2.68**	**-4.39, -0.93**
	F	2.89	2.98	-0.04	-3.08, 3.09	2000-05	0.94	-3.19, 5.26	2005-08	14.77	-6.08, 40.25	**2008-19**	**-4.17**	**-5.34, -2.98**	-	-	-
Paraguay																	
	M	4.83	9.00	**3.56**	**2.90, 4.22**	**2000-19**	**3.56**	**2.90, 4.22**	-	-	-	-	-	-	-	-	-
	F	2.46	3.26	**1.53**	**0.59, 2.49**	**2000-19**	**1.53**	**0.59, 2.49**	-	-	-	-	-	-	-	-	-
Uruguay																	
	M	25.66	31.11	**1.34**	**0.34, 2.35**	2000-09	-1.15	-2.77, 0.51	**2009-19**	**3.63**	**2.19, 5.09**	-	-	-	-	-	-
	F	5.07	7.67	**1.31**	**0.40, 2.23**	**2000-19**	**1.31**	**0.40, 2.23**	-	-	-	-	-	-	-	-	-
Southern Cone																	
	M	9.99	11.26	**0.72**	**0.27, 1.16**	2000-13	-0.08	-0.47, 0.30	**2013-19**	**2.46**	**1.15, 3.78**	-	-	-	-	-	-
	F	2.23	2.93	**1.10**	**0.83, 1.37**	**2000-19**	**1.10**	**0.83, 1.37**	-	-	-	-	-	-	-	-	-

APC: Average annual percent change; APC: Annual percent change; CI: Confidence interval; F: Females; M: Males

[a]per 100,000 population

Note. Bolded text indicates statistical significance (p<0.05).

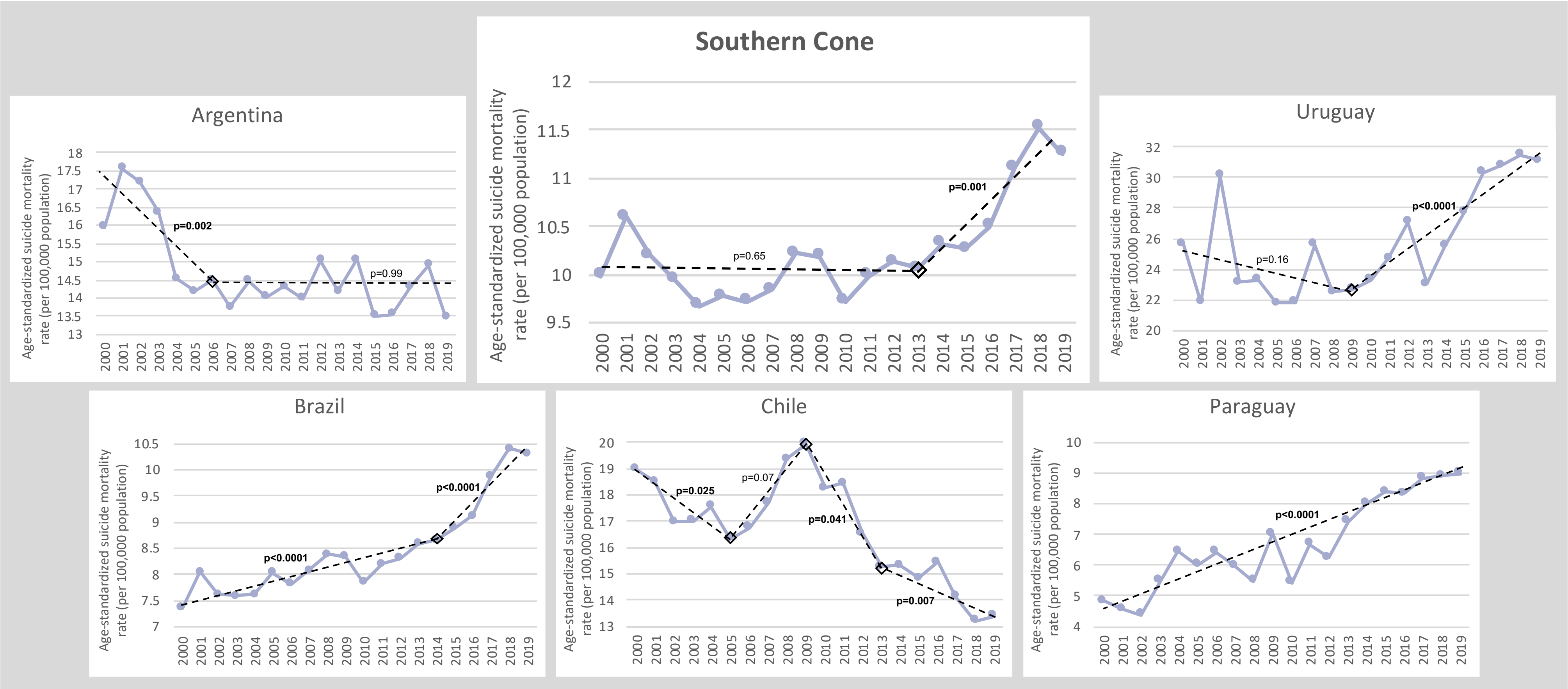

Supplementary Figure 11. Age-standardized suicide mortality rate among males, and the trend over time in the Southern Cone sub-region and its countries, 2000-2019
Note. Diamonds indicate identified points of inflection. Statistically significant linear segments at p<0.05 are bolded.

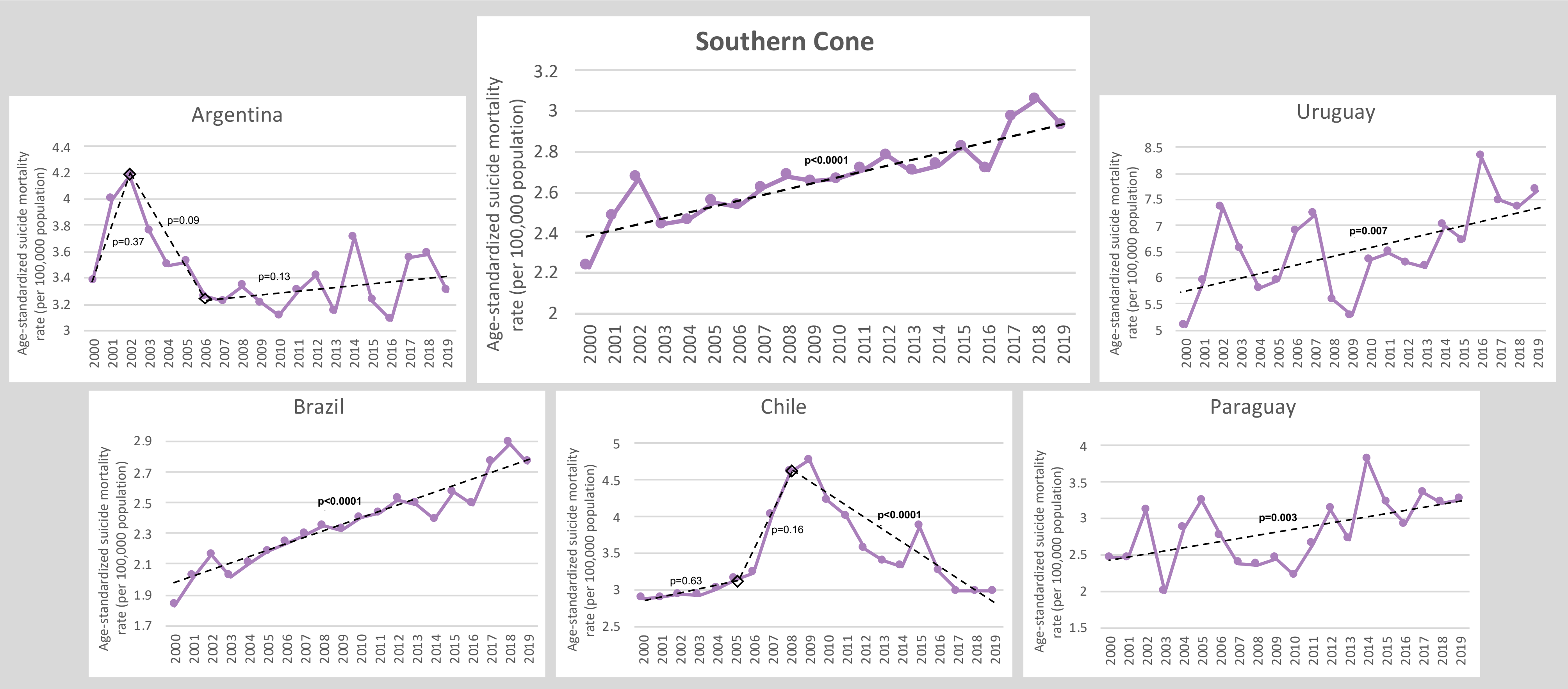

Supplementary Figure 12. Age-standardized suicide mortality rate among females, and the trend over time in the Southern Cone sub-region and its countries, 2000-2019
Note. Diamonds indicate identified points of inflection. Statistically significant linear segments at $p<0.05$ are bolded.

Supplementary Table 8. Observed and Forecasted Sex-specific Age-standardized Suicide Mortality Rates and 95% Confidence Intervals Among Males, 2000-2030

Location Name	Model	2000	2001	2002	2003	2004	2005	2006	2007	2008	2009	2010	2011	2012	2013	2014	2015	2016	2017	2018	2019	2020	2021	2022	2023	2024	2025	2026	2027	2028	2029	2030
**Andean Area**																																
Bolivia, Plurinational State of	linear	11.8 (7.0-18.0)	11.3 (6.7-17.3)	11.1 (6.5-16.9)	10.8 (6.2-16.4)	10.4 (6.0-15.9)	10.2 (5.9-15.6)	9.6 (5.6-14.8)	9.7 (5.6-15.0)	9.4 (5.4-14.6)	9.4 (5.4-14.6)	9.4 (5.4-14.7)	9.5 (5.4-14.9)	9.3 (5.3-14.6)	9.3 (5.2-14.7)	9.6 (5.3-15.2)	9.9 (5.5-15.7)	10.3 (5.6-16.5)	10.0 (5.4-15.9)	9.8 (5.2-15.5)	9.6 (5.1-15.2)	9.2 (8.7-9.8)	9.1 (8.5-9.7)	9.1 (8.4-9.7)	9.0 (8.3-9.6)	8.9 (8.2-9.6)	8.8 (8.1-9.6)	8.7 (7.9-9.5)	8.7 (7.8-9.5)	8.6 (7.7-9.5)	8.5 (7.6-9.4)	8.4 (7.5-9.4)
Colombia	linear	8.4 (7.2-14.1)	8.4 (7.2-14.0)	7.7 (6.6-19.1)	7.4 (6.4-15.2)	7.5 (6.5-21.4)	7.0 (6.0-18.1)	7.0 (6.0-16.1)	7.0 (6.0-16.1)	7.1 (6.1-15.1)	6.8 (5.9-7.9)	6.5 (5.5-14.0)	6.1 (5.2-16.1)	6.3 (5.4-15.5)	6.0 (5.1-13.1)	6.2 (5.2-12.1)	6.5 (5.4-12.5)	6.9 (5.6-13.3)	7.4 (5.6-18.5)	6.3 (4.4-15.1)	6.0 (4.1-15.0)	5.9 (5.5-6.4)	5.8 (5.3-6.3)	5.7 (5.2-6.3)	5.6 (5.1-6.2)	5.5 (4.9-6.2)	5.4 (4.8-6.1)	5.4 (4.7-6.0)	5.3 (4.5-6.0)	5.2 (4.4-5.9)	5.1 (4.3-5.9)	5.0 (4.1-5.8)
Ecuador	lowess & ARIMA	9.6 (6.7-12.1)	10.3 (7.1-13.0)	11.9 (8.1-15.1)	12.8 (8.6-16.2)	13.6 (8.8-17.1)	15.2 (9.4-19.1)	15.1 (9.1-19.0)	15.6 (9.1-19.7)	16.2 (9.4-20.5)	16.1 (9.3-20.4)	15.1 (8.5-19.1)	15.0 (8.4-19.1)	15.6 (8.5-19.8)	14.7 (8.1-18.6)	13.5 (7.2-17.1)	13.7 (7.2-17.5)	14.0 (7.3-18.1)	14.4 (7.4-19.1)	12.5 (6.3-17.3)	11.9 (6.0-17.0)	12.3 (11.0-13.6)	12.3 (10.4-14.1)	12.2 (10.0-14.4)	12.2 (9.7-14.7)	12.2 (9.5-14.9)	12.2 (9.2-15.1)	12.2 (9.0-15.3)	12.1 (8.9-15.4)	12.1 (8.7-15.5)	12.1 (8.6-15.6)	12.1 (8.4-15.7)
Peru	lowess & ARIMA	4.6 (3.0-6.2)	4.3 (2.8-5.8)	4.4 (2.7-5.9)	4.5 (2.8-6.1)	4.3 (2.7-5.9)	4.2 (2.5-5.7)	4.1 (2.5-5.6)	4.1 (2.5-5.6)	4.2 (2.6-5.8)	5.0 (3.1-6.9)	5.4 (3.3-7.3)	5.1 (3.1-7.0)	5.0 (3.1-6.8)	4.8 (3.0-6.6)	4.5 (2.8-6.2)	4.2 (2.5-5.9)	4.0 (2.4-5.8)	4.1 (2.4-6.2)	4.1 (2.4-6.3)	4.1 (2.4-6.4)	3.9 (3.8-4.0)	4.0 (3.8-4.2)	4.1 (3.8-4.4)	4.3 (3.9-4.7)	4.6 (4.1-5.0)	4.7 (4.3-5.2)	4.9 (4.4-5.3)	5.0 (4.5-5.4)	4.9 (4.5-5.4)	4.9 (4.4-5.4)	4.7 (4.2-5.3)
Venezuela, Bolivarian Republic of	linear	11.3 (9.6-20.1)	11.5 (9.7-20.6)	10.7 (9.0-22.9)	9.7 (8.1-11.4)	8.7 (7.3-10.3)	8.3 (6.9-17.4)	7.5 (6.2-8.9)	7.0 (5.8-8.3)	6.5 (5.4-15.0)	5.9 (4.8-7.1)	6.3 (5.1-7.6)	6.9 (5.5-19.3)	6.0 (4.8-7.4)	4.5 (3.6-5.5)	4.0 (3.1-5.0)	4.1 (3.0-5.4)	3.9 (2.8-5.4)	3.9 (2.6-5.4)	3.9 (2.6-5.5)	3.6 (2.4-5.2)	2.2 (1.5-2.9)	1.8 (1.1-2.5)	1.4 (0.6-2.2)	0.9 (0.1-1.8)	0.5 (-0.4-1.4)	0.1 (-0.9-1.0)	-0.3 (-1.3-0.7)	-0.8 (-1.8-0.3)	-1.2 (-2.3--0.1)	-1.6 (-2.8--0.4)	-2.0 (-3.3--0.8)
**Central America, Mexico and Latin Caribbean**																																
Costa Rica	linear	12.3 (10.3-14.4)	9.8 (8.2-11.5)	12.7 (10.7-14.8)	14.0 (11.8-16.4)	14.5 (12.3-16.9)	11.5 (9.8-13.5)	14.2 (12.0-35.9)	9.5 (8.1-11.1)	9.4 (8.0-11.0)	9.5 (8.0-11.1)	11.7 (9.9-13.6)	11.2 (9.5-13.1)	10.9 (9.1-33.3)	11.2 (9.4-13.2)	8.9 (7.5-10.6)	10.7 (8.8-12.8)	12.4 (10.1-15.0)	9.4 (7.2-32.7)	11.5 (8.3-25.0)	13.3 (9.3-18.3)	10.7 (9.0-12.4)	10.6 (8.8-12.4)	10.6 (8.6-12.5)	10.5 (8.4-12.5)	10.4 (8.2-12.6)	10.4 (8.0-12.7)	10.3 (7.8-12.7)	10.2 (7.6-12.8)	10.1 (7.4-12.8)	10.1 (7.2-12.9)	10.0 (7.0-13.0)
Cuba	linear	22.7 (20.0-39.7)	20.3 (17.9-22.9)	18.9 (16.6-21.3)	18.4 (16.2-20.8)	18.5 (16.2-20.8)	16.9 (14.8-19.1)	17.4 (15.3-19.7)	16.2 (14.2-18.3)	16.5 (14.5-18.6)	17.6 (15.4-19.8)	18.6 (16.3-20.9)	18.2 (15.9-20.6)	17.7 (15.5-20.1)	17.5 (15.3-19.9)	16.6 (14.4-19.0)	17.1 (14.7-19.7)	16.0 (13.6-18.8)	17.0 (14.2-35.7)	16.6 (13.0-29.4)	16.7 (12.3-30.7)	15.9 (14.7-17.0)	15.7 (14.5-16.9)	15.5 (14.2-16.8)	15.3 (13.9-16.7)	15.2 (13.7-16.7)	15.0 (13.4-16.6)	14.8 (13.1-16.5)	14.6 (12.8-16.4)	14.4 (12.6-16.3)	14.3 (12.3-16.2)	14.1 (12.0-16.1)
Dominican Republic	ARIMA	8.3 (5.8-11.7)	8.0 (5.5-11.2)	8.0 (5.6-11.4)	8.8 (6.1-12.4)	8.7 (6.1-12.1)	9.2 (6.3-12.9)	9.4 (6.4-13.4)	9.5 (6.4-13.8)	9.4 (6.2-13.9)	9.4 (6.3-13.9)	9.0 (6.1-13.1)	9.2 (6.4-13.4)	9.7 (6.7-14.1)	10.2 (6.9-14.9)	11.1 (7.3-16.6)	11.4 (7.3-17.4)	11.3 (7.1-17.6)	10.0 (6.0-15.9)	9.2 (5.4-14.9)	8.5 (4.9-14.1)	8.2 (7.3-9.1)	8.0 (6.3-9.7)	7.9 (5.6-10.3)	7.9 (4.9-10.8)	7.8 (4.3-11.4)	7.8 (3.8-11.8)	7.8 (3.4-12.3)	7.8 (3.0-12.7)	7.8 (2.6-13.0)	7.8 (2.3-13.4)	7.8 (1.9-13.7)
El Salvador	lowess & ARIMA	10.6 (8.3-13.8)	11.7 (9.1-15.4)	9.3 (7.2-12.4)	12.0 (9.2-15.9)	12.9 (9.9-17.1)	14.7 (11.2-32.4)	12.7 (9.7-17.4)	15.7 (11.9-35.3)	13.0 (9.8-32.6)	16.8 (12.6-22.5)	11.7 (8.7-30.1)	14.4 (10.8-19.0)	12.4 (9.0-16.6)	12.3 (8.7-30.8)	10.1 (7.1-13.4)	12.5 (8.6-17.5)	10.2 (6.7-22.6)	11.1 (7.1-24.6)	11.0 (6.7-24.4)	11.1 (6.7-24.8)	11.2 (11.1-11.4)	11.4 (11.0-11.9)	11.7 (10.9-12.5)	12.0 (10.8-13.1)	12.2 (10.7-13.7)	12.4 (10.7-14.2)	12.6 (10.6-14.6)	12.7 (10.6-14.9)	12.8 (10.5-15.0)	12.8 (10.5-15.0)	12.7 (10.4-14.9)
Guatemala	linear	24.0 (18.6-30.3)	25.1 (19.5-31.6)	24.0 (18.6-30.3)	24.4 (18.9-30.9)	24.6 (18.9-31.1)	25.0 (19.4-31.7)	23.4 (18.2-29.7)	17.7 (13.8-22.4)	16.9 (13.2-21.4)	16.0 (12.4-20.1)	14.6 (11.4-18.3)	12.7 (10.0-15.9)	11.6 (9.1-14.4)	10.9 (8.6-13.7)	10.4 (8.2-13.1)	10.4 (8.1-13.1)	10.0 (7.7-12.8)	9.8 (7.4-12.7)	10.4 (7.6-14.0)	10.3 (7.2-14.2)	6.1 (4.0-8.2)	5.1 (2.9-7.3)	4.1 (1.7-6.5)	3.1 (0.5-5.7)	2.1 (-0.6-4.8)	1.1 (-1.8-4.0)	0.1 (-2.9-3.1)	-0.9 (-4.1-2.3)	-1.9 (-5.3-1.5)	-2.9 (-6.4-0.6)	-3.9 (-7.6--0.2)
Haiti	ARIMA	14.7 (7.3-23.5)	14.2 (7.1-22.9)	13.9 (6.9-22.3)	13.9 (6.8-22.2)	13.7 (6.7-22.1)	13.9 (6.8-22.5)	14.2 (6.9-22.9)	14.9 (7.3-24.0)	15.1 (7.4-24.5)	15.4 (7.6-25.1)	16.0 (7.8-26.4)	15.3 (7.5-25.2)	15.2 (7.5-25.1)	15.2 (7.4-25.1)	15.2 (7.4-25.4)	15.3 (7.5-25.4)	15.1 (7.3-25.0)	15.1 (7.3-24.9)	15.1 (7.4-25.1)	14.9 (7.3-24.8)	14.9 (14.2-15.5)	14.9 (13.9-15.8)	14.9 (13.7-16.0)	14.9 (13.5-16.2)	14.9 (13.4-16.4)	14.9 (13.2-16.5)	14.9 (13.1-16.6)	14.9 (13.0-16.8)	14.9 (12.9-16.9)	14.9 (12.8-17.0)	14.9 (12.7-17.1)
Honduras	ARIMA	5.1 (2.8-9.9)	5.0 (2.8-9.9)	5.1 (2.8-10.2)	5.1 (2.7-10.3)	5.0 (2.7-10.1)	5.0 (2.6-10.1)	5.0 (2.6-10.0)	4.9 (2.6-9.8)	4.8 (2.5-9.6)	4.6 (2.4-9.3)	4.5 (2.3-9.0)	4.4 (2.3-8.8)	7.1 (3.6-14.7)	7.0 (3.6-14.4)	7.3 (3.7-15.0)	8.3 (4.1-17.2)	4.5 (2.3-9.0)	4.6 (2.4-9.2)	4.4 (2.3-8.7)	4.4 (2.2-8.7)	4.8 (2.8-6.8)	5.0 (2.8-7.2)	5.1 (2.9-7.4)	5.2 (2.9-7.5)	5.2 (2.9-7.5)	5.2 (2.9-7.5)	5.2 (2.9-7.5)	5.2 (3.0-7.5)	5.3 (3.0-7.5)	5.3 (3.0-7.5)	5.3 (3.0-7.5)
Mexico	linear	6.8 (6.5-17.0)	7.2 (6.8-12.4)	7.2 (6.9-16.8)	7.4 (7.1-7.8)	7.4 (7.1-7.8)	7.6 (7.2-7.9)	7.4 (7.1-7.8)	7.3 (7.0-7.7)	7.7 (7.3-8.1)	8.1 (7.7-8.5)	8.0 (7.6-8.3)	9.0 (8.6-9.4)	8.2 (7.8-8.6)	8.9 (8.5-9.3)	9.5 (9.0-9.9)	9.2 (8.8-9.7)	9.4 (8.9-9.8)	9.9 (9.3-10.6)	8.8 (7.6-10.2)	8.7 (7.1-10.6)	9.7 (9.2-10.1)	9.8 (9.4-10.2)	9.9 (9.5-10.4)	10.1 (9.6-10.6)	10.2 (9.7-10.7)	10.3 (9.8-10.9)	10.5 (9.9-11.1)	10.6 (10.0-11.2)	10.8 (10.1-11.4)	10.9 (10.2-11.6)	11.0 (10.3-11.8)
Nicaragua	linear	9.3 (5.9-11.8)	9.5 (6.2-12.1)	9.3 (6.0-11.8)	10.7 (6.9-13.6)	10.8 (7.0-13.8)	10.7 (6.9-13.6)	8.9 (5.9-11.4)	8.7 (5.7-11.0)	9.0 (6.0-11.4)	8.5 (5.7-10.7)	8.9 (6.1-11.3)	9.5 (6.5-30.3)	7.5 (5.2-28.7)	9.8 (6.9-30.9)	8.2 (5.8-10.8)	7.1 (5.1-9.5)	7.8 (5.5-10.4)	7.7 (5.5-10.3)	8.3 (5.7-27.3)	7.8 (5.1-21.0)	7.5 (6.8-8.3)	7.4 (6.6-8.2)	7.3 (6.4-8.1)	7.1 (6.2-8.1)	7.0 (6.0-8.0)	6.9 (5.8-7.9)	6.8 (5.6-7.9)	6.6 (5.5-7.8)	6.5 (5.3-7.7)	6.4 (5.1-7.6)	6.2 (4.9-7.6)
Panama	linear	10.4 (8.5-12.6)	10.6 (8.7-12.8)	9.4 (7.7-11.4)	11.4 (9.3-13.7)	10.2 (8.3-12.2)	9.1 (7.4-11.0)	11.2 (9.2-13.6)	9.1 (7.5-10.9)	9.2 (7.6-11.1)	8.7 (7.1-10.5)	7.6 (6.2-9.1)	6.8 (5.5-8.2)	6.6 (5.4-8.1)	6.5 (5.2-7.9)	7.2 (5.8-8.9)	4.9 (3.9-6.0)	5.7 (4.5-7.2)	5.5 (4.1-7.3)	4.9 (3.4-6.8)	4.8 (3.2-6.8)	4.3 (3.5-5.1)	4.0 (3.1-4.8)	3.6 (2.7-4.5)	3.3 (2.3-4.2)	2.9 (1.9-4.0)	2.6 (1.5-3.7)	2.2 (1.1-3.4)	1.9 (0.7-3.1)	1.5 (0.3-2.8)	1.2 (-0.2-2.5)	0.8 (-0.6-2.2)
**Non-Latin Caribbean**																																
Antigua and Barbuda	linear	4.5 (3.1-6.1)	4.2 (2.9-5.7)	2.8 (1.9-3.8)	3.2 (2.3-4.3)	4.3 (3.1-5.9)	2.9 (2.0-3.9)	0.9 (0.7-1.3)	0.7 (0.5-1.0)	0.5 (0.4-0.7)	0.0 (0.0-0.0)	0.3 (0.2-0.4)	0.1 (0.1-0.2)	0.0 (0.0-0.0)	0.0 (0.0-0.0)	0.0 (0.0-0.0)	0.0 (0.0-0.0)	0.0 (0.0-0.0)	0.0 (0.0-0.0)	0.0 (0.0-0.0)	0.0 (0.0-0.0)	-1.3 (-2.2--0.4)	-1.6 (-2.5--0.6)	-1.8 (-2.8--0.8)	-2.1 (-3.1--1.0)	-2.3 (-3.5--1.1)	-2.5 (-3.8--1.3)	-2.8 (-4.1--1.5)	-3.0 (-4.4--1.7)	-3.3 (-4.7--1.8)	-3.5 (-5.0--2.0)	-3.8 (-5.3--2.2)
Bahamas	linear	4.2 (3.1-5.5)	4.7 (3.5-6.2)	4.3 (3.2-5.6)	4.3 (3.2-5.7)	5.6 (4.2-7.4)	5.3 (3.9-6.9)	6.0 (4.4-7.8)	5.6 (4.2-7.3)	5.8 (4.3-7.6)	5.8 (4.3-7.6)	6.4 (4.7-8.4)	6.6 (4.8-8.7)	6.2 (4.6-8.3)	5.5 (4.0-7.3)	5.8 (4.2-7.7)	6.0 (4.3-8.1)	6.2 (4.4-8.4)	5.8 (4.1-8.0)	5.8 (4.1-8.2)	5.8 (4.0-8.1)	6.4 (5.9-6.9)	6.5 (6.0-7.0)	6.6 (6.0-7.2)	6.7 (6.0-7.3)	6.7 (6.1-7.4)	6.8 (6.1-7.5)	6.9 (6.2-7.6)	7.0 (6.2-7.8)	7.1 (6.3-7.9)	7.1 (6.3-8.0)	7.2 (6.3-8.1)
Barbados	linear	4.8 (3.6-6.3)	2.8 (2.1-3.6)	0.4 (0.3-0.5)	0.6 (0.5-0.8)	0.7 (0.6-0.9)	3.6 (2.7-4.6)	2.6 (2.0-3.4)	2.5 (1.9-3.3)	1.3 (1.0-1.7)	2.2 (1.6-2.8)	2.2 (1.7-2.9)	1.1 (0.8-1.4)	0.2 (0.1-0.2)	0.2 (0.2-0.3)	0.5 (0.4-0.7)	0.5 (0.4-0.7)	0.5 (0.4-0.7)	0.5 (0.4-0.7)	0.5 (0.4-0.7)	0.5 (0.3-0.7)	-0.0 (-1.0-1.0)	-0.1 (-1.2-1.0)	-0.3 (-1.5-0.9)	-0.4 (-1.7-0.9)	-0.5 (-1.9-0.8)	-0.7 (-2.1-0.8)	-0.8 (-2.3-0.7)	-0.9 (-2.5-0.6)	-1.1 (-2.8-0.6)	-1.2 (-3.0-0.5)	-1.4 (-3.2-0.5)
Belize	linear	17.2 (13.1-21.9)	13.9 (10.6-17.8)	13.7 (10.4-17.5)	13.9 (10.7-17.8)	15.2 (11.7-19.5)	12.2 (9.3-15.6)	12.3 (9.5-15.8)	11.5 (8.8-14.7)	9.7 (7.4-12.3)	13.0 (9.9-16.6)	14.3 (10.9-18.2)	13.8 (10.6-17.7)	12.8 (9.8-16.5)	11.9 (9.1-15.3)	12.1 (9.2-15.6)	12.2 (9.2-15.7)	11.9 (8.9-15.5)	13.9 (10.3-18.3)	12.8 (9.4-16.9)	13.6 (9.9-18.2)	12.1 (10.6-13.6)	12.0 (10.4-13.6)	11.9 (10.2-13.6)	11.8 (10.0-13.6)	11.7 (9.8-13.6)	11.6 (9.6-13.6)	11.5 (9.4-13.6)	11.4 (9.2-13.7)	11.3 (9.0-13.7)	11.2 (8.8-13.7)	11.1 (8.5-13.7)
Grenada	linear	3.8 (2.9-5.0)	0.0 (0.0-0.0)	6.6 (5.0-8.5)	4.8 (3.6-6.2)	9.3 (7.0-12.0)	6.0 (4.6-7.8)	8.9 (6.7-11.6)	3.3 (2.5-4.3)	3.9 (3.0-5.1)	1.4 (1.1-1.8)	1.7 (1.3-2.2)	3.2 (2.4-4.1)	2.8 (2.1-3.6)	1.8 (1.3-2.3)	0.0 (0.0-0.0)	0.9 (0.7-1.1)	0.9 (0.7-1.1)	0.8 (0.6-1.0)	0.0 (0.0-0.0)	0.5 (0.4-0.7)	-0.1 (-2.4-2.1)	-0.4 (-2.8-1.9)	-0.7 (-3.3-1.8)	-1.0 (-3.8-1.7)	-1.4 (-4.2-1.5)	-1.7 (-4.7-1.4)	-2.0 (-5.2-1.3)	-2.3 (-5.7-1.2)	-2.6 (-6.1-1.0)	-2.9 (-6.6-0.9)	-3.2 (-7.1-0.8)
Guyana	ARIMA	57.6 (44.5-89.4)	64.8 (50.3-82.1)	65.4 (50.7-109.8)	59.9 (46.6-105.3)	59.2 (46.1-98.7)	56.9 (44.6-93.5)	54.8 (43.0-90.3)	54.9 (43.2-93.2)	52.5 (41.5-65.4)	53.6 (42.3-67.0)	57.7 (45.3-99.2)	56.8 (44.3-99.9)	60.4 (46.8-106.0)	65.4 (50.0-84.2)	66.4 (50.2-86.3)	63.5 (46.7-99.8)	63.8 (45.7-101.8)	63.8 (44.7-102.8)	64.4 (44.1-105.2)	65.0 (43.6-107.5)	63.4 (58.2-68.6)	62.0 (53.7-70.3)	61.2 (52.2-70.2)	60.8 (51.5-70.0)	60.5 (51.2-69.9)	60.4 (51.1-69.7)	60.3 (51.0-69.7)	60.3 (50.9-69.6)	60.3 (50.9-69.6)	60.2 (50.9-69.6)	60.2 (50.9-69.6)
Jamaica	ARIMA	3.3 (2.5-4.2)	3.2 (2.4-4.1)	3.1 (2.4-4.0)	2.9 (2.3-3.8)	2.7 (2.1-3.5)	2.5 (1.9-3.2)	2.6 (2.0-3.3)	3.0 (2.3-3.8)	3.8 (2.9-5.0)	4.0 (3.0-5.1)	3.1 (2.4-4.0)	3.7 (2.8-4.7)	3.3 (2.5-4.3)	3.2 (2.4-4.1)	3.5 (2.6-4.5)	3.4 (2.5-4.5)	3.4 (2.5-4.6)	3.5 (2.4-4.8)	3.5 (2.4-4.9)	3.6 (2.5-5.1)	3.5 (2.8-4.1)	3.4 (2.7-4.1)	3.4 (2.6-4.1)	3.3 (2.6-4.1)	3.3 (2.5-4.1)	3.3 (2.5-4.1)	3.3 (2.5-4.1)	3.3 (2.5-4.1)	3.3 (2.5-4.1)	3.3 (2.5-4.1)	3.3 (2.5-4.1)
Saint Lucia	lowess & ARIMA	14.5 (11.1-18.7)	14.7 (11.2-18.9)	13.5 (10.2-17.4)	14.2 (10.8-18.2)	13.3 (10.2-17.2)	13.6 (10.4-17.5)	14.1 (10.8-18.2)	13.1 (10.0-16.8)	14.5 (11.0-18.7)	12.8 (9.7-16.6)	14.0 (10.5-18.1)	12.5 (9.4-16.2)	15.0 (11.3-19.6)	14.1 (10.5-18.4)	15.1 (11.2-19.9)	14.5 (10.7-19.1)	12.1 (8.9-16.0)	12.9 (9.4-17.2)	12.9 (9.3-17.4)	12.5 (8.9-16.9)	11.6 (11.5-11.7)	11.1 (10.8-11.5)	10.6 (9.9-11.4)	10.2 (9.1-11.4)	9.8 (8.2-11.5)	9.5 (7.4-11.6)	9.2 (6.6-11.8)	9.0 (5.8-12.1)	8.7 (5.1-12.4)	8.5 (4.4-12.7)	8.3 (3.7-13.0)
Saint Vincent and the Grenadines	linear	12.6 (9.4-16.2)	11.0 (8.3-14.2)	12.9 (9.7-16.8)	12.2 (9.2-15.7)	10.0 (7.5-12.9)	17.4 (13.1-22.6)	10.7 (8.0-13.9)	15.3 (11.4-20.0)	8.0 (6.0-10.2)	4.7 (3.6-6.0)	5.2 (3.8-6.8)	8.7 (6.5-11.4)	6.6 (5.0-8.5)	6.4 (4.8-8.3)	2.2 (1.7-2.9)	5.7 (4.2-7.6)	0.6 (0.4-0.7)	0.0 (0.0-0.0)	1.4 (1.0-1.8)	1.3 (1.0-1.8)	-0.1 (-2.8-2.6)	-0.8 (-3.7-2.0)	-1.6 (-4.6-1.5)	-2.3 (-5.6-1.0)	-3.0 (-6.5-0.4)	-3.8 (-7.4--0.1)	-4.5 (-8.4--0.6)	-5.2 (-9.3--1.1)	-6.0 (-10.3--1.7)	-6.7 (-11.2--2.2)	-7.4 (-12.2--2.7)
Suriname	linear	38.8 (28.9-51.8)	43.2 (32.1-57.3)	43.8 (32.8-57.5)	43.7 (32.8-56.8)	42.4 (31.9-54.9)	43.1 (32.9-55.8)	43.7 (33.3-56.3)	43.0 (32.8-55.3)	42.2 (32.1-54.0)	41.9 (31.6-53.7)	43.6 (32.9-56.1)	41.4 (31.1-53.5)	43.0 (32.2-55.8)	42.3 (31.6-55.3)	41.2 (30.6-54.1)	41.1 (30.0-54.5)	40.3 (28.9-54.4)	40.2 (28.5-54.8)	41.8 (29.1-57.9)	41.3 (28.2-58.2)	41.2 (39.9-42.4)	41.1 (39.7-42.4)	41.0 (39.6-42.5)	40.9 (39.4-42.5)	40.8 (39.2-42.5)	40.7 (39.0-42.5)	40.7 (38.8-42.5)	40.6 (38.6-42.5)	40.5 (38.5-42.5)	40.4 (38.3-42.5)	40.3 (38.1-42.5)
Trinidad and Tobago	linear	26.4 (22.0-31.3)	26.1 (21.7-31.0)	24.7 (20.6-29.3)	21.2 (17.6-25.4)	21.0 (17.5-25.1)	19.8 (16.4-23.6)	17.4 (14.4-20.9)	17.6 (14.6-21.1)	21.2 (17.4-25.5)	17.0 (13.9-20.7)	16.9 (13.8-20.6)	14.7 (11.8-18.1)	13.2 (10.5-16.5)	16.1 (12.1-20.8)	15.6 (11.2-21.2)	15.0 (10.4-21.1)	14.4 (9.9-20.5)	14.0 (9.4-20.1)	13.4 (8.9-19.2)	13.1 (8.7-18.9)	11.1 (9.3-12.8)	10.4 (8.6-12.3)	9.8 (7.8-11.8)	9.1 (7.0-11.2)	8.5 (6.2-10.7)	7.8 (5.4-10.2)	7.1 (4.6-9.7)	6.5 (3.8-9.2)	5.8 (3.0-8.6)	5.2 (2.3-8.1)	4.5 (1.5-7.6)
**North America**																																
Canada	lowess & ARIMA	16.6 (14.9-18.3)	16.8 (15.2-18.5)	16.3 (14.7-18.0)	16.6 (15.0-18.4)	15.4 (13.9-17.1)	15.9 (14.3-26.8)	14.5 (13.0-16.1)	14.7 (13.2-16.3)	14.7 (13.2-16.3)	15.5 (13.9-17.2)	15.1 (13.6-16.8)	14.1 (12.6-25.1)	14.9 (13.3-16.6)	15.0 (13.3-16.7)	15.9 (14.0-17.9)	16.4 (14.3-18.7)	15.3 (13.1-17.7)	17.6 (14.8-20.6)	15.6 (12.9-18.5)	15.3 (12.5-18.4)	15.8 (15.7-15.9)	15.8 (15.5-16.0)	15.7 (15.3-16.1)	15.7 (15.1-16.2)	15.6 (15.0-16.2)	15.6 (14.8-16.3)	15.5 (14.7-16.4)	15.5 (14.6-16.4)	15.5 (14.6-16.5)	15.6 (14.6-16.5)	15.6 (14.6-16.6)

United States	linear	16.4 (15.9-23.5)	16.9 (16.4-24.5)	17.3 (16.7-22.4)	17.1 (16.6-24.2)	17.1 (16.6-21.6)	17.1 (16.5-21.6)	17.5 (16.9-22.0)	17.7 (17.2-23.1)	18.1 (17.5-24.7)	18.1 (17.5-24.0)	18.5 (17.9-26.3)	18.9 (18.2-24.9)	18.9 (18.2-25.5)	18.8 (18.0-26.5)	19.2 (18.4-23.7)	19.9 (19.1-25.9)	21.2 (20.3-29.0)	22.5 (21.3-29.0)	21.8 (20.5-29.2)	22.4 (20.9-29.9)	21.9 (21.2-22.5)	22.2 (21.5-22.9)	22.5 (21.7-23.2)	22.8 (22.0-23.6)	23.1 (22.2-23.9)	23.4 (22.4-24.3)	23.7 (22.7-24.6)	24.0 (22.9-25.0)	24.2 (23.2-25.3)	24.5 (23.4-25.7)	24.8 (23.7-26.0)
**Southern Cone**																																
Argentina	linear	15.9 (13.7-18.4)	17.6 (15.2-20.2)	17.2 (14.8-19.7)	16.4 (14.2-25.3)	14.5 (12.6-16.7)	14.2 (12.3-27.7)	14.5 (12.6-16.6)	13.7 (11.9-22.3)	14.5 (12.6-25.8)	14.0 (12.2-22.7)	14.3 (12.4-26.0)	14.0 (12.2-22.8)	15.0 (13.0-17.3)	14.2 (12.2-16.3)	15.1 (12.9-27.1)	13.5 (11.4-22.4)	13.6 (11.3-16.0)	14.3 (11.8-17.1)	14.9 (12.1-18.1)	13.5 (10.8-16.6)	13.4 (12.5-14.3)	13.2 (12.3-14.2)	13.1 (12.1-14.1)	13.0 (11.9-14.1)	12.9 (11.7-14.0)	12.7 (11.5-14.0)	12.6 (11.3-13.9)	12.5 (11.1-13.8)	12.3 (10.9-13.8)	12.2 (10.7-13.7)	12.1 (10.5-13.7)
Brazil	linear	7.4 (6.8-10.7)	8.1 (7.5-14.2)	7.6 (7.1-12.1)	7.6 (7.1-14.9)	7.6 (7.1-13.8)	8.0 (7.5-12.6)	7.8 (7.3-15.2)	8.1 (7.6-12.7)	8.4 (7.9-15.3)	8.3 (7.8-17.0)	7.8 (7.4-12.5)	8.2 (7.7-14.7)	8.3 (7.8-13.1)	8.6 (8.1-13.3)	8.7 (8.1-15.3)	8.9 (8.3-15.1)	9.1 (8.5-15.1)	9.9 (9.2-17.9)	10.4 (9.5-16.9)	10.3 (9.3-17.8)	9.8 (9.4-10.2)	10.0 (9.5-10.4)	10.1 (9.6-10.6)	10.2 (9.7-10.7)	10.4 (9.8-10.9)	10.5 (9.9-11.1)	10.6 (10.0-11.2)	10.8 (10.1-11.4)	10.9 (10.2-11.5)	11.0 (10.3-11.7)	11.1 (10.4-11.9)
Chile	lowess & ARIMA	19.0 (16.6-21.5)	18.5 (16.3-20.9)	17.0 (15.0-19.1)	17.0 (15.0-33.0)	17.6 (15.6-19.7)	16.3 (14.5-28.6)	16.8 (14.9-32.8)	17.7 (15.8-30.5)	19.4 (17.3-21.6)	19.9 (17.8-22.2)	18.3 (16.3-31.3)	18.4 (16.4-20.6)	16.6 (14.7-18.5)	15.3 (13.5-17.1)	15.3 (13.5-17.3)	14.8 (12.9-27.6)	15.4 (13.3-17.8)	14.1 (11.9-16.6)	13.2 (11.0-15.8)	13.4 (10.9-16.2)	13.0 (12.7-13.3)	12.7 (11.9-13.5)	12.4 (11.0-13.8)	12.1 (10.1-14.1)	11.9 (9.3-14.4)	11.6 (8.6-14.6)	11.3 (7.9-14.6)	11.0 (7.3-14.6)	10.6 (6.7-14.5)	10.3 (6.2-14.4)	10.0 (5.7-14.3)
Paraguay	linear	4.8 (3.0-6.3)	4.6 (2.8-6.1)	4.4 (2.7-5.8)	5.5 (3.3-7.3)	6.4 (3.8-24.5)	6.0 (3.5-8.0)	6.4 (3.8-8.4)	6.0 (3.7-7.8)	5.5 (3.4-7.2)	7.0 (4.4-9.2)	5.4 (3.5-7.1)	6.7 (4.1-8.7)	6.2 (3.8-8.2)	7.4 (4.4-30.8)	8.0 (4.6-25.8)	8.4 (4.8-11.1)	8.3 (4.8-11.3)	8.8 (4.9-12.3)	8.9 (4.9-12.9)	9.0 (4.9-13.4)	9.1 (8.5-9.7)	9.3 (8.7-10.0)	9.6 (8.9-10.3)	9.8 (9.1-10.5)	10.0 (9.3-10.8)	10.3 (9.5-11.1)	10.5 (9.6-11.4)	10.7 (9.8-11.6)	11.0 (10.0-11.9)	11.2 (10.2-12.2)	11.4 (10.4-12.5)
Uruguay	linear	25.7 (22.2-29.4)	21.8 (18.9-25.0)	30.1 (26.1-34.4)	23.2 (20.1-26.5)	23.3 (20.2-26.7)	21.8 (18.9-24.9)	21.9 (18.9-25.0)	25.6 (22.1-53.6)	22.5 (19.5-25.8)	22.7 (19.6-25.9)	23.3 (20.1-26.7)	24.7 (21.3-28.3)	27.1 (23.3-31.1)	23.1 (19.9-26.5)	25.5 (21.9-29.3)	27.7 (23.6-32.1)	30.3 (25.6-35.4)	30.8 (25.6-36.5)	31.5 (25.8-37.9)	31.1 (25.1-38.0)	29.4 (26.7-32.2)	29.8 (26.9-32.7)	30.1 (27.0-33.3)	30.5 (27.2-33.8)	30.9 (27.3-34.4)	31.2 (27.5-35.0)	31.6 (27.6-35.6)	31.9 (27.7-36.1)	32.3 (27.9-36.7)	32.6 (28.0-37.3)	33.0 (28.2-37.8)

Supplementary Table 9. Observed and Forecasted Sex-specific Age-standardized Suicide Mortality Rates and 95% Confidence Intervals Among Females, 2000-2030

Location Name	Model	2000	2001	2002	2003	2004	2005	2006	2007	2008	2009	2010	2011	2012	2013	2014	2015	2016	2017	2018	2019	2020	2021	2022	2023	2024	2025	2026	2027	2028	2029	2030
**Andean Area**																																
Bolivia, Plurinational State of	linear	5.2 (2.9-8.7)	4.9 (2.8-8.4)	5.0 (2.8-8.4)	4.8 (2.7-8.2)	4.8 (2.7-8.2)	4.8 (2.6-8.2)	4.6 (2.5-7.9)	4.8 (2.6-8.3)	4.7 (2.6-8.2)	4.7 (2.5-8.3)	4.7 (2.5-8.2)	4.7 (2.5-8.3)	4.6 (2.4-8.0)	4.6 (2.4-7.9)	4.6 (2.4-8.0)	4.6 (2.4-8.0)	4.7 (2.4-8.2)	4.5 (2.3-7.9)	4.3 (2.2-7.6)	4.2 (2.2-7.3)	4.4 (4.3-4.5)	4.3 (4.2-4.4)	4.3 (4.2-4.4)	4.3 (4.1-4.4)	4.2 (4.1-4.4)	4.2 (4.1-4.3)	4.2 (4.0-4.3)	4.1 (4.0-4.3)	4.1 (3.9-4.3)	4.1 (3.9-4.3)	4.0 (3.9-4.2)
Colombia	lowess & ARIMA	2.6 (2.2-8.9)	2.5 (2.1-2.9)	2.3 (1.9-12.6)	2.5 (2.1-11.2)	2.2 (1.8-8.7)	1.9 (1.6-10.7)	1.9 (1.6-8.2)	1.8 (1.5-2.1)	1.8 (1.5-2.1)	1.7 (1.4-8.2)	1.5 (1.2-12.4)	1.5 (1.2-8.6)	1.5 (1.2-8.3)	1.5 (1.2-8.2)	1.4 (1.1-1.7)	1.8 (1.5-2.2)	1.6 (1.2-2.0)	1.8 (1.4-2.4)	1.7 (1.2-2.3)	1.7 (1.1-2.4)	1.9 (1.8-1.9)	1.9 (1.9-2.0)	2.0 (1.9-2.1)	2.1 (1.8-2.3)	2.1 (1.8-2.4)	2.2 (1.8-2.6)	2.3 (1.7-2.8)	2.3 (1.7-3.0)	2.4 (1.6-3.2)	2.5 (1.5-3.4)	2.5 (1.5-3.6)
Ecuador	ARIMA	4.2 (3.3-5.1)	4.4 (3.5-5.5)	4.9 (3.9-6.0)	5.1 (4.0-6.2)	5.5 (4.4-6.7)	5.6 (4.5-6.8)	5.8 (4.7-7.2)	5.6 (4.5-6.9)	5.5 (4.5-6.8)	5.5 (4.4-6.7)	5.4 (4.4-6.6)	5.2 (4.2-6.3)	5.1 (4.0-6.2)	4.8 (3.8-6.0)	4.5 (3.5-5.5)	4.2 (3.3-5.3)	4.3 (3.3-5.4)	4.2 (3.2-5.4)	3.8 (2.8-5.1)	3.6 (2.5-5.0)	3.3 (2.9-3.7)	3.0 (2.4-3.7)	2.8 (1.7-3.9)	2.5 (1.0-4.0)	2.2 (0.2-4.3)	2.0 (-0.6-4.6)	1.7 (-1.5-4.9)	1.4 (-2.4-5.2)	1.2 (-3.3-5.6)	0.9 (-4.2-6.0)	0.6 (-5.2-6.5)
Peru	linear	2.4 (1.7-3.3)	2.1 (1.5-2.9)	2.1 (1.4-2.8)	2.1 (1.4-2.8)	1.8 (1.3-2.5)	1.8 (1.3-2.5)	1.7 (1.2-2.4)	1.6 (1.1-2.2)	1.6 (1.1-2.2)	1.9 (1.3-2.6)	2.0 (1.4-2.7)	1.9 (1.3-2.6)	1.8 (1.2-2.5)	1.7 (1.1-2.4)	1.6 (1.0-2.3)	1.5 (0.9-2.1)	1.4 (0.9-2.2)	1.4 (0.8-2.2)	1.4 (0.8-2.2)	1.4 (0.8-2.2)	1.3 (1.2-1.5)	1.3 (1.1-1.4)	1.2 (1.1-1.4)	1.2 (1.0-1.4)	1.1 (1.0-1.3)	1.1 (0.9-1.3)	1.1 (0.9-1.3)	1.0 (0.8-1.2)	1.0 (0.8-1.2)	0.9 (0.7-1.2)	0.9 (0.6-1.1)
Venezuela, Bolivarian Republic of	linear	1.6 (1.4-2.0)	1.8 (1.5-2.1)	1.9 (1.6-10.8)	1.8 (1.5-2.2)	1.6 (1.3-9.9)	1.5 (1.2-1.8)	1.2 (1.0-1.5)	1.2 (1.0-1.5)	1.1 (0.9-9.0)	1.1 (0.9-8.5)	1.1 (0.9-1.3)	0.9 (0.7-1.1)	1.0 (0.8-1.2)	0.8 (0.7-1.1)	0.7 (0.5-0.9)	0.8 (0.6-1.1)	0.8 (0.6-1.1)	0.8 (0.5-1.1)	0.7 (0.5-1.0)	0.7 (0.5-1.0)	0.5 (0.3-0.6)	0.4 (0.3-0.5)	0.3 (0.2-0.5)	0.3 (0.1-0.4)	0.2 (0.0-0.4)	0.1 (-0.1-0.3)	0.1 (-0.1-0.3)	-0.0 (-0.2-0.2)	-0.1 (-0.3-0.1)	-0.1 (-0.4-0.1)	-0.2 (-0.4-0.0)
**Central America, Mexico and Latin Caribbean**																																
Costa Rica	linear	1.6 (1.2-2.0)	1.5 (1.1-1.8)	2.0 (1.6-2.5)	2.5 (2.0-3.1)	2.3 (1.8-2.9)	1.9 (1.5-2.4)	2.7 (2.1-3.5)	1.9 (1.5-2.3)	1.6 (1.2-2.0)	1.7 (1.3-2.2)	1.9 (1.5-2.4)	2.3 (1.8-2.8)	1.9 (1.4-2.4)	1.7 (1.3-2.1)	1.8 (1.4-2.3)	2.2 (1.6-2.8)	2.2 (1.7-2.8)	2.2 (1.6-3.0)	2.1 (1.4-3.0)	1.9 (1.3-2.8)	2.1 (1.7-2.4)	2.1 (1.7-2.4)	2.1 (1.7-2.5)	2.1 (1.7-2.5)	2.1 (1.7-2.5)	2.1 (1.7-2.6)	2.1 (1.6-2.6)	2.1 (1.6-2.6)	2.1 (1.6-2.7)	2.1 (1.6-2.7)	2.1 (1.6-2.7)
Cuba	ARIMA	8.9 (7.7-10.3)	7.5 (6.5-8.7)	7.3 (6.2-8.5)	5.9 (5.1-6.9)	5.9 (5.0-6.9)	5.7 (4.8-6.7)	4.2 (3.6-4.9)	4.0 (3.4-4.7)	4.7 (3.9-5.5)	4.9 (4.2-5.8)	4.8 (4.0-5.6)	4.5 (3.8-5.3)	4.2 (3.5-4.9)	4.2 (3.5-5.0)	4.2 (3.5-5.0)	4.0 (3.3-4.9)	3.8 (3.1-4.6)	4.4 (3.5-5.5)	4.1 (3.1-5.3)	4.1 (2.9-5.6)	4.0 (2.8-5.2)	4.0 (2.1-5.9)	3.9 (1.3-6.6)	3.9 (0.5-7.2)	3.9 (-0.2-8.0)	3.8 (-1.1-8.7)	3.8 (-1.9-9.5)	3.7 (-2.8-10.3)	3.7 (-3.8-11.1)	3.6 (-4.7-12.0)	3.6 (-5.7-12.9)
Dominican Republic	linear	1.6 (1.1-2.2)	1.6 (1.1-2.2)	1.8 (1.2-2.6)	1.9 (1.3-2.7)	2.0 (1.4-2.9)	2.0 (1.4-2.9)	1.9 (1.2-2.7)	1.9 (1.2-2.9)	1.9 (1.2-2.9)	1.9 (1.2-2.9)	1.8 (1.2-2.7)	1.9 (1.2-2.8)	2.0 (1.3-2.9)	2.1 (1.4-3.1)	2.3 (1.5-3.4)	2.4 (1.5-3.6)	2.4 (1.5-3.6)	2.2 (1.3-3.4)	2.0 (1.1-3.2)	1.9 (1.1-3.0)	2.2 (2.1-2.4)	2.2 (2.1-2.4)	2.3 (2.1-2.5)	2.3 (2.1-2.5)	2.3 (2.1-2.5)	2.3 (2.1-2.6)	2.4 (2.1-2.6)	2.4 (2.1-2.6)	2.4 (2.1-2.7)	2.4 (2.1-2.7)	2.5 (2.2-2.7)
El Salvador	linear	3.4 (2.8-4.1)	3.8 (3.1-4.5)	3.1 (2.6-3.8)	3.3 (2.7-4.0)	2.7 (2.2-3.2)	2.7 (2.2-3.3)	2.1 (1.7-2.6)	2.5 (2.0-3.0)	2.6 (2.1-3.1)	2.4 (1.9-3.0)	2.1 (1.6-16.7)	2.6 (2.1-3.3)	2.6 (2.0-3.2)	2.3 (1.8-2.9)	2.6 (2.0-3.4)	2.0 (1.5-2.7)	2.2 (1.5-3.1)	2.2 (1.4-3.1)	2.1 (1.4-3.1)	2.1 (1.3-3.1)	1.9 (1.6-2.2)	1.8 (1.5-2.1)	1.8 (1.4-2.1)	1.7 (1.3-2.1)	1.6 (1.2-2.0)	1.6 (1.1-2.0)	1.5 (1.1-1.9)	1.4 (1.0-1.9)	1.4 (0.9-1.8)	1.3 (0.8-1.8)	1.2 (0.7-1.8)
Guatemala	linear	4.1 (3.1-5.3)	4.3 (3.3-5.6)	4.5 (3.4-5.9)	4.5 (3.4-5.8)	4.5 (3.4-5.8)	4.5 (3.4-5.9)	4.3 (3.3-5.6)	3.9 (2.9-5.0)	3.7 (2.8-4.7)	3.7 (2.8-4.8)	3.4 (2.6-4.4)	3.1 (2.4-4.0)	3.0 (2.3-3.8)	2.9 (2.2-3.8)	2.9 (2.2-3.6)	2.9 (2.2-3.6)	2.8 (2.1-3.6)	2.6 (1.9-3.5)	2.6 (1.8-3.5)	2.5 (1.7-3.5)	2.3 (2.0-2.5)	2.2 (1.9-2.4)	2.0 (1.8-2.3)	1.9 (1.6-2.2)	1.8 (1.5-2.1)	1.7 (1.4-2.0)	1.6 (1.2-1.9)	1.4 (1.1-1.8)	1.3 (0.9-1.7)	1.2 (0.8-1.6)	1.1 (0.7-1.5)
Haiti	linear	11.1 (6.3-17.9)	10.8 (6.1-17.4)	10.6 (6.0-17.1)	10.5 (5.9-16.9)	10.0 (5.6-16.0)	10.5 (5.9-16.9)	10.5 (5.9-16.9)	10.7 (6.0-17.5)	10.5 (5.9-17.3)	10.6 (5.8-17.5)	10.8 (5.9-18.0)	10.1 (5.5-16.6)	9.6 (5.3-15.8)	9.4 (5.2-15.3)	9.3 (5.1-15.2)	9.1 (5.0-15.0)	8.7 (4.8-14.4)	8.3 (4.6-13.7)	8.2 (4.5-13.5)	8.0 (4.4-13.1)	8.3 (7.9-8.7)	8.2 (7.7-8.6)	8.0 (7.5-8.5)	7.9 (7.3-8.4)	7.7 (7.2-8.3)	7.6 (7.0-8.2)	7.4 (6.8-8.0)	7.3 (6.6-7.9)	7.1 (6.4-7.8)	7.0 (6.2-7.7)	6.8 (6.1-7.6)
Honduras	linear	1.1 (0.6-1.9)	1.1 (0.6-1.9)	1.1 (0.6-1.9)	1.1 (0.6-1.9)	1.2 (0.7-2.0)	1.2 (0.6-2.0)	1.2 (0.6-2.0)	1.1 (0.6-1.9)	1.1 (0.6-1.9)	1.0 (0.6-1.7)	1.0 (0.6-1.8)	1.0 (0.6-1.7)	1.0 (0.6-1.7)	1.0 (0.6-1.8)	1.0 (0.6-1.8)	1.1 (0.6-1.8)	1.0 (0.6-1.7)	1.0 (0.6-1.8)	1.0 (0.5-1.7)	1.0 (0.5-1.7)	1.0 (0.9-1.0)	1.0 (0.9-1.0)	0.9 (0.9-1.0)	0.9 (0.9-1.0)	0.9 (0.9-1.0)	0.9 (0.9-1.0)	0.9 (0.9-1.0)	0.9 (0.8-0.9)	0.9 (0.8-0.9)	0.9 (0.8-0.9)	0.9 (0.8-0.9)
Mexico	linear	1.1 (1.1-5.1)	1.4 (1.3-5.4)	1.3 (1.3-5.3)	1.4 (1.3-1.5)	1.4 (1.3-1.4)	1.4 (1.3-1.5)	1.3 (1.2-1.4)	1.6 (1.4-1.7)	1.7 (1.6-1.8)	1.8 (1.7-1.9)	1.6 (1.5-1.8)	2.0 (1.9-2.1)	1.9 (1.8-2.0)	2.0 (1.8-2.1)	2.2 (2.1-2.4)	2.3 (2.1-2.4)	2.1 (1.9-2.2)	2.1 (2.0-2.3)	2.2 (1.8-2.5)	2.2 (1.7-2.7)	2.4 (2.3-2.5)	2.4 (2.3-2.6)	2.5 (2.4-2.6)	2.5 (2.4-2.7)	2.6 (2.5-2.8)	2.7 (2.5-2.8)	2.7 (2.6-2.9)	2.8 (2.6-3.0)	2.8 (2.7-3.0)	2.9 (2.7-3.1)	3.0 (2.8-3.2)
Nicaragua	linear	3.6 (2.7-4.5)	3.3 (2.6-4.2)	2.8 (2.2-3.6)	3.1 (2.4-4.0)	2.7 (2.1-3.4)	2.1 (1.7-2.6)	2.0 (1.6-2.6)	2.4 (1.9-3.1)	2.6 (2.0-3.4)	2.8 (2.2-3.6)	2.4 (1.8-3.0)	2.9 (2.1-25.5)	2.5 (1.9-3.3)	2.4 (1.8-3.2)	2.2 (1.6-2.8)	2.2 (1.6-2.9)	1.7 (1.3-2.3)	2.1 (1.5-2.9)	2.2 (1.5-3.1)	1.9 (1.2-2.8)	1.9 (1.5-2.2)	1.8 (1.5-2.2)	1.7 (1.4-2.1)	1.7 (1.3-2.1)	1.6 (1.2-2.0)	1.6 (1.1-2.0)	1.5 (1.0-2.0)	1.4 (1.0-1.9)	1.4 (0.9-1.9)	1.3 (0.8-1.9)	1.3 (0.7-1.8)
Panama	linear	1.5 (1.1-1.9)	1.7 (1.3-2.2)	2.3 (1.8-3.0)	1.3 (1.0-1.7)	2.0 (1.6-2.6)	1.6 (1.2-2.0)	0.8 (0.6-1.0)	1.2 (0.9-1.6)	2.0 (1.5-2.5)	1.5 (1.1-1.9)	1.1 (0.8-1.4)	1.2 (0.9-1.5)	0.9 (0.7-1.2)	0.7 (0.5-0.9)	1.1 (0.8-1.5)	1.1 (0.8-1.4)	1.0 (0.8-1.4)	0.9 (0.7-1.3)	1.0 (0.7-1.4)	1.0 (0.6-1.4)	0.8 (0.4-1.1)	0.7 (0.4-1.1)	0.7 (0.3-1.1)	0.6 (0.2-1.0)	0.6 (0.1-1.0)	0.5 (0.1-1.0)	0.5 (-0.0-1.0)	0.4 (-0.1-0.9)	0.4 (-0.2-0.9)	0.3 (-0.2-0.9)	0.3 (-0.3-0.9)
**Non-Latin Caribbean**																																
Antigua and Barbuda	lowess & ARIMA	0.0 (0.0-0.0)	0.0 (0.0-0.0)	0.0 (0.0-0.0)	0.0 (0.0-0.0)	0.0 (0.0-0.0)	0.0 (0.0-0.0)	0.0 (0.0-0.0)	0.0 (0.0-0.0)	0.0 (0.0-0.0)	0.0 (0.0-0.0)	0.0 (0.0-0.0)	0.2 (0.1-0.2)	0.0 (0.0-0.0)	0.0 (0.0-0.0)	0.8 (0.6-1.1)	0.8 (0.6-1.1)	0.9 (0.7-1.3)	0.0 (0.0-0.0)	0.6 (0.4-0.9)	0.6 (0.4-0.8)	0.5 (0.5-0.6)	0.5 (0.4-0.6)	0.5 (0.3-0.6)	0.4 (0.2-0.7)	0.4 (0.1-0.7)	0.4 (0.0-0.8)	0.4 (-0.0-0.9)	0.4 (-0.1-0.9)	0.4 (-0.2-1.0)	0.4 (-0.2-1.0)	0.4 (-0.2-1.1)
Bahamas	lowess & ARIMA	1.1 (0.8-1.5)	1.1 (0.8-1.4)	0.8 (0.6-1.1)	0.9 (0.7-1.3)	1.3 (0.9-1.8)	1.3 (0.9-1.7)	1.4 (1.0-1.9)	1.3 (0.9-1.7)	1.2 (0.9-1.7)	1.2 (0.9-1.7)	1.2 (0.9-1.7)	1.3 (0.9-1.7)	1.2 (0.8-1.6)	1.1 (0.8-1.4)	1.0 (0.7-1.4)	1.1 (0.8-1.5)	1.1 (0.7-1.5)	1.0 (0.7-1.4)	1.2 (0.8-1.7)	1.2 (0.8-1.8)	1.3 (1.2-1.3)	1.3 (1.2-1.3)	1.3 (1.2-1.4)	1.3 (1.2-1.4)	1.3 (1.2-1.4)	1.3 (1.1-1.4)	1.2 (1.1-1.3)	1.2 (1.0-1.3)	1.1 (1.0-1.2)	1.1 (0.9-1.2)	1.0 (0.9-1.2)
Barbados	linear	0.5 (0.3-0.6)	0.3 (0.2-0.4)	0.2 (0.1-0.2)	0.9 (0.7-1.1)	1.0 (0.8-1.4)	0.8 (0.6-1.1)	0.2 (0.1-0.2)	0.1 (0.1-0.2)	0.2 (0.2-0.3)	0.2 (0.2-0.3)	0.3 (0.2-0.4)	0.2 (0.2-0.3)	0.2 (0.2-0.3)	0.0 (0.0-0.0)	0.2 (0.1-0.2)	0.2 (0.1-0.2)	0.2 (0.1-0.2)	0.2 (0.1-0.2)	0.2 (0.1-0.2)	0.2 (0.1-0.2)	0.0 (-0.2-0.3)	0.0 (-0.2-0.3)	-0.0 (-0.3-0.3)	-0.0 (-0.3-0.3)	-0.1 (-0.4-0.2)	-0.1 (-0.4-0.2)	-0.1 (-0.5-0.2)	-0.1 (-0.5-0.2)	-0.2 (-0.5-0.2)	-0.2 (-0.6-0.2)	-0.2 (-0.6-0.2)
Belize	linear	2.9 (2.2-3.9)	2.5 (1.9-3.3)	2.6 (1.9-3.4)	2.4 (1.8-3.2)	2.5 (1.8-3.3)	2.0 (1.5-2.7)	2.1 (1.5-2.7)	1.9 (1.4-2.5)	1.7 (1.3-2.3)	2.0 (1.5-2.6)	2.1 (1.6-2.8)	2.1 (1.5-2.7)	1.9 (1.4-2.5)	1.8 (1.3-2.4)	1.7 (1.3-2.3)	1.6 (1.2-2.1)	1.5 (1.1-2.1)	1.7 (1.2-2.2)	1.6 (1.2-2.3)	1.8 (1.3-2.5)	1.4 (1.3-1.6)	1.4 (1.2-1.6)	1.3 (1.1-1.5)	1.3 (1.1-1.5)	1.2 (1.0-1.5)	1.2 (0.9-1.4)	1.1 (0.8-1.4)	1.1 (0.8-1.3)	1.0 (0.7-1.3)	0.9 (0.6-1.3)	0.9 (0.6-1.2)
Grenada	linear	0.6 (0.4-0.8)	0.8 (0.6-1.0)	0.5 (0.4-0.7)	0.5 (0.3-0.6)	0.9 (0.7-1.2)	0.8 (0.6-1.1)	1.6 (1.2-2.2)	0.6 (0.4-0.8)	0.7 (0.5-0.9)	0.0 (0.0-0.0)	0.0 (0.0-0.0)	0.0 (0.0-0.0)	0.5 (0.4-0.7)	0.5 (0.4-0.7)	0.8 (0.6-1.1)	0.3 (0.2-0.3)	0.9 (0.6-1.2)	0.6 (0.4-0.8)	0.7 (0.5-1.0)	0.7 (0.5-1.1)	0.5 (0.1-0.9)	0.5 (0.1-0.9)	0.5 (0.1-0.9)	0.5 (0.0-1.0)	0.5 (0.0-1.0)	0.5 (-0.0-1.0)	0.5 (-0.1-1.0)	0.5 (-0.1-1.0)	0.5 (-0.1-1.1)	0.5 (-0.2-1.1)	0.5 (-0.2-1.1)
Guyana	linear	14.4 (10.1-39.9)	15.5 (10.9-51.0)	14.6 (10.3-45.6)	16.8 (12.0-46.2)	16.0 (11.5-21.9)	15.1 (10.7-20.7)	15.8 (11.3-21.4)	15.8 (11.3-21.5)	15.3 (10.9-20.9)	19.5 (13.9-26.5)	15.8 (11.3-21.6)	14.8 (10.5-20.3)	15.9 (11.1-21.8)	17.1 (11.9-23.8)	13.6 (9.3-18.8)	15.8 (10.7-22.5)	16.1 (10.6-23.2)	16.3 (10.6-24.0)	16.7 (10.6-25.0)	16.9 (10.6-25.9)	16.5 (15.3-17.7)	16.5 (15.3-17.8)	16.6 (15.2-18.0)	16.6 (15.2-18.1)	16.7 (15.1-18.2)	16.7 (15.1-18.4)	16.8 (15.1-18.5)	16.9 (15.0-18.7)	16.9 (15.0-18.8)	17.0 (15.0-19.0)	17.0 (14.9-19.1)
Jamaica	linear	0.9 (0.7-1.2)	0.9 (0.7-1.2)	0.8 (0.6-1.1)	0.9 (0.6-1.1)	0.8 (0.6-1.0)	0.7 (0.5-1.0)	0.7 (0.5-1.0)	0.8 (0.6-1.0)	0.8 (0.6-1.1)	0.9 (0.6-1.1)	0.9 (0.6-1.1)	0.9 (0.7-1.2)	0.9 (0.7-1.2)	0.9 (0.7-1.2)	1.0 (0.7-1.3)	1.0 (0.7-1.4)	1.0 (0.7-1.4)	1.0 (0.7-1.4)	1.0 (0.7-1.4)	1.0 (0.7-1.5)	1.0 (0.9-1.1)	1.0 (1.0-1.1)	1.0 (1.0-1.1)	1.0 (1.0-1.1)	1.1 (1.0-1.1)	1.1 (1.0-1.1)	1.1 (1.0-1.2)	1.1 (1.0-1.2)	1.1 (1.0-1.2)	1.1 (1.0-1.2)	1.1 (1.0-1.2)
Saint Lucia	linear	2.0 (1.5-2.7)	1.9 (1.4-2.6)	1.8 (1.3-2.3)	1.7 (1.2-2.2)	1.6 (1.2-2.1)	1.6 (1.2-2.2)	1.6 (1.2-2.2)	1.6 (1.2-2.1)	1.7 (1.2-2.2)	1.5 (1.1-2.0)	1.6 (1.2-2.2)	1.5 (1.1-2.0)	1.7 (1.2-2.3)	1.6 (1.2-2.2)	1.6 (1.2-2.2)	1.6 (1.2-2.2)	1.5 (1.1-2.0)	1.5 (1.1-2.1)	1.5 (1.1-2.1)	1.5 (1.0-2.1)	1.4 (1.3-1.5)	1.4 (1.3-1.5)	1.4 (1.3-1.5)	1.4 (1.3-1.5)	1.4 (1.2-1.5)	1.3 (1.2-1.5)	1.3 (1.2-1.5)	1.3 (1.2-1.4)	1.3 (1.1-1.4)	1.3 (1.1-1.4)	1.2 (1.1-1.4)
Saint Vincent and the Grenadines	linear	0.5 (0.4-0.7)	1.3 (0.9-1.7)	1.5 (1.1-2.0)	1.3 (0.9-1.7)	1.4 (1.1-1.9)	2.4 (1.8-3.1)	1.6 (1.2-2.0)	1.1 (0.8-1.4)	0.5 (0.4-0.7)	0.6 (0.5-0.8)	0.5 (0.4-0.6)	1.9 (1.4-2.5)	3.9 (2.9-5.2)	1.0 (0.8-1.3)	0.5 (0.3-0.8)	0.9 (0.6-1.2)	0.8 (0.5-1.1)	0.0 (0.0-0.0)	0.7 (0.4-1.0)	0.7 (0.4-1.0)	0.8 (-0.0-1.6)	0.8 (-0.1-1.7)	0.7 (-0.2-1.7)	0.7 (-0.3-1.7)	0.7 (-0.4-1.8)	0.6 (-0.5-1.8)	0.6 (-0.6-1.8)	0.6 (-0.7-1.9)	0.5 (-0.8-1.9)	0.5 (-0.9-1.9)	0.5 (-1.0-2.0)
Suriname	linear	11.8 (8.3-16.1)	12.6 (8.8-17.4)	12.2 (8.6-16.6)	11.8 (8.4-16.0)	11.7 (8.3-15.8)	11.7 (8.4-15.8)	11.8 (8.4-15.8)	11.9 (8.6-16.1)	12.1 (8.7-16.2)	12.1 (8.7-16.2)	12.1 (8.7-16.3)	11.6 (8.3-15.5)	11.6 (8.3-15.7)	11.8 (8.3-16.0)	11.7 (8.2-16.1)	11.8 (8.1-16.4)	11.8 (7.9-16.7)	11.7 (7.7-16.8)	11.9 (7.7-17.3)	11.8 (7.5-17.4)	11.7 (11.5-11.9)	11.7 (11.4-11.9)	11.7 (11.4-11.9)	11.7 (11.4-11.9)	11.6 (11.3-11.9)	11.6 (11.3-11.9)	11.6 (11.3-11.9)	11.6 (11.2-11.9)	11.6 (11.2-11.9)	11.6 (11.2-11.9)	11.5 (11.1-11.9)
Trinidad and Tobago	ARIMA	6.3 (4.9-8.0)	6.3 (4.9-8.0)	5.3 (4.1-6.7)	4.9 (3.8-6.2)	4.5 (3.4-5.7)	4.6 (3.5-5.8)	4.2 (3.2-5.4)	3.6 (2.8-4.7)	4.6 (3.5-5.9)	4.0 (3.0-5.2)	4.5 (3.4-5.8)	3.9 (3.0-5.0)	4.7 (3.5-6.1)	4.5 (3.2-6.0)	4.3 (2.9-6.1)	4.3 (2.8-6.3)	4.2 (2.6-6.3)	4.1 (2.5-6.1)	3.8 (2.3-5.6)	3.6 (2.3-5.5)	3.5 (2.6-4.4)	3.4 (2.3-4.4)	3.2 (2.0-4.4)	3.1 (1.7-4.4)	2.9 (1.4-4.4)	2.8 (1.2-4.4)	2.6 (0.9-4.3)	2.5 (0.7-4.3)	2.3 (0.4-4.3)	2.2 (0.2-4.2)	2.1 (-0.1-4.2)
**North America**																																
Canada	lowess & ARIMA	4.9 (4.3-5.6)	5.0 (4.4-5.7)	4.9 (4.3-5.6)	5.2 (4.5-5.9)	5.2 (4.5-5.9)	5.3 (4.6-6.0)	4.7 (4.1-5.4)	5.0 (4.4-5.7)	5.1 (4.5-5.8)	5.0 (4.4-5.7)	5.5 (4.8-6.2)	5.2 (4.5-15.2)	5.3 (4.6-6.0)	5.5 (4.7-6.2)	5.9 (5.0-6.8)	6.0 (5.0-7.0)	5.5 (4.5-16.2)	5.8 (4.7-6.9)	5.5 (4.4-11.5)	5.4 (4.2-11.3)	5.5 (5.3-5.6)	5.5 (5.3-5.6)	5.5 (5.3-5.7)	5.5 (5.3-5.8)	5.5 (5.3-5.8)	5.6 (5.3-5.9)	5.6 (5.3-5.9)	5.6 (5.3-6.0)	5.6 (5.3-6.0)	5.7 (5.3-6.0)	5.7 (5.3-6.1)
United States	linear	4.0 (3.8-6.8)	4.1 (3.9-4.2)	4.3 (4.1-7.2)	4.3 (4.2-4.5)	4.7 (4.5-4.9)	4.6 (4.4-4.8)	4.7 (4.5-7.7)	4.9 (4.7-7.9)	5.0 (4.8-9.1)	5.0 (4.8-9.1)	5.2 (5.0-5.4)	5.4 (5.1-8.3)	5.4 (5.2-5.7)	5.5 (5.3-9.6)	5.8 (5.5-8.7)	6.0 (5.8-6.3)	6.3 (5.9-6.6)	6.5 (6.1-9.6)	6.5 (6.1-8.6)	6.8 (6.3-8.8)	6.7 (6.6-6.9)	6.9 (6.7-7.0)	7.0 (6.9-7.2)	7.2 (7.0-7.3)	7.3 (7.1-7.5)	7.4 (7.3-7.6)	7.6 (7.4-7.8)	7.7 (7.5-7.9)	7.9 (7.7-8.1)	8.0 (7.8-8.2)	8.1 (7.9-8.4)

**Southern Cone**																																
Argentina	lowess & ARIMA	3.4 (2.9-4.0)	4.0 (3.4-4.7)	4.2 (3.5-4.9)	3.8 (3.2-4.4)	3.5 (3.0-4.1)	3.5 (3.0-4.1)	3.3 (2.8-13.4)	3.2 (2.7-3.8)	3.3 (2.8-10.6)	3.2 (2.7-3.8)	3.1 (2.6-3.6)	3.3 (2.8-3.9)	3.4 (2.9-4.0)	3.1 (2.6-3.7)	3.7 (3.1-4.4)	3.2 (2.7-3.9)	3.1 (2.5-3.7)	3.5 (2.9-4.3)	3.6 (2.8-4.4)	3.3 (2.6-4.2)	3.4 (3.4-3.4)	3.4 (3.3-3.5)	3.4 (3.3-3.6)	3.4 (3.2-3.7)	3.4 (3.1-3.8)	3.4 (2.9-3.9)	3.4 (2.8-4.1)	3.4 (2.7-4.2)	3.5 (2.5-4.4)	3.5 (2.4-4.5)	3.5 (2.2-4.7)
Brazil	linear	1.8 (1.7-2.0)	2.0 (1.8-2.2)	2.2 (2.0-2.3)	2.0 (1.8-2.2)	2.1 (1.9-2.3)	2.2 (1.9-2.4)	2.2 (2.0-6.6)	2.3 (2.1-2.5)	2.4 (2.2-7.8)	2.3 (2.1-2.5)	2.4 (2.2-5.8)	2.4 (2.2-5.7)	2.5 (2.3-5.9)	2.5 (2.3-5.8)	2.4 (2.2-2.6)	2.6 (2.4-2.8)	2.5 (2.3-5.9)	2.8 (2.5-7.5)	2.9 (2.6-6.4)	2.8 (2.5-6.7)	2.8 (2.7-2.9)	2.9 (2.8-2.9)	2.9 (2.8-3.0)	2.9 (2.8-3.0)	3.0 (2.9-3.1)	3.0 (2.9-3.1)	3.1 (3.0-3.2)	3.1 (3.0-3.2)	3.2 (3.0-3.3)	3.2 (3.1-3.3)	3.3 (3.1-3.4)
Chile	ARIMA	2.9 (2.4-3.4)	2.9 (2.4-3.4)	2.9 (2.5-13.6)	2.9 (2.5-3.4)	3.0 (2.6-3.5)	3.1 (2.7-3.7)	3.2 (2.8-3.7)	4.0 (3.4-15.6)	4.6 (3.9-5.3)	4.8 (4.1-5.5)	4.2 (3.6-4.9)	4.0 (3.4-4.6)	3.6 (3.0-4.1)	3.4 (2.9-4.0)	3.3 (2.8-3.9)	3.9 (3.2-4.6)	3.3 (2.7-3.9)	3.0 (2.4-3.6)	3.0 (2.4-3.6)	3.0 (2.3-3.7)	3.0 (2.4-3.7)	3.1 (2.2-4.0)	3.1 (2.1-4.1)	3.2 (2.1-4.2)	3.2 (2.1-4.3)	3.2 (2.1-4.3)	3.2 (2.1-4.4)	3.2 (2.1-4.4)	3.3 (2.1-4.4)	3.3 (2.1-4.4)	3.3 (2.1-4.4)
Paraguay	linear	2.5 (1.8-3.3)	2.5 (1.8-3.3)	3.1 (2.3-4.1)	2.0 (1.5-2.6)	2.9 (2.1-3.8)	3.2 (2.4-4.3)	2.8 (2.0-3.7)	2.4 (1.7-21.3)	2.4 (1.7-3.2)	2.5 (1.8-3.2)	2.2 (1.6-2.9)	2.6 (2.0-3.5)	3.1 (2.3-4.1)	2.7 (2.0-3.6)	3.8 (2.7-22.2)	3.2 (2.3-4.4)	2.9 (2.0-4.1)	3.4 (2.2-4.8)	3.2 (2.0-4.8)	3.3 (2.0-5.0)	3.3 (2.9-3.7)	3.3 (2.9-3.7)	3.4 (2.9-3.8)	3.4 (2.9-3.9)	3.4 (2.9-4.0)	3.5 (3.0-4.0)	3.5 (3.0-4.1)	3.6 (3.0-4.2)	3.6 (3.0-4.2)	3.7 (3.0-4.3)	3.7 (3.0-4.4)
Uruguay	linear	5.1 (4.1-6.2)	5.9 (4.8-7.2)	7.4 (6.0-8.9)	6.5 (5.3-7.9)	5.8 (4.7-29.9)	6.0 (4.8-7.2)	6.9 (5.6-8.3)	7.2 (5.9-8.7)	5.6 (4.5-6.8)	5.3 (4.3-6.4)	6.3 (5.1-7.7)	6.5 (5.2-7.9)	6.3 (5.1-7.6)	6.2 (5.0-7.6)	7.0 (5.6-8.5)	6.7 (5.3-8.2)	8.3 (6.6-10.2)	7.5 (5.8-9.2)	7.4 (5.7-9.2)	7.7 (5.9-9.7)	7.5 (6.8-8.1)	7.5 (6.8-8.3)	7.6 (6.8-8.4)	7.7 (6.9-8.6)	7.8 (6.9-8.7)	7.9 (6.9-8.8)	8.0 (7.0-9.0)	8.1 (7.0-9.1)	8.1 (7.0-9.2)	8.2 (7.1-9.4)	8.3 (7.1-9.5)

Supplementary Table 10. Model fit statistics for the forecasting models

Country	Sex	ARIMA		Linear regression		LOWESS smoothing	
		AIC	BIC	AIC	BIC	AIC	BIC
**Andean Area**							
Bolivia, Plurinational State	Females	-25.581	-23.692	-28.599	-25.612	n/a	n/a
of	Males	6.210	7.991	37.220	40.207	n/a	n/a
Colombia	Females	-9.909	-8.964	3.100	6.087	-106.716	-104.045
	Males	23.335	24.280	31.108	34.096	n/a	n/a
Ecuador	Females	-3.948	-2.167	39.527	42.515	-94.436	-92.655
	Males	58.720	62.702	84.884	87.871	47.426	50.413
Peru	Females	-24.210	-22.321	-17.604	-14.616	n/a	n/a
	Males	0.176	4.159	25.567	28.554	-50.681	-46.699
Venezuela, Bolivarian	Females	-27.741	-25.852	-19.448	-16.461	n/a	n/a
Republic of	Males	32.603	34.492	46.777	49.764	n/a	n/a
**Central America, Mexico and Latin Caribbean**							
Costa Rica	Females	15.038	17.029	16.643	19.630	n/a	n/a
	Males	81.394	83.386	82.208	85.195	-21.016	-16.037
Cuba	Females	37.472	39.253	54.353	57.340	-53.773	-51.992
	Males	55.010	55.955	67.261	70.249	n/a	n/a
Dominican Republic	Females	-27.349	-25.460	-10.413	-7.426	-81.553	-78.720
	Males	28.704	30.593	51.716	54.703	-24.713	-22.932
El Salvador	Females	12.975	15.809	12.939	15.927	n/a	n/a
	Males	85.614	87.605	87.152	90.139	-27.090	-22.112
Guatemala	Females	-15.100	-13.212	4.401	7.388	n/a	n/a
	Males	70.982	72.871	91.033	94.020	n/a	n/a
Haiti	Females	9.591	11.480	27.737	30.725	n/a	n/a
	Males	14.918	15.862	33.932	36.919	-48.249	-45.415
Honduras	Females	-81.681	-80.736	-72.228	-69.240	n/a	n/a
	Males	61.030	64.017	66.877	69.864	-16.958	-11.980
Mexico	Females	-18.554	-15.721	-24.419	-21.432	n/a	n/a
	Males	29.089	30.033	25.731	28.718	n/a	n/a
Nicaragua	Females	15.728	16.672	16.063	19.050	n/a	n/a
	Males	57.187	58.131	50.417	53.404	n/a	n/a
Panama	Females	28.461	29.406	18.235	21.223	n/a	n/a
	Males	53.862	57.640	52.032	55.019	n/a	n/a
**Non-Latin Caribbean**							
Antigua and Barbuda	Females	13.193	14.137	6.602	9.589	-95.094	-92.261
	Males	38.509	40.398	56.853	59.840	n/a	n/a
Bahamas	Females	-23.881	-20.894	-16.099	-13.111	-106.278	-102.295
	Males	27.149	28.093	33.965	36.952	n/a	n/a
Barbados	Females	1.777	4.764	3.442	6.429	n/a	n/a
	Males	60.003	60.948	63.049	66.036	n/a	n/a
Belize	Females	-8.779	-7.835	-5.877	-2.890	n/a	n/a
	Males	76.326	79.313	76.779	79.766	n/a	n/a
Grenada	Females	20.337	22.329	22.105	25.092	-58.694	-54.711
	Males	82.804	85.637	93.536	96.523	n/a	n/a
Guyana	Females	67.959	69.950	68.517	71.505	n/a	n/a
	Males	101.964	105.947	118.328	121.316	19.858	23.636
Jamaica	Females	-67.491	-66.547	-50.383	-47.396	n/a	n/a
	Males	16.070	19.057	18.199	21.186	-57.328	-54.494
Saint Lucia	Females	-35.496	-34.552	-33.796	-30.808	n/a	n/a
	Males	55.233	57.225	54.520	57.507	-46.794	-43.016
Saint Vincent and the	Females	52.409	55.396	54.776	57.763	n/a	n/a
Grenadines	Males	102.452	104.341	100.846	103.833	n/a	n/a
Suriname	Females	1.801	4.788	2.396	5.384	n/a	n/a
	Males	71.895	73.887	70.773	73.761	n/a	n/a
Trinidad and Tobago	Females	27.561	30.394	35.405	38.392	-72.758	-70.977
	Males	79.399	81.288	83.472	86.459	n/a	n/a
**North America**							
Canada	Females	6.948	7.893	1.359	4.346	-49.521	-47.632
	Males	55.227	57.218	56.841	59.828	-45.273	-41.290
United States	Females	-27.863	-25.029	-21.695	-18.708	n/a	n/a
	Males	29.401	31.289	44.871	47.858	n/a	n/a
**Southern Cone**							
Argentina	Females	7.148	10.135	8.655	11.642	-94.636	-92.855
	Males	53.340	54.285	56.937	59.924	n/a	n/a
Brazil	Females	-26.368	-24.479	-38.465	-35.478	n/a	n/a
	Males	14.849	16.738	25.735	28.722	n/a	n/a
Chile	Females	19.972	22.959	40.722	43.709	-54.232	-49.253

	Males	56.801	57.745	73.464	76.452	-10.071	-6.293
Paraguay	Females	32.560	33.505	23.873	26.861	n/a	n/a
	Males	42.630	45.463	40.359	43.346	n/a	n/a
Uruguay	Females	48.924	49.869	46.556	49.543	n/a	n/a
	Males	96.323	98.212	101.717	104.704	n/a	n/a

n/a: not applicable

Supplementary Table 11. Proportion of firearm- and pesticide-involved suicides by country and sex in the Region of Americas

Country	Year	Proportion of firearm-involved suicides (%)		Proportion of pesticide-involved suicides (%)	
		Females	Males	Females	Males
Argentina	2019	5.23	15.19	0.17	0.19
Bahamas	n/a	n/a	n/a	n/a	n/a
Belize	2016	0	7.69	12.50	15.38
Bolivia	n/a	n/a	n/a	n/a	n/a
Brazil	2019	3.43	8.34	3.05	2.00
Canada	2019	1.68	16.94	0	0
Chile	2019	1.93	3.70	0	0.52
Colombia	2019	2.21	11.37	8.52	5.90
Costa Rica	2019	3.77	14.24	13.21	10.61
Cuba	2019	0.39	1.33	2.32	0.75
Dominican Republic	2018	2.21	4.52	2.21	2.09
Ecuador	2019	0	4.64	8.13	4.85
El Salvador	2018	0	3.76	58.70	40.32
Guatemala	2019	1.27	7.45	22.29	14.91
Guyana	2019	0	2.17	73.17	50.00
Haiti	n/a	n/a	n/a	n/a	n/a
Mexico	2019	3.39	7.45	5.32	2.21
Nicaragua	2019	0	2.23	47.37	29.00
Paraguay	2019	4.92	13.21	0.82	1.35
Saint Lucia	n/a	n/a	n/a	n/a	n/a
Suriname	2014	2.78	7.61	75.00	67.39
Trinidad and Tobago	2012	0	0.89	18.92	20.54
United States of America	2019	31.39	55.66	0	0
Uruguay	2019	12.75	22.47	0.67	0.52

Note. The following countries had a suicide mortality rate less than three per 100,000 population in 2019, and therefore were not included in the counterfactual scenario estimates: Antigua and Barbuda, Barbados, Grenada, Honduras, Jamaica, Panama, Peru, Saint Vincent and the Grenadines, and Venezuela.
n/a: Not available

Supplementary Table 12. Search strategy employed in PubMed, Embase, and PsycInfo (OVID)

	**Searches**
1	policy/ or public policy/ or health policy/
2	Pesticides/ and 1
3	(pesticide* adj6 ban).tw,kw,kf.
4	2 or 3
5	Firearms/ and 1
6	((firearm* or gun or guns) adj6 (policy or policies or regulate or regulation or restrict or restriction or ban or control)).tw,kw,kf.
7	5 or 6
8	4 or 7
9	exp Suicide Prevention/ or exp Suicide/
10	(suicide adj3 (prevent* or mortality or death)).tw,kw,kf.
11	9 or 10
12	8 and 11
13	remove duplicates from 12

Supplementary Table 13. PICOS criteria for study selection

Criteria	Inclusion criteria	Exclusion criteria
Population	Population level (e.g., national coverage) or clearly defined sub-population (e.g., distinct age-group); all ages; any country	None
Intervention or exposure	Any suicide means restriction intervention or policy implemented on a national-level	Means restriction isolated to one site or setting within a country
Comparator	Comparison of before and after means restriction implementation	None
Outcome	Suicide mortality rate	None
Study design	Quantitative observational pre/post study design	None
Other	Any language, any geographical region, and any year of publication	None

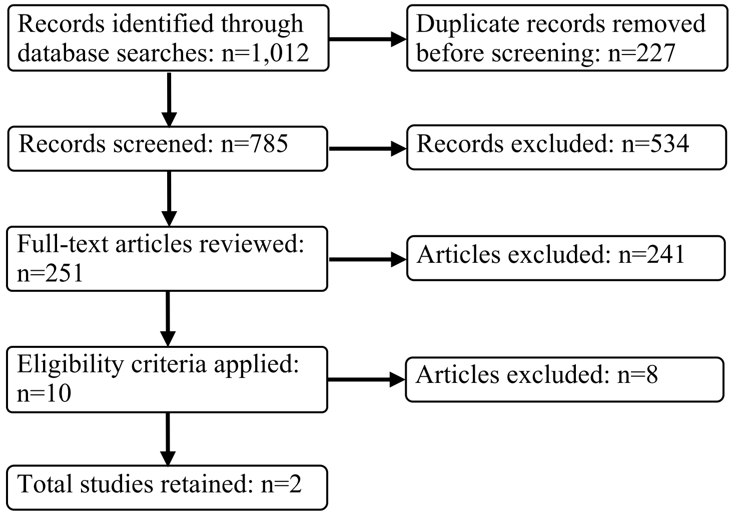

Supplementary Figure 13. PRISMA flow diagram of study selection

Supplementary Table 14. Effect estimates for a firearm ban

				Firearm-involved suicides			Other suicide means		
Reference	Country	Means restriction	Year	Rate ratio	Lower 95% CI	Upper 95% CI	Rate ratio	Lower 95% CI	Upper 95% CI
Chapman et al., 2016	Australia	In 1996, a ban on semiautomatic rifles and pump-action shotguns and rifles and also initiated a program for buyback of firearms was implemented	1979-1996 (mean)	-	-	-	-	-	-
			1997	0.53	0.47	0.59	1.38	1.32	1.44
			1998	0.44	0.39	0.50	1.39	1.33	1.45
			1999	0.46	0.41	0.52	1.28	1.22	1.33
			2000	0.39	0.34	0.44	1.24	1.18	1.29
			2001	0.45	0.40	0.51	1.24	1.18	1.29
			2002	0.36	0.32	0.42	1.17	1.12	1.22
			2003	0.31	0.27	0.36	1.08	1.04	1.13
			2004	0.28	0.24	0.33	1.06	1.01	1.11
			2005	0.24	0.20	0.28	1.05	1.00	1.10
			2006	0.31	0.27	0.36	1.03	0.99	1.08
			2007	0.30	0.26	0.34	1.08	1.03	1.13
			2008	0.29	0.25	0.33	1.09	1.05	1.14
			2009	0.25	0.22	0.29	1.10	1.05	1.15
			2010	0.26	0.22	0.30	1.10	1.05	1.15
			2011	0.21	0.18	0.25	1.13	1.08	1.18
			2012	0.25	0.22	0.30	1.13	1.09	1.18
			2013	0.24	0.20	0.28	1.15	1.10	1.20

Supplementary Table 15. Effect estimates for a pesticide ban

				Pesticide-involved suicides			Other suicide means		
Reference	Country	Means restriction	Year	Rate ratio	Lower 95% CI	Upper 95% CI	Rate ratio	Lower 95% CI	Upper 95% CI
Chowdhury et al., 2018	Bangladesh	Ban of WHO Class I (extremely and highly hazardous) pesticides in 2000	1996-2000 (mean)	-	-	-	-	-	-
			2001	1.00	0.98	1.03	1.00	0.98	1.02
			2002	0.88	0.85	0.91	0.87	0.85	0.90
			2003	0.76	0.72	0.79	0.76	0.73	0.79
			2004	0.71	0.68	0.75	0.73	0.70	0.77
			2005	0.75	0.70	0.80	0.73	0.69	0.77
			2006	0.77	0.72	0.83	0.70	0.66	0.75
			2007	0.79	0.72	0.86	0.76	0.71	0.82
			2008	0.73	0.66	0.80	0.75	0.69	0.82
			2009	0.66	0.60	0.73	0.70	0.64	0.77
			2010	0.62	0.55	0.69	0.70	0.63	0.77
			2011	0.56	0.50	0.64	0.68	0.60	0.76
			2012	0.52	0.45	0.60	0.70	0.62	0.79
			2013	0.46	0.40	0.53	0.69	0.61	0.79
			2014	0.43	0.37	0.50	0.70	0.61	0.81

Supplementary Table 16. Predicted age-standardized suicide mortality rate per 100,000 population and number of avoided deaths by suicide in seven countries in the Region of the Americas under a counterfactual scenario of a pesticide or firearm restriction implemented in 2020 for the years 2021-2030

Country	Sex	Scenario	2021	2022	2023	2024	2025	2026	2027	2028	2029	2030
**Pesticide ban**												
**El Salvador**	**Males**	**No restriction, age-standardized rate (95% CI)**	11.4 (11.0, 11.9)	11.7 (10.9, 12.5)	12.0 (10.8, 13.1)	12.2 (10.7, 13.7)	12.4 (10.7, 14.2)	12.6 (10.6, 14.6)	12.7 (10.6, 14.9)	12.8 (10.6, 15.0)	12.8 (10.5, 15.0)	12.7 (10.4, 14.9)
		**Means restriction, age-standardized rate (95% CI)**	10.0 (9.7, 10.3)	8.9 (8.5, 9.2)	8.6 (8.3, 9.1)	9.0 (8.5, 9.6)	9.1 (8.5, 9.7)	9.7 (9.0, 10.6)	9.4 (8.6, 10.3)	8.7 (8.0, 9.6)	8.5 (7.6, 9.4)	9.0 (7.1, 9.0)
		**Absolute number of avoided suicides (95% UI)**	-43	-85	-102	-99	-106	-92	-106	-131	-138	-153
	**Females**	**No restriction, age-standardized rate (95% CI)**	1.8 (1.5, 2.1)	1.8 (1.4, 2.1)	1.7 (1.3, 2.1)	1.6 (1.2, 2.0)	1.6 (1.2, 2.0)	1.5 (1.1, 1.9)	1.4 (1.0, 1.9)	1.4 (0.9, 1.8)	1.3 (0.8, 1.8)	1.2 (0.7, 1.8)
		**Means restriction, age-standardized rate (95% CI)**	1.6 (1.6, 1.7)	1.3 (1.3, 1.4)	1.2 (1.2, 1.3)	1.2 (1.1, 1.3)	1.2 (1.1, 1.2)	1.2 (1.1, 1.3)	1.1 (1.0, 1.2)	0.9 (0.8, 1.0)	0.9 (0.8, 0.9)	0.8 (0.7, 0.9)
		**Absolute number of avoided suicides (95% UI)**	-8	-14	-16	-14	-13	-11	-12	-15	-15	-16
**Guyana**	**Males**	**No restriction, age-standardized rate (95% CI)**	62.0 (53.7, 70.3)	61.2 (52.2, 70.2)	60.8 (51.5, 70.0)	60.5 (51.2, 69.9)	60.4 (51.1, 69.7)	60.3 (51.0, 69.7)	60.3 (50.9, 69.6)	60.3 (50.9, 69.6)	60.3 (50.9, 69.6)	60.2 (50.9, 69.6)
		**Means restriction, age-standardized rate (95% CI)**	54.2 (52.7, 56.1)	46.5 (44.4, 48.4)	43.8 (41.9, 46.2)	44.8 (42.1, 47.5)	44.4 (41.7, 47.7)	46.8 (43.1, 50.7)	44.6 (40.7, 48.8)	41.0 (37.4, 45.2)	39.8 (35.5, 44.0)	37.4 (33.1, 42.2)
		**Absolute number of avoided suicides (95% UI)**	-30	-57	-67	-62	-64	-55	-63	-79	-84	-95
	**Females**	**No restriction, age-standardized rate (95% CI)**	16.5 (15.3, 17.8)	16.6 (15.2, 18.0)	16.6 (15.2, 18.1)	16.7 (15.2, 18.2)	16.8 (15.1, 18.4)	16.8 (15.1, 18.5)	16.9 (15.0, 18.7)	16.9 (15.0, 18.8)	17.0 (15.0, 19.0)	17.0 (14.9, 19.1)
		**Means restriction, age-standardized rate (95% CI)**	14.5 (14.1, 15.0)	12.6 (12.0, 13.1)	11.9 (11.4, 12.6)	12.4 (11.6, 13.2)	12.6 (11.8, 13.5)	13.1 (12.1, 14.3)	12.4 (11.3, 13.6)	11.3 (10.3, 12.5)	10.9 (9.7, 12.1)	10.1 (9.0, 11.4)
		**Absolute number of avoided suicides (95% UI)**	-9	-17	-21	-19	-18	-16	-20	-25	-27	-31
**Nicaragua**	**Males**	**No restriction, age-standardized rate (95% CI)**	7.4 (6.6, 8.2)	7.3 (6.4, 8.1)	7.1 (6.2, 8.1)	7.0 (6.0, 8.0)	6.9 (5.8, 7.9)	6.8 (5.7, 7.9)	6.6 (5.5, 7.8)	6.5 (5.3, 7.7)	6.4 (5.1, 7.6)	6.2 (4.9, 7.6)
		**Means restriction, age-standardized rate (95% CI)**	6.5 (6.3, 6.7)	5.5 (5.3, 5.7)	5.2 (5.0, 5.5)	5.2 (4.9, 5.5)	5.0 (4.7, 5.3)	5.2 (4.8, 5.6)	4.9 (4.5, 5.4)	4.5 (4.1, 4.9)	4.3 (3.9, 4.8)	4.0 (3.6, 4.5)
		**Absolute number of avoided suicides (95% UI)**	-29	-56	-64	-61	-65	-54	-59	-72	-74	-81
	**Females**	**No restriction, age-standardized rate (95% CI)**	1.8 (1.5, 2.2)	1.8 (1.4, 2.1)	1.7 (1.3, 2.1)	1.6 (1.2, 2.0)	1.6 (1.1., 2.0)	1.5 (1.0, 2.0)	1.5 (1.0, 1.9)	1.4 (0.9, 1.9)	1.3 (0.8, 1.9)	1.3 (0.7, 1.8)
		**Means restriction, age-standardized rate (95% CI)**	1.6 (1.5, 1.6)	1.3 (1.3, 1.4)	1.2 (1.2, 1.3)	1.2 (1.1, 1.3)	1.2 (1.2, 1.2)	1.2 (1.1, 1.3)	1.1 (1.0, 1.2)	0.9 (0.9, 1.0)	0.9 (0.8, 1.0)	0.8 (0.7, 0.9)
		**Absolute number of avoided suicides (95% UI)**	-8	-15	-17	-16	-16	-13	-14	-17	-17	-19
**Suriname**	**Males**	**No restriction, age-standardized rate (95% CI)**	41.1 (39.8, 42.5)	41.0 (39.6, 42.5)	40.9 (39.4, 42.5)	40.8 (39.2, 42.5)	40.8 (39.0, 42.5)	40.7 (38.8, 42.5)	40.6 (38.6, 42.5)	40.5 (38.5, 42.5)	40.4 (38.3, 42.5)	40.3 (38.1, 42.6)
		**Means restriction, age-standardized rate (95% CI)**	36.0 (34.9, 37.3)	31.2 (29.7, 32.4)	29.3 (28.1, 31.0)	30.4 (28.5, 32.3)	30.5 (28.5, 32.8)	31.7 (29.1, 34.4)	29.9 (27.2, 32.7)	27.3 (24.8, 30.1)	26.1 (23.3, 28.9)	24.2 (21.5, 27.4)
		**Absolute number of avoided suicides (95% UI)**	-14	-28	-34	-31	-31	-27	-32	-40	-43	-49
	**Females**	**No restriction, age-standardized rate (95% CI)**	11.8 (11.4, 11.9)	11.7 (11.4, 11.9)	11.7 (11.4, 11.9)	11.6 (11.3, 11.9)	11.6 (11.3, 11.9)	11.6 (11.3, 11.9)	11.6 (11.2, 11.9)	11.6 (11.2, 11.9)	11.6 (11.2, 12.0)	11.5 (11.1, 12.0)

		**Means restriction, age-standardized rate (95% CI)**	10.3 (9.9, 10.6)	8.9 (8.4, 9.2)	8.3 (8.0, 8.8)	8.7 (8.1, 9.2)	8.8 (8.2, 9.4)	9.1 (8.3, 9.9)	8.5 (7.7, 9.3)	7.8 (7.1, 8.6)	7.4 (6.6, 8.2)	6.8 (6.1, 7.7)
		**Absolute number of avoided suicides (95% UI)**	-4	-8	-10	-9	-9	-8	-10	-12	-13	-15
**Trinidad and Tobago[a]**	**Males**	**No restriction, age-standardized rate (95% CI)**	10.4 (8.6, 12.3)	9.8 (7.8, 11.8)	9.1 (7.0, 11.2)	8.5 (6.2, 10.7)	7.8 (5.4, 10.2)	7.2 (4.6, 9.7)	6.5 (3.8, 9.2)	5.8 (3.1, 8.6)	5.2 (2.3, 8.1)	4.5 (1.5, 7.6)
		**Means restriction, age-standardized rate (95% CI)**	9.1 (8.9, 9.4)	7.4 (7.1, 7.7)	6.6 (6.3, 7.0)	6.2 (5.9, 6.6)	5.6 (5.3, 6.0)	5.5 (5.1, 5.9)	4.8 (4.4, 5.3)	4.0 (3.7, 4.5)	3.5 (3.2, 3.9)	3.0 (2.6, 3.3)
		**Absolute number of avoided suicides (95% UI)**	-10	-18	-19	-17	-17	-13	-13	-14	-13	-12
	**Females**	**No restriction, age-standardized rate (95% CI)**	3.4 (2.3, 4.4)	3.2 (2.0, 4.4)	3.1 (1.7, 4.4)	2.9 (1.4, 4.4)	2.8 (1.2, 4.4)	2.6 (0.9, 4.3)	2.5 (0.7, 4.3)	2.3 (0.4, 4.3)	2.2 (0.2, 4.2)	2.1 (0, 4.2)
		**Means restriction, age-standardized rate (95% CI)**	2.9 (2.9, 3.0)	2.4 (2.3, 2.5)	2.2 (2.1, 2.4)	2.1 (2.0, 2.3)	2.0 (1.9, 2.1)	2.0 (1.9, 2.2)	1.9 (1.7, 2.0)	1.6 (1.5, 1.8)	1.5 (1.4, 1.7)	1.4 (1.2, 1.5)
		**Absolute number of avoided suicides (95% UI)**	-3	-5	-6	-5	-6	-4	-4	-5	-5	-5
**Firearm ban**												
**United States of America**	**Males**	**No restriction, age-standardized rate (95% CI)**	22.2 (21.5, 22.9)	22.5 (21.7, 23.2)	22.8 (22.0, 23.6)	23.1 (22.2, 23.9)	23.4 (22.5, 24.3)	23.7 (22.7, 24.6)	23.0 (22.9, 25.0)	24.3 (23.2, 25.3)	24.6 (23.4, 25.7)	24.8 (23.7, 26.0)
		**Means restriction, age-standardized rate (95% CI)**	20.0 (18.8, 21.4)	19.3 (18.1, 20.6)	18.7 (17.5, 20.1)	17.6 (16.4, 18.9)	18.6 (17.4, 20.0)	17.1 (15.9, 18.3)	15.7 (14.6, 16.9)	15.2 (14.2, 16.4)	14.7 (13.6, 15.8)	15.7 (14.6, 16.9)
		**Absolute number of avoided suicides (95% UI)**	-3,432	-5,102	-6,538	-8,855	-7,655	-10,690	-13,397	-14,665	-16,077	-14.911
	**Females**	**No restriction, age-standardized rate (95% CI)**	6.9 (6.7, 7.0)	7.0 (6.9, 7.2)	7.2 (7.0, 7.3)	7.3 (7.1, 7.5)	7.4 (7.3, 7.6)	7.6 (7.4, 7.8)	7.7 (7.5, 7.9)	7.9 (7.7, 8.1)	8.0 (7.8, 8.2)	8.1 (7.9, 8.4)
		**Means restriction, age-standardized rate (95% CI)**	7.6 (7.2, 8.0)	7.6 (7.3, 8.1)	7.3 (6.9, 7.7)	7.1 (6.7, 7.5)	7.4 (7.0, 7.8)	6.9 (6.6, 7.4)	6.5 (6.1, 6.9)	6.4 (6.1, 6.8)	6.4 (6.0, 6.7)	6.6 (6.2, 7.0)
		**Absolute number of avoided suicides (95% UI)**	1,272	1,055	255	-386	-147	-1,065	-2,065	-2,457	-2,802	-2,673
**Uruguay[a]**	**Males**	**No restriction, age-standardized rate (95% CI)**	29.8 (26.9, 32.7)	30.2 (27.0, 33.3)	30.5 (27.2, 33.8)	30.9 (27.3, 34.4)	31.2 (27.5, 35.0)	31.6 (27.6, 35.6)	31.9 (27.7, 36.1)	32.3 (27.9, 36.7)	32.7 (28.0, 37.3)	33.0 (28.2, 37.9)
		**Means restriction, age-standardized rate (95% CI)**	35.3 (33.6, 37.1)	35.4 (33.7, 37.2)	33.4 (31.7, 35.1)	32.2 (30.6, 33.9)	33.0 (31.4, 34.8)	31.2 (29.7, 32.9)	29.1 (27.6, 30.7)	28.6 (27.1, 30.1)	28.3 (26.8, 29.8)	28.8 (27.3, 30.3)
		**Absolute number of avoided suicides (95% UI)**	88	84	46	22	29	-6	-46	-60	-71	-69
	**Females**	**No restriction, age-standardized rate (95% CI)**	7.6 (6.8, 8.3)	7.6 (6.8, 8.4)	7.7 (6.9, 8.6)	7.8 (6.9, 8.7)	7.9 (6.9, 8.8)	8.0 (7.0, 9.0)	8.1 (7.0, 9.1)	8.1 (7.0, 9.3)	8.2 (7.1, 9.4)	8.3 (7.1, 9.5)
		**Means restriction, age-standardized rate (95% CI)**	9.6 (9.1, 10.0)	9.7 (9.2, 10.1)	9.1 (8.6, 9.5)	8.8 (8.4, 9.2)	9.0 (8.5, 9.4)	8.5 (8.1, 8.9)	7.9 (7.6, 8.3)	7.8 (7.4, 8.2)	7.8 (7.4, 8.2)	7.8 (7.4, 8.2)
		**Absolute number of avoided suicides (95% UI)**	33	34	22	16	18	9	-2	-5	-8	-8

[a]Included in counterfactual scenario 2 only (i.e., a means restriction (firearm or pesticide ban) for those countries where the respective means accounted for 40% or more of overall suicides for at least one sex).
